# Site-specific albumin tagging with NIR-II fluorogenic dye for high-performance and super-stable bioimaging

**DOI:** 10.7150/thno.88815

**Published:** 2024-02-24

**Authors:** Ningning Zhu, Jiajun Xu, Qi Su, Tianyang Han, Ding Zhou, Yuewei Zhang, Shoujun Zhu

**Affiliations:** 1 Joint Laboratory of Opto-Functional Theranostics in Medicine and Chemistry, First Hospital of Jilin University, Jilin University, Changchun 130021, P.R. China.; 2 State Key Laboratory of Supramolecular Structure and Materials, Center for Supramolecular Chemical Biology, College of Chemistry, Jilin University, Changchun 130012, P.R. China.; 3 School of Chemistry and Pharmaceutical Engineering, Jilin Institute of Chemical Technology, Jilin 132022, P.R. China.; 4 Jilin Provincial Key Laboratory of Tooth Development and Bone Remodeling, School and Hospital of Stomatology, Jilin University, Changchun 130021, P.R. China.

**Keywords:** NIR-II cyanine dye, cyanine@albumin, covalent binding site, site-specific labeling, NIR-II lymphography

## Abstract

Synthetic near-infrared-II (NIR-II) dyes are promising for deep tissue imaging, yet they are generally difficult to target a given biomolecule with high specificity. Furthermore, the interaction mechanism between albumin and cyanine molecules, which is usually regarded as uncertain "complexes" such as crosslinked nanoparticles, remains poorly understood.

**Methods:** Here, we propose a new class of NIR-II fluorogenic dyes capable of site-specific albumin tagging for in situ albumin seeking/targeting or constructing high-performance cyanine@albumin probes. We further investigate the interaction mechanism between NIR-II fluorogenic dyes and albumin.

**Results:** We identify CO-1080 as an optimal dye structure that produces a stable/bright NIR-II cyanine@albumin probe. CO-1080 exhibits maximum supramolecular binding affinity to albumin while catalyzing their covalent attachment. The probe shows exact binding sites located on Cys476 and Cys101, as identified by proteomic analysis and docking modeling.

**Conclusion:** Our cyanine@albumin probe substantially improves the pharmacokinetics of its free dye counterpart, enabling high-performance NIR-II angiography and lymphography. Importantly, the site-specific labeling tags between NIR-II fluorogenic dyes and albumin occur under mild conditions, offering a specific and straightforward synthesis strategy for NIR-II fluorophores in the fields of targeting bioimaging and imaging-guided surgery.

## Introduction

Near-infrared-II (NIR-II, 900‒2000 nm) fluorescence imaging is a promising imaging modality for deep tissue visualization 1-16. Small molecular dyes with either NIR-II peak emission or off-peak emission have been extensively developed for NIR-II imaging. Currently, to create targeted contrast agents, NIR-I/II probes rely on the covalent attachment of NIR-II dyes (bearing bioconjugatable moieties) to a protein of interest with a reactive group (e.g., primary amino). One successful case of a NIR-targeted contrast agent for clinical use is IRDye800CW-labeled antibodies, such as cetuximab/panitumumab@IRDye800CW, which were designed to target the epidermal growth factor receptor (EGFR) 17-19. The NIR-dye-labeled antibodies have facilitated either therapeutic or detection applications in the clinic, including tumor-margin detection, identification of tumor-positive lymph nodes (LNs), and imaging-guided surgery 20-24. However, the labeling process using IRDye800CW-NHS-ester results in random conjugation with primary amino on the antibody. Given that the number and position of fluorescent labels dramatically affect the targeting ability of antibodies 5, 25-28, it is critical to develop site-specific labeling strategies to achieve an ideal NIR-targeted agent with maximizing the targeting efficiency and brightness.

To achieve effective site-specific labeling of NIR dyes on proteins, it is necessary introducing highly reactive groups (such as maleimide) on the dye scaffolds 6, 29, 30. Further adopting cysteine residues on the selected position of proteins would facilitate the accurately-scheduled nucleophilic substitution under the physiological conditions. However, this strategy is laborious and costly. In addition, the designed reaction sites are generally located on the surface of the applied protein, so the tethered dye is exposed in the aqueous solution, leading to considerable issues of photostability and vibration/rotation non-radiative transition process. Since 2010, Chung 31, Shi 32, 33, and other groups reported that cyanine dyes with a central 4-chlorocyclohexly ring could accumulate and persist in solid tumors. Consequently, Prof. Burgess 34-38 and Prof. Goncalves 39 proposed that these dyes with meso-Cl could directly label cysteine residues of albumin under mild conditions (e.g., 37^o^C in PBS buffer) 34, 35, 39. They attributed the tumor-seeking properties of these dyes to albumin-mediated processes.

Our recent research has investigated the precise binding site between Cl-containing dyes and albumin (HSA) using liquid chromatography high-resolution mass spectrometry (LC-HRMS) and shotgun proteomics techniques 40. Our findings suggest that Cl-containing dyes (e.g., IR-780, IR-808, IR-783) can bind to albumin through supramolecular interactions, followed by covalently binding through the nucleophilic substitution reaction at Cys476 residue on Domain III (DIII). The efficient confinement effect of Cl-containing dyes in the hydrophobic pocket of albumin would substantially improve the photostability and radiative transition process of embedded dyes. The binding efficiency between Cl-containing dyes and albumin varies with dye structures. Current researches on this topic exclusively focus on NIR-I dye structures with emission at around 800 nm. Since the pioneering work of Dai, Zhang, and Cheng in 2017/2018 41, 42, the development of NIR-II dye@protein complex with enhanced brightness has been continuously exploited 40, 43-52. Despite the promising results, the detailed binding mechanism was rarely confirmed.

Chemogenetic/fluorogenic systems have been developed that use synthetic labels to target genetically encoded tags 53, 54. While promising, these systems have not yet been expanded to NIR-II dyes with peak emissions over 1000 nm. Herein, we synthesized a set of fluorogenic NIR-II dyes (1080 dyes: Et-1080, St-1080, FD-1080-Cl, and CO-1080) and investigated the binding mechanism (site) between NIR-II fluorogenic dyes and albumin 42, 55-57. We confirmed that CO-1080 displayed the strongest binding ability with albumin, coupled with the highest brightness enhancement compared to other FD-1080 analogues. We successfully identify the two exact binding sites (Cys476 and Cys101) for cyanine dyes. One site was located on domain IIIa (DIIIa) with efficient spatial restriction, thus significantly enhancing the brightness of 1080 dyes. While the other was located on domain Ia (DIa) with insufficient supramolecular restriction. Rational engineering the dye structure and tagging with a conformation-favorable protein pocket can combine to maximize the stability and brightness of CO-1080@albumin. The site-specific albumin tagged with fluorogenic dye is promising for in vitro constructing high-performance and super-stable dye@albumin probes as well as in-situ albumin targeting.

## Results and Discussion

### Synthesis and characterization of fluorogenic 1080 dyes

**Figure 1:**
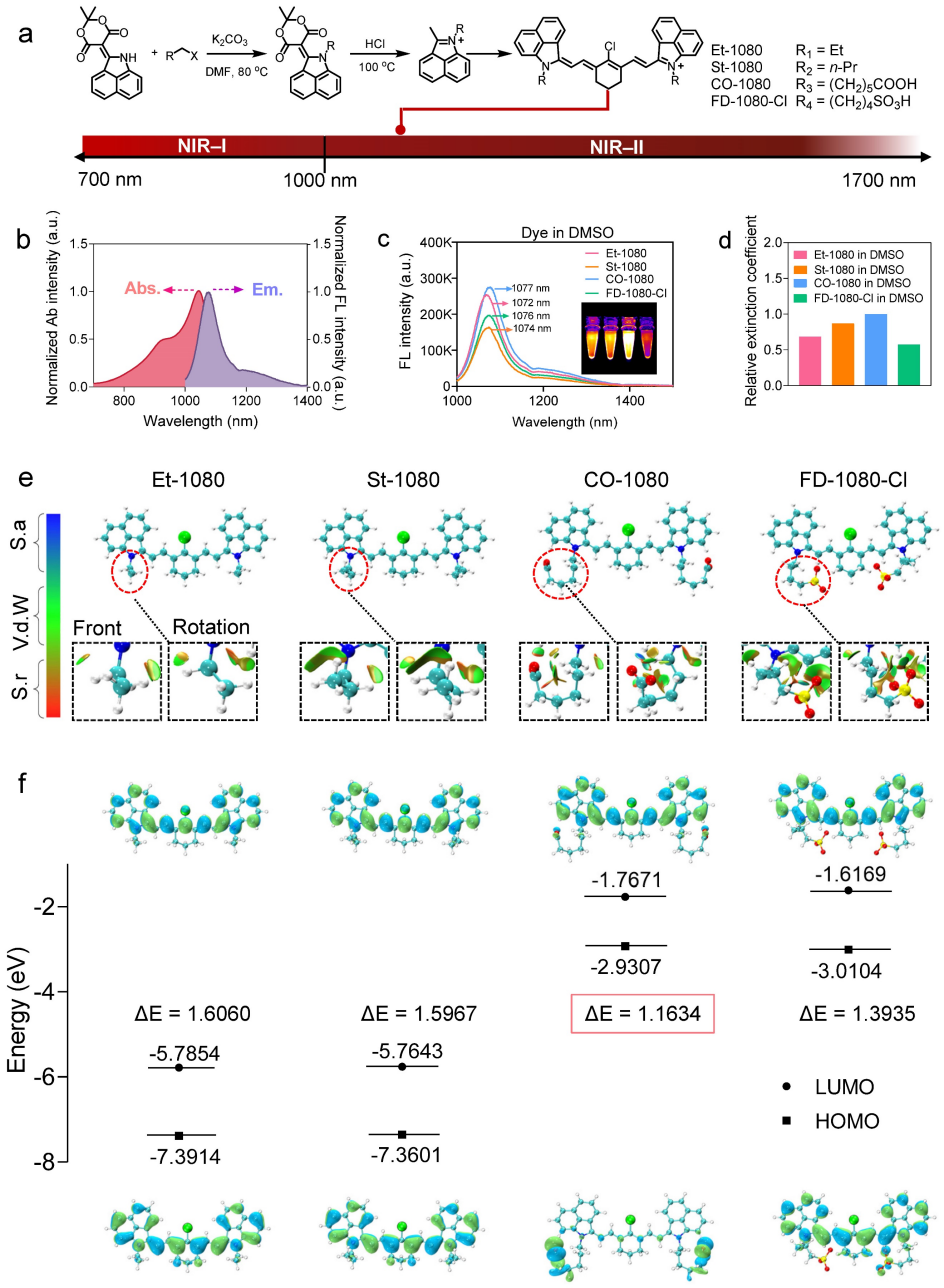
**Panel of 1080 dyes and their photophysical properties.** (**A**) Synthetic route of NIR-II cyanine dyes. (**B**) Normalized absorbance and emission spectra of cyanine dyes. Abs.: absorption spectra; Em.: emission spectra. (**C**) Fluorescence emission spectra of cyanine dyes in DMSO under 980 nm excitation. (**D**) Using CO-1080 as a reference, the relative molar extinction coefficients of cyanine dyes in DMSO. (**E**) Optimal molecular conformation of Et-1080, St-1080, CO-1080, and FD-1080-Cl. S.a: Strong attraction (H-bond and halogen-bond); Vd.W: Van der Waals interaction; S.r: Strong repulsion (steric effect in ring and cage). (**F**) Illustration of HOMO and LUMO energy levels of Et-1080, St-1080, CO-1080, and FD-1080-Cl based on density function theory. The HOMO and LUMO energy levels were plotted based on the optimized S0 and S1 geometries using Gaussian (b31yp/6-31g(d).

NIR-II dye with exceptional photostability and long absorption/emission wavelength is essential for long-term visualization of biological events 42. Building on previous publications in terms of FD-1080 and chemical structure features of NIR-I cyanine dyes (IR-780, IR-808, and IR-783) with the optimal albumin-binding ability 36, 37, 39, 40, 43, 44, 55, 56, we designed fluorogenic FD-1080 analogues to achieve site-specific albumin tagging and investigate the binding mechanism of NIR-II dyes and albumin. Compared to the pioneering work of FD-1080, we developed an alternate strategy to synthesize FD-1080 analogues including alkylation and deprotection, followed by the Knoevenagel reaction (Figure 1A and Figure S1-6) 58, 59. Tuning a pair of naphthalene structural isomers (alkyl, carboxylic acid, or sulphonic terminal groups on indole rings) resulted in FD-1080 analogues (Et-1080, St-1080, CO-1080, and FD-1080-Cl) with maximal excitation and emission wavelengths in the NIR-II region (> 1000 nm), respectively (Figure 1B).

The photophysical properties such as absorbance, fluorescence spectra, brightness, the relative molar extinction coefficients, and NIR-II quantum yields of the 1080 dyes were investigated in detail. We characterized the photophysical properties of four dyes in DMSO, finding that they have absorption/emission in NIR-II regions (Figure 1C and Figure S7). Plotting the NIR-II brightness of dyes in DMSO revealed that CO-1080 exhibited a significant fluorescence enhancement compared to other analogues (Figure S8A). While all four 1080 compounds show similar optical properties, CO-1080 displayed slight bathochromic shifts with λ_max em_ = 1077 nm, affording a larger Stokes shift compared with Et-1080, St-1080, and FD-1080-Cl. Notably, CO-1080 exhibited a larger molar extinction coefficient and higher quantum yield compared with other FD-1080 analogues (Figure 1D, Table S1, and Figure S9).

To better understand the luminescence behavior of FD-1080 analogues, we calculated the HOMO and LUMO, as well as the restrained electrostatic potential (RESP) charges of each molecule, which are very important for subsequent molecular dynamics simulations. Conformational searching was performed on the flexible structure of these molecules to identify their lowest energy structures. Specifically, they were subjected to high-temperature dynamics sampling followed by progressively refining more accurate computational methods, thus obtaining the final lowest energy conformer (see Experimental Methods section, Figure 1E). To further verify the optimality of these structures, we performed a weak interaction analysis using the reduced density gradient (RDG) method on the optimal conformation. As shown in Figure 1E, we observed that Et-1080 and St-1080 molecules orient their two alkyl chains in opposite directions to reduce steric hindrance, while FD-1080-Cl molecules with two sulfonic acid-containing side chains tend to aggregate towards the center to form more robust hydrogen binding interactions. In contrast, CO-1080 molecules with two carboxyl-containing side chains are oriented away from each other, attributed to the longer side chains hindering their ability to form hydrogen bonds with the hydrogen atoms on the cyclohexenyl. By orienting the two side chains in opposite directions, as shown in Figure 1E, strong hydrogen bonds are facilitated while reducing electrostatic repulsion interactions. Based on these optimal structures, we calculated the HOMO and LUMO for each molecule (Figure 1F) 60, 61, with CO-1080 having the smallest bandgap of 1.16 eV, consistent with its near-infrared fluorescence. Although the series of NIR-II cyanine fluorophores showed bright and sharp NIR-II emission in DMSO, their brightness and emission spectra suffered from attenuation and broadening in an aqueous solution (Figure S8b, S9). Therefore, maintaining superior NIR-II brightness and favorable anti-quenching capability in aqueous solution remains a significant challenge so far.

### Fluorogenic NIR-II dyes constructed 1080@bovine serum albumin (BSA) probe by in vitro albumin tagging

**Figure 2:**
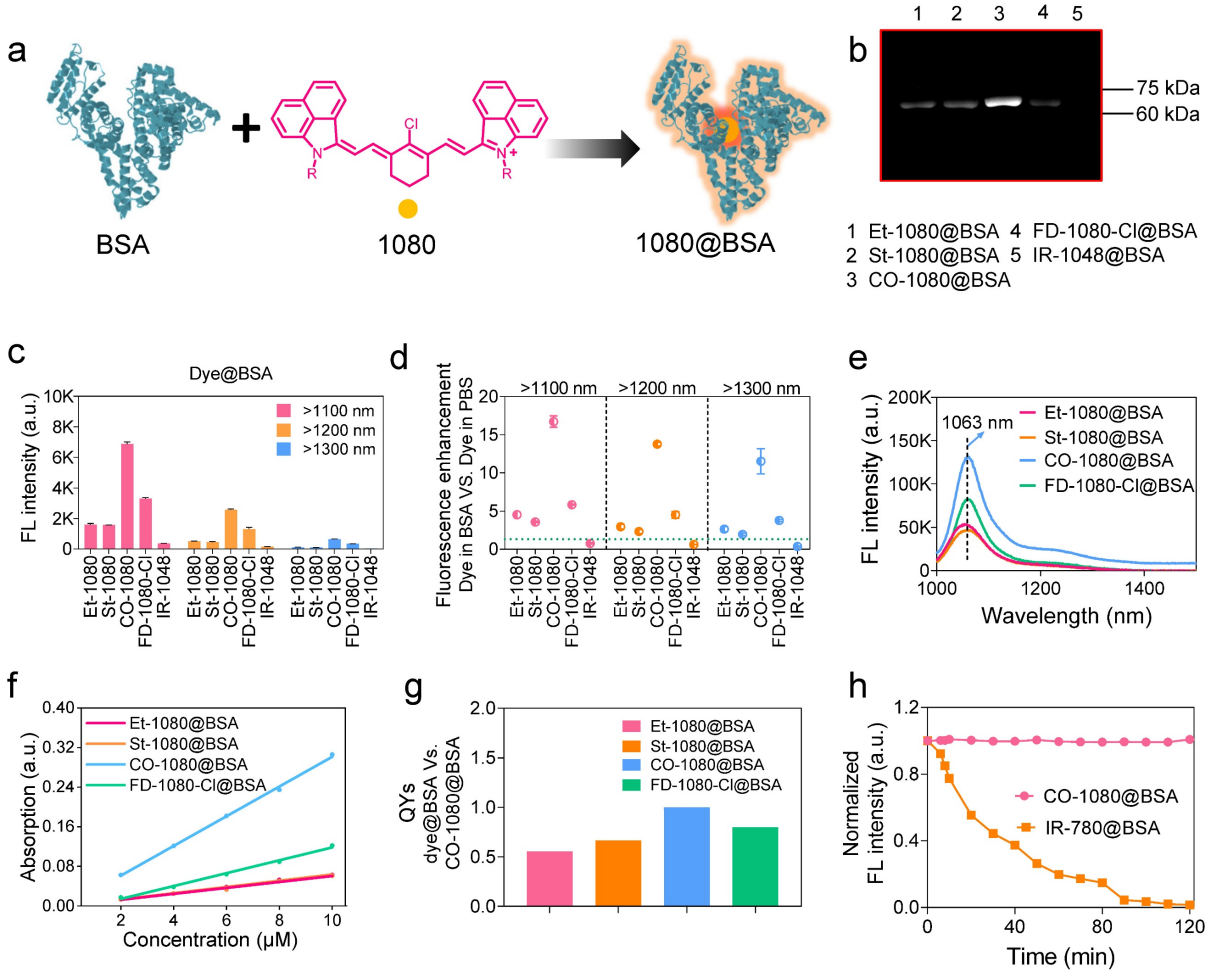
**1080@BSA probes and their photophysical properties.** (**A**) Diagram of the 1080@BSA probes. (**B**) Electrophoresis analysis of 1080@BSA probes, including Et-1080@BSA, St-1080@BSA, CO-1080@BSA, and FD-1080-Cl@BSA (IR-1048@BSA was used as a control). (**C**) Fluorescence intensity of four 1080@BSA probes under different emission filters at the same exposure time. (**D**) Fluorescence enhancement of 1080@BSA probes compared to their corresponding free dyes in PBS buffer. Imaging conditions: 10 μM, > 1100 nm collection. (**E**) Fluorescence emission spectra of four 1080@BSA probes. (**F**) Comparison of molar extinction coefficients of 1080@BSA probes. (**G**) Relative quantum yields of four 1080@BSA probes using CO-1080@BSA as a reference (normalized as 1). (h) Photostability of CO-1080@BSA and IR-780@BSA under continuous laser irradiation for 120 minutes.

We have revealed that the hydrophobic pocket of albumin can effectively embed NIR-I cyanine dyes through covalent binding, preventing nonradiative transition caused by internal rotation/vibration of dye 40, 46. Such site-specific binding of NIR dyes on protein not only substantially improves the brightness of NIR dyes in physiological conditions, but also provides an accurately-scheduled dye-labeling strategy. To expand this fluorogenic strategy to NIR-II dyes, we conducted a systematic investigation of the binding mechanism between the synthesized 1080 dyes (with commercial IR-1048 as a control dye) and albumin (Figure 2A). Our goal was to determine the optimal reaction conditions for 1080@BSA probes using NIR-II brightness and electrophoresis analysis (see Supplementary Material for detailed protocols). Results revealed that the optimal reaction conditions include a reaction temperature of 50^o^C for CO-1080@BSA and 60^o^C for other 1080@BSA probes (Figure S10), a reaction ratio of 1:1 (Figure S11), a time of 2 h (Figure S12), and a concentration of 10 μM (Figure S13, S14).

Following the optimal reaction conditions, the NIR-II fluorescent bands were observed at the corresponding molecular weight positions on the electrophoresis gel when the 1080 dyes interacted with BSA. This verified the efficient covalent binding between the 1080 dyes and BSA. Among all the 1080@BSA compounds, the CO-1080@BSA displayed the strongest covalent binding ability (Figure 2B). We then examined the brightness of cyanine@BSA under > 1100, > 1200, and > 1300 nm sub-NIR-II windows and found that the CO-1080@BSA had the highest brightness under all the tested windows (Figure 2C). Notably, the quantified results showed that the NIR-II brightness of CO-1080@BSA was ∼17-fold higher than that of CO-1080 in PBS buffer, while maintaining nearly 20% brightness compared to CO-1080 in DMSO (Figure 2D, and Figure S8b, C). Compared with free 1080 dyes, dye@BSA probes displayed a significant bathochromic shift in PBS buffer with stable peak absorption/emission (Figure 2E, Figure S7). The fluorescence enhancement of dye@BSA probes was potentially attributed to the internal rotation/vibration suppression of dye molecules 40, 44, 46. The absorption spectra of CO-1080 showed a 1.5-fold increase in BSA solution compared to DMSO, while the absorption spectra of the other dyes showed various degrees of reduction in BSA solution versus that of DMSO at the same concentration (Figure S15). Moreover, CO-1080@BSA exhibited a larger molar extinction coefficient and higher quantum yield than other 1080@BSA probes (Figure 2F, G, and Figure S16), collectively resulting in superior NIR-II brightness.

We also tested the photostability for all 1080 dyes in either DMSO or their BSA-bound probes with continuous 980 nm laser irradiation (Figure S17). Results indicated that the half-life of brightness decay for all tested dyes in DMSO was less than 78 min. The order of photostability of dyes in DMSO was as follows: FD-1080-Cl > CO-1080 > St-1080 > Et-1080. Conversely, the brightness of all dye@BSA probes remained constant even for 120 min under NIR-II window, indicating that the combination with BSA significantly improved their photostability. Compared to IR-780@BSA with considerable photobleaching ability 40, 43, CO-1080@BSA will be applicable not only for non-invasive long-term visualization of deep-tissue-based events but also for accurate NIR-II molecular imaging under either macroscopical or microscopical manners (Figure 2H).

### Binding sites between 1080 dyes and BSA

**Figure 3:**
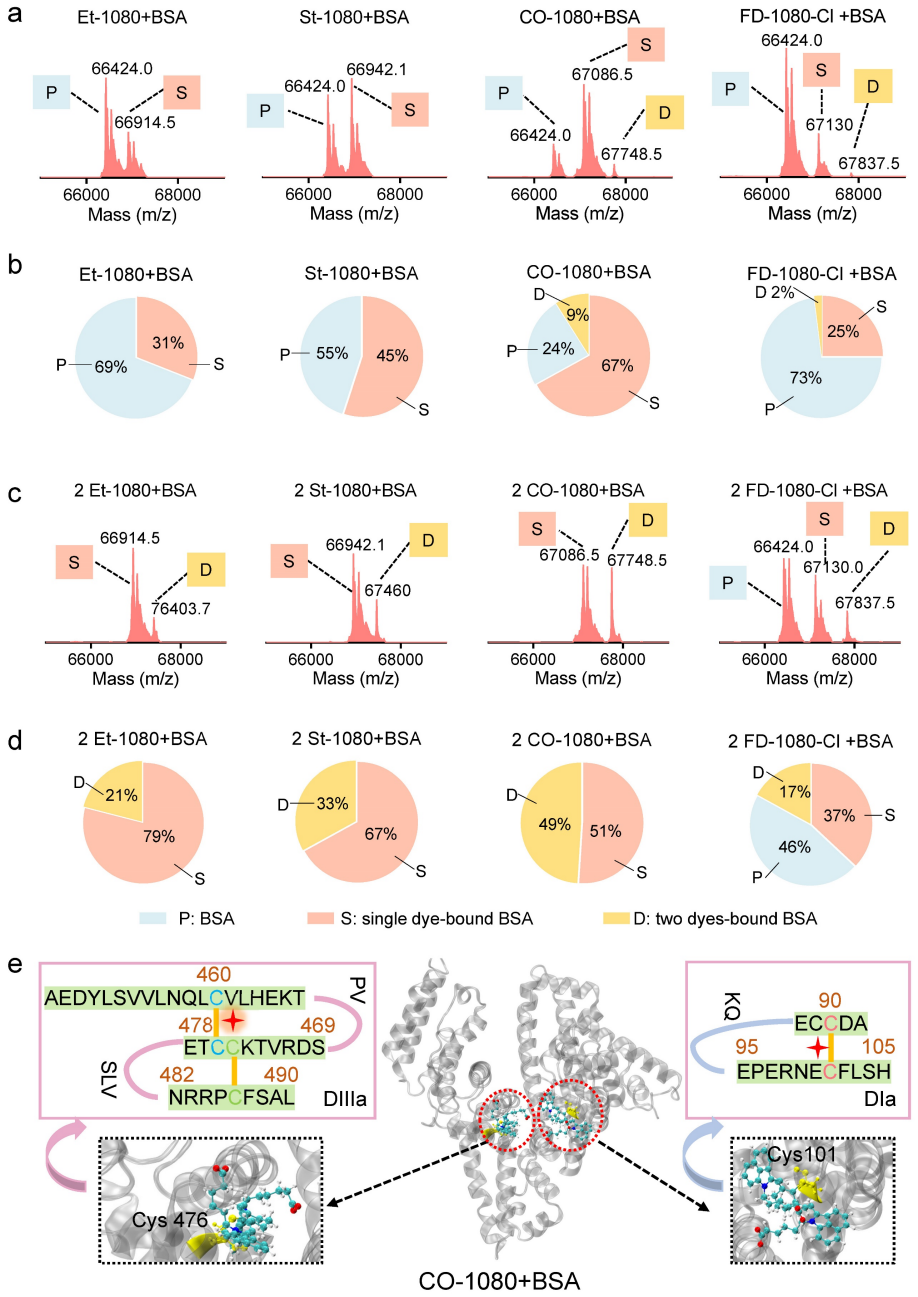
** LC-HRMS analysis and proteomics analysis of 1080@BSA probes.** LC-HRMS of Et-1080@BSA, St-1080@BSA, CO-1080@BSA, and FD-1080-Cl@BSA after (**A**) the dye reacted with BSA at a 1:1 ratio or (**C**) at 2:1 ratio. Quantification of molecular weights corresponding to free BSA, one 1080 labeled BSA, and two 1080 labeled BSA after dye (**B**) 1:1 or (**D**) 2:1 reacting with BSA. (**E**) Center: 3D structure of the exact binding site for CO-1080 in BSA; Left and right: Full BSA contains two binding sites for CO-1080: one site is Cys101 on DIa while the other is located in the hydrophobic pocket of DIIIa (Cys476).

We have electrophoretically confirmed that 1080 dyes bind to albumin by covalent binding, with an optimal reaction ratio close to 1:1 or 1:2 based on maximum brightness screening. The binding mechanism was further investigated through liquid chromatography high-resolution mass spectrometry (LC-HRMS). Surprisingly, considerable differences in binding behaviors for each 1080 dye were observed, with BSA molecule covalently binding two CO-1080 and FD-1080-Cl molecules even in a 1:1 reaction ratio, while a large percentage of unbound BSA was left for Et-1080, St-1080, and FD-1080-Cl (**Figure 3A, B** and **Figure S18**). A shift in reaction ratio from 1:1 to 2:1 resulted in nearly all BSA molecules being bound by 1080 dyes, except for FD-1080-Cl, while the percentage of two dyes-bound BSA substantially increased (**Figure 3C, D**). Collectively, based on the quantified binding percentages in **Figure 3A-D**, the covalent binding affinity of four 1080 dyes with BSA were ranked in the following order: CO-1080 > St-1080 > Et-1080 > FD-1080-Cl. Interestingly, it appears that two independent binding sites exist for 1080 dyes on BSA.

Inspired by our previous reports that chlorine-containing cyanine dyes could react with cysteine (Cys) residue of albumins through nucleophilic substitution 40, we sought to validate the covalent formation via displacement of chloride of 1080 dyes by thiol group on cysteine. After mixing L-cysteine molecules (10:1 reaction ratio, room temperature) with Et-1080, St-1080, CO-1080, and FD-1080-Cl, the resulting products were directly identified using LC-HRMS (**Figure S19**). Results indicated that the thiol group of L-cysteine can react with meso-chlorine on the unique carbon of cyclohexenyl ring via nucleophilic substitution. However, this displacement of meso-chlorine is heavily reliant on the base catalyst, whereas the reaction of 1080 with BSA could be completed in mild conditions (e.g., PBS buffer). Therefore, the previous claim that the binding site of NIR-dyes at Cys34 (free thiol) in albumin is unlikely reasonable, as this nucleophilic substitution requires a conformationally favorable pocket to trigger the reaction under the mild aqueous condition.

To identify nucleophilic reaction sites between CO-1080 dye and protein residues, we conducted an unbiased shotgun proteomic analysis. The reaction product of BSA and CO-1080 (1:1) was first digested by trypsin, which efficiently and specifically cleaved the C-terminus of lysine and arginine. The resulting peptides were then analyzed by ultra-performance liquid chromatography/tandem mass spectrometry (UPLC-MS/MS). The raw MS files were analyzed and searched against the UniProt human database with dye labeling as a variable modification (C_38_H_46_N_2_O_6_S_2_, mass = 690.2797 m/z) using Byonic. The full mass (MS1) of the peptide was calculated to be 562.600 (m/z, z = 3, mass error < 13.5 ppm). We further inferred the residue composition of peptide and the exact binding residue by analyzing the mass distribution of b and y series ions. The measurement accuracy for the fragments was < 0.02 Da (y ion series; y1-y8). The MS and MS/MS spectra of results indicated that the dye-labeled peptide sequence was CC[+661.307]TESLVNR, revealing that the Cys476 residue in the DIIIa domain was the potential binding site. In addition, analyzing the fragment ion spectra of NEC[+661.307]FLSHKDDSPDLPK also detected Cys101 in DIa to be tagged with the dye (**Figure S20** and **Table S2**).

The 3D crystal structures of BSA revealed that the DIIIa and DIa domain contains disulfide bonds, which include the Cys476 and Cys460 residues as well as the Cys101 and Cys90 residues. We hypothesized that during the process of peptide folding into well-defined secondary structures, these disulfide bonds are reversible. Therefore, free -SH groups of Cys476 and Cys101 would be present in the hydrophobic pocket. Our analysis suggests that the Cl-C group in 1080 series dyes could react with the thiol group in Cys476/Cys101 through a nucleophilic substitution reaction. The 1080 dye preferentially reacted with the first binding site at 1:1 ratio, while both binding sites tend to be labeled at higher reaction ratios (e.g., 2:1 ratio) (**Figure 3E**).

### Docking modeling reveals interaction discrepancy between fluorogenic 1080 dyes and BSA

**Figure 4:**
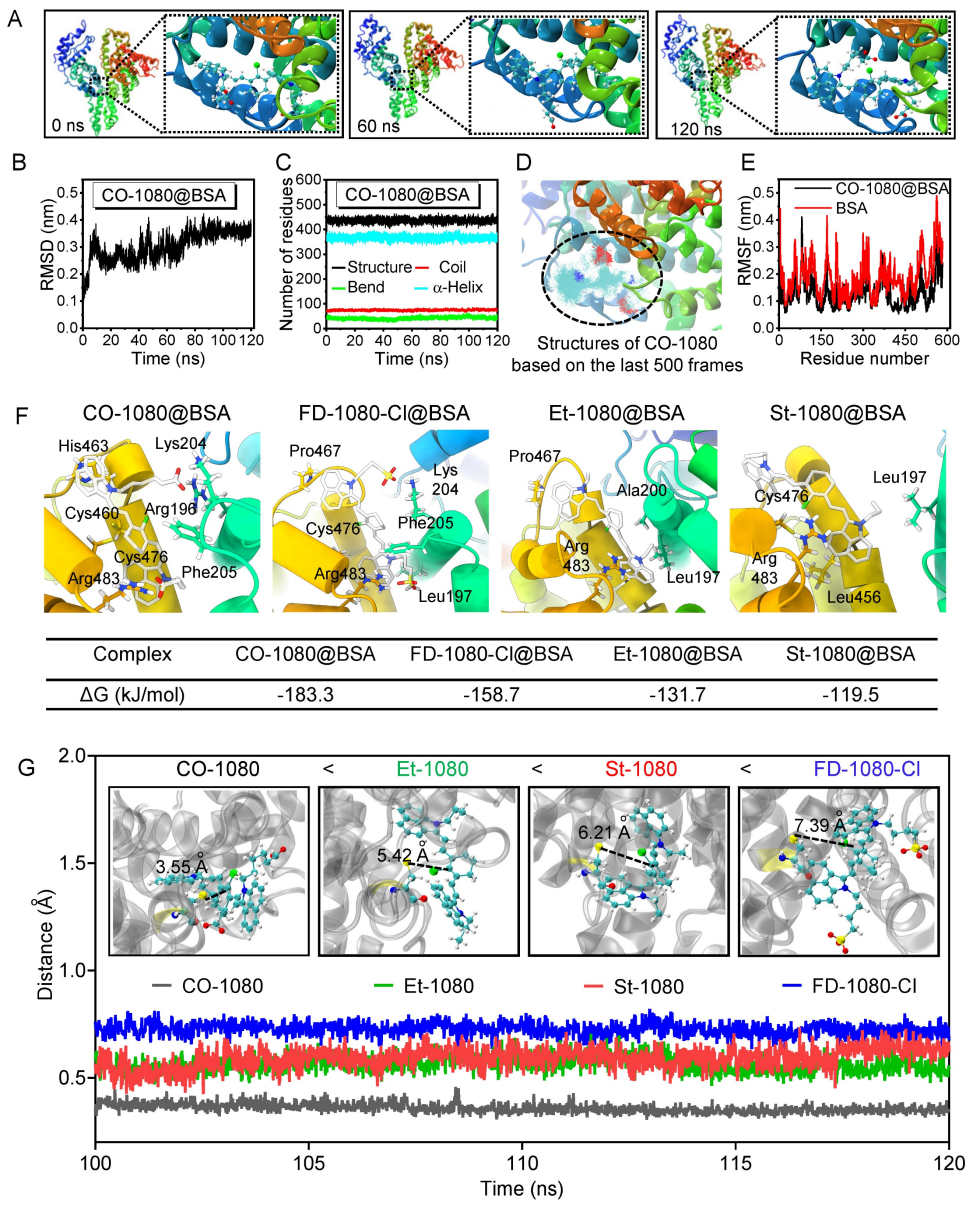
** Docking modeling of 1080@BSA.** (**A**) The structures of CO-1080 binding to the BSA complex were extracted from the molecular dynamics simulation trajectory at 0, 60, and 120 ns, respectively. (**B**) RMSD of CO-1080@BSA as a function of time. (**C**) The numbers of residues in diverse secondary structures of CO-1080@BSA. (**D**) RMSF values of amino acid residues 300-600 in CO-1080@BSA and BSA, respectively. (**E**) Conformational superposition of CO-1080@BSA. (**F**) 2D diagram of interaction between CO-1080, FD-1080-Cl, Et-1080, St-1080 and BSA. Interactions involved in the binding of BSA to amino acid residues are shown as squares in different corresponding colors; Contribution of individual residues to 1080 dyes binding energy. (**G**) The distances between the C atom in the Cl-C group of the dyes and the S atom in Cys476.

To investigate the interaction and binding poses between the fluorogenic dyes and BSA, molecular docking modeling was performed. Since molecular docking is a semi-flexible docking process, we first confirmed the effective binding modes using 120 ns molecular dynamics simulations (MD) (**Figure 4A, Figure S21, 22,** and **Movie S1**). We calculated the root mean square deviation (RMSD) of the skeleton atoms in the four complexes using the entire trajectory to confirm structural stability after a least-squares fit to the initial structure. The RMSD values of Et-1080@BSA, St-1080@BSA, CO-1080@BSA, and FD-1080-Cl@BSA reached equilibrium from 90 ns with minor fluctuations (**Figure 4B** and **Figure S23**). The numbers of residues in diverse secondary structures of the recombinant albumin remained almost unchanged during the 120 ns simulation (**Figure 4C** and **Figure S24**), indicating that the existence of the Et-1080, St-1080, CO-1080, and FD-1080-Cl ligands had no adverse effects on the physicochemical properties of BSA. We then focused on CO-1080 and examined the structure of its final 500 frames in the trajectory. The results showed that the conformation of CO-1080 remained almost unchanged even in the final stage of the simulation, indicating its stable interaction with BSA (**Figure 4D**). We further detected residue-wise root mean square fluctuation (RMSF), reflecting the position deviation of amino acid residues in BSA. As shown in **Figure 4E**, the RMSF values of the CO-1080@BSA complex were significantly lower than those of the same region in free BSA, indicating that the amino acid residues at the binding site of the ligand CO-1080 were inhibited, thus resulting in multiple interactions between CO-1080 and BSA.

To further characterize the detailed binding patterns and differences in binding ability between the dyes and BSA, we visualized the final structure of the simulation trajectory using the Visual Molecular Dynamics (VMD) program, and displayed the three-dimensional (3D) and two-dimensional (2D) interaction diagrams (**Figure 4F** and **Movie S2**). Due to the structural characteristics of the four molecules, various weak interactions occurred between 1080 dyes and BSA. From a qualitative perspective, it can be reasonably concluded that CO-1080 and FD-1080-Cl, which have multiple interactions, exhibited a stronger binding ability to BSA compared to St-1080 and Et-1080. To quantitatively describe the binding ability of four molecules to BSA, the gmx_MMPBSA program was used to calculate the binding free energy (**Figure S25**). The calculated binding free energies for St-1080, Et-1080, FD-1080-Cl, and CO-1080 were -119.5, -131.7, -158.7, and -183.3 kJ/mol, respectively. The analysis suggests that the side chains of the 1080 dyes buried inside the pocket play essential roles in the formation of stable complexes. Collectively, the strong affinity can not only facilitate the dye molecule's entry into the BSA pocket, thereby reducing the water-quenching effect, but also effectively suppress the internal rotation/vibration of the molecule within the pocket.

The CO-1080@BSA probe exhibited the highest fluorescence intensity, followed by FD-1080-Cl@BSA, Et-1080@BSA, and St-1080@BSA, consistent with their respective binding energies: CO-1080 > FD-1080-Cl > Et-1080 > St-1080. However, according to the experimental results in** Figure 3**, the order of covalent binding capacity with albumin is CO-1080 > St-1080 > Et-1080 > FD-1080-Cl. The probability of nucleophilic substitution reaction is positively correlated with the distance between the two reactive functional groups. Therefore, the distances between the C atom in the Cl-C group of the dyes and the S atom in Cys476 were analyzed through molecular dynamic trajectories: 3.55 Å for CO-1080, 5.42 Å for St-1080, 6.21 Å for Et-1080, and 7.39 Å for FD-1080-Cl. These distance variations are consistent with the order of covalent binding capacity with albumin mentioned above (**Figure 4G**).

Brightness comparison of electrophoresis bands from 1:1 and 8:1 reaction ratios (**Figure S11C, D**) verified that extra dye molecule bound to Cys101 on BSA did not contribute to the overall fluorescence intensity. To understand the mechanism underlying this phenomenon, CO-1080 molecule was covalently linked to Cys101 to obtain a modified BSA protein with a non-standard amino acid residue, and a 120 ns molecular dynamics simulation was performed (**Figure S26A** and **Movie S3**). The RMSD of the CO-1080-labeled BSA protein remained in a shaking state throughout the simulation, suggesting that Cys101-bound CO-1080 was always free to move (**Figure S26B**). The superimposed final 500 frames of the trajectory (**Figure S26C**), RMSD (**Figure S26D**), and solvent-accessibility surface area (SASA) (**Figure S26E**) further verified that the Cys101-bound CO-1080 was not in a fixed position but freely moving on the surface of the protein, fully exposing to water molecules. The free movement and full contact with water molecules caused the fluorescence of the dye molecule at this binding site to be severely quenched.

Taken together, we can reasonably assume that the cyanine dye undergoes two distinct stages of interaction with BSA. In stage I, the dye molecule inserts into the calyx-shaped hydrophobic cavity of albumin through supramolecular interactions. During this process, the effectively suppressing internal rotation/vibration of the molecule results in significant brightness enhancement. In the second step, the Cl-C group of the dye and the thiol group of Cys476 in BSA preferentially form the covalent “clasp” via nucleophilic substitution, therefore immobilizing the conformation of dyes in the pocket. The supramolecular interactions in stage I catalyze their covalent attachment in stage II. The strength of the covalent binding ability is related to the distance between the thiol group of Cys476 and the Cl-C group of the dye molecule. In addition, when the reaction ratio between dye and BSA is greater than 2:1, more dyes were bound with Cys101 in stage II. We believe that this binding site did not contribute to brightness enhancement due to the unsuppressed rotation/vibration of the dye molecule.

### High-contrast NIR-II lymphography and angiography of the CO-1080@BSA probe

Considering the much-improved brightness and potential clinical use, we then performed cell toxicity experiments using free dyes and dye@BSA probes. Consistent with our expectation, the results verified that the dye@BSA complexes showed no apparent cytotoxicity compared to their free dye counterparts even at the equivalent dosage of up to 200 μΜ (**Figure S27**). Therefore, dye@BSA complexes possessed reasonable biosafety, indicating their feasibility for bioimaging in the NIR-II window. We further assessed the fundamental metabolism of 1080@BSA probes to compare their in vivo performance. Following intravenous administration of CO-1080@BSA, the probe quickly accumulated in the liver and then distributed throughout the whole body, displaying a similar hepatobiliary excretion pathway with free CO-1080. The NIR-II signals of the CO-1080@BSA-administered cohort were much higher than that of the free dye within a short timeframe (approximately 6-fold enhancement at 5 min time point). Long-term monitoring indicated that CO-1080@BSA and CO-1080 could be eventually excreted out of the body within 72 h post-injection (**Figure S28-29**). It should be noted that the overall excretion is difficult to estimate accurately due to the potential enzymatic degradation of the dye-albumin complex, as well as dissociation between the dye and albumin in vivo. Nevertheless, since albumin-derived formulations have been approved by the FDA for human use, we believe that our albumin-dye formulation also has the potential for clinical translation.

**Figure 5:**
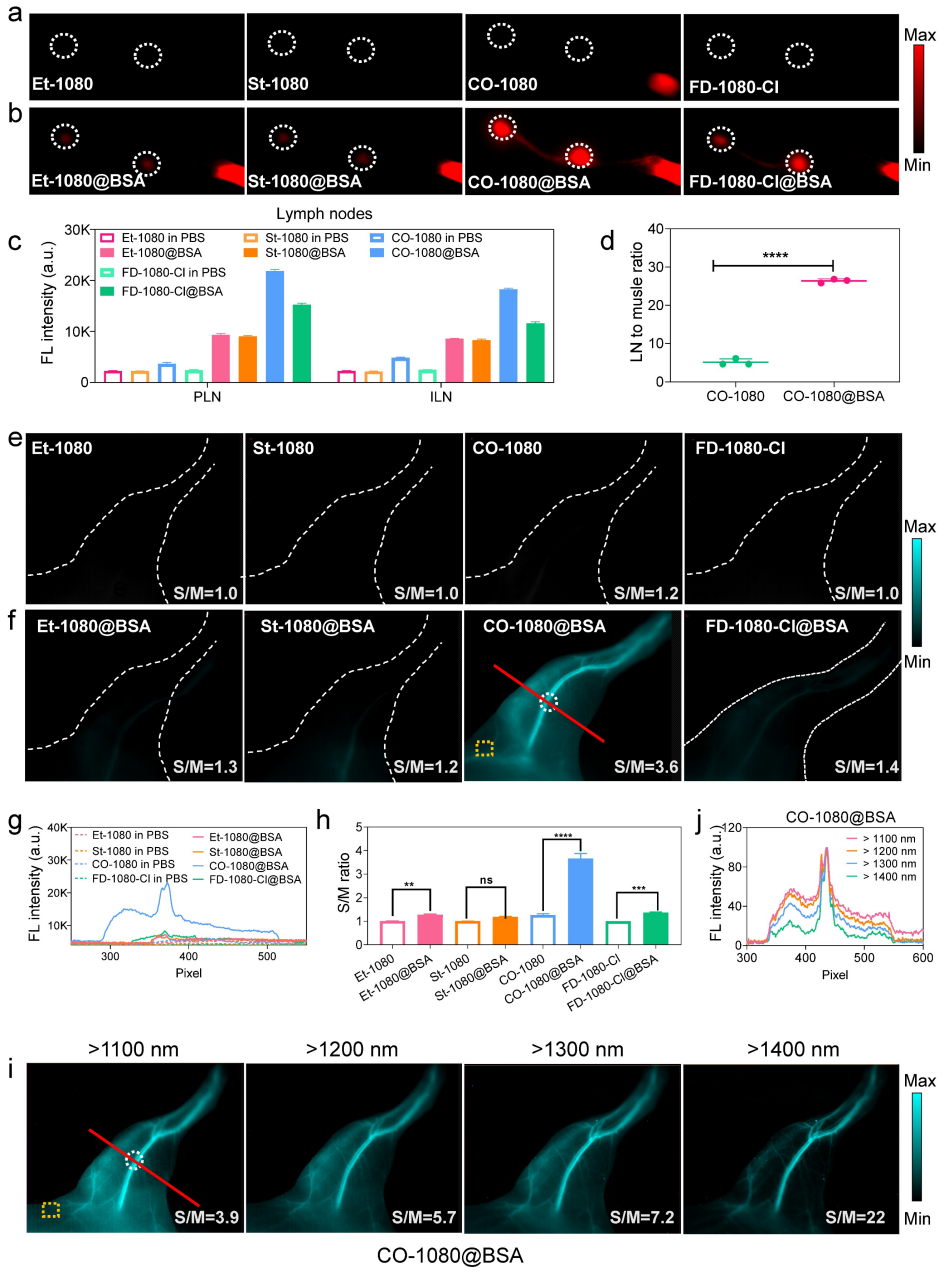
**CO-1080@BSA enables high-contrast NIR-II lymphography and angiography.** (**A**) Comparison of lymph node imaging using Et-1080, St-1080, CO-1080, and FD-1080-Cl in PBS. Imaging conditions: 980 nm excitation, > 1100 nm collection. (**B**) Comparison of lymph node imaging using Et-1080@BSA, St-1080@BSA, CO-1080@BSA, and FD-1080-Cl@BSA. (**C**) Signal quantification of popliteal and inguinal LNs after intradermal administration of 1080@BSA probes. PLN: popliteal lymph node; ILN: inguinal lymph node. (**D**) Statistical analysis of lymph node-to-skin ratios for CO-1080 and CO-1080@BSA. (**E**) Fluorescence imaging of hindlimb vasculature at the corresponding time point after Et-1080, St-1080, CO-1080, and FD-1080-Cl administration. (**F**) NIR-II imaging of hindlimb vasculature of Et-1080@BSA, St-1080@BSA, CO-1080@BSA, and FD-1080-Cl@BSA with equivalent dosage. Imaging conditions: 980 nm excitation, over 1100 nm collection. (**G**) Fluorescent cross-sectional intensity profile of the selected ROI region of hindlimb vessels. (**H**) Corresponding signal-to-muscle (S/M) ratio analysis with vessel intensity divided by fluorescence intensity of yellow ROIs. (**I**) NIR-II hindlimb vessel imaging of CO-1080@BSA was collected under various sub-NIR-II windows. The injection dosage for all NIR-II lymphatic imaging was 25 μL (200 μM). The injection dosage for all NIR-II vessel imaging was 200 μL (600 μM). (**J**) The fluorescence intensity profiles across a red line of interest in i with tunable sub-NIR-II imaging windows.

To further investigate the advantages of the CO-1080@BSA probe for NIR-II bioimaging, we compared its lymphography quality against Et-1080, St-1080, CO-1080, FD-1080-Cl, Et-1080@BSA, St-1080@BSA, and FD-1080-Cl@BSA, by injecting them into the footpads of shaved Balb/c mice at the same dosage. Under the same imaging condition, all free 1080 dyes displayed weak signals in the lymphatic system, indicating their limitation for biological application (**Figure 5A, C**). However, the CO-1080@BSA was able to illuminate the lymph node with a higher lymph node-to-muscle ratio (26) than other 1080@ probes (**Figure 5B, D and Figure S30**). ICG-based lymphography is known to be superior to lymphoscintigraphy for assessing lymphatic diseases, we thus further compared the lymphography performance of CO-1080@BSA with ICG lymphography. The results indicated that the CO-1080@BSA probe offered much higher spatial resolution for lymph node imaging compared to ICG lymphography, therefore, the sacral and popliteal LNs could be clearly distinguished after intradermal injection of CO-1080@BSA (**Figure S30**). Furthermore, CO-1080@BSA exhibited remarkably higher photostability compared with ICG under continuous laser exposure (e.g., 20 min duration). Collectively, these results indicated that CO-1080@BSA outperformed the clinically approved ICG on lymphography.

A healthy and functional vasculature plays an important role of delivering nutrients to cells and protecting organs. Reliable vascular visualization in small animals is of great significance in preclinical biomedical research. The superbright CO-1080@BSA probe with adequate biosafety, together yields real-time and noninvasive NIR-II angiography in vivo. Firstly, we performed non-invasive vascular imaging of the C57 hindlimb using both free 1080 dyes and 1080@BSA probes. Notably, under a mild imaging condition, only CO-1080@BSA was able to provide a high spatial resolution in vessel imaging, allowing for the distinction of arteries from veins (**Figure 5E-H**). The signal-to-muscle signal ratio of selected regions of interest (ROIs) demonstrated the superior imaging ability of CO-1080@BSA for hindlimb vascular visualization (**Figure 5H**). We further assessed the imaging quality/contrast of CO-1080@BSA in angiography of the hindlimb under various sub-NIR-II windows (**Figure 5I, J**). The imaging contrast of the hindlimb vessel was further improved by collecting signals at longer sub-NIR-II windows from 1100 to 1400 nm. The vessel-to-muscle ratio values in ROIs increased from 3.9 at the > 1100 nm to 22 at the > 1400 nm window. To further assess the biological toxicity of CO-1080@BSA probe in vivo, CO-1080@BSA was intravenously injected into mice. All biochemical parameters were fell within the normal range followed by performing biochemical analysis and pathological analysis of major organs after injection (**Table S3**), and no morphological abnormalities compared to the control group (PBS) were observed by H&E-staining, indicating that CO-1080@BSA possessed excellent biosafety (**Figure S31**). Overall, the CO-1080@BSA probe with impressive NIR-II imaging ability provides a complementary extension to the current NIR-II imaging in terms of brightness enhancement and pharmacokinetics improvement.

## Conclusion

The discovery of NIR-II fluorogenic dye and albumin binding functions as a brightness amplifier and pharmacokinetics regulator, thus providing us an additional perspective on NIR-II probe development. In this study, fluorogenic 1080 cyanine dyes with NIR-II peak emission as center chromophores could be packed inside protein shells to achieve enhanced brightness and satisfactory pharmacokinetics. During our manuscript submission process, we came across a report that simultaneously utilized IR1080 for in situ albumin-hitchhiking 62. Although this report shares the same structure with our CO-1080, the extra significance of our work lies in the systematic investigation of the binding mechanism between a series of 1080 dyes and albumin using mass spectrometry, shotgun proteomic analysis, and docking modeling. The screened dye (CO-1080) with optimal side structure exhibited maximum supramolecular binding affinity between albumin coupled with a stable covalent bond. Our results shed light on the interaction mechanism as well as binding sites (Cys476 and Cys101) between albumin and 1080 dyes. Our CO-1080@BSA displayed deep tissue penetration and excellent imaging resolution, which enabled noninvasive and accurate NIR-II visualization of the vascular and lymphatic systems. The imaging data of CO-1080@BSA suggests that it may be excreted through hepatobiliary clearance pathways, with minimal retention in muscles and other major organs. However, it is important to consider that dissociation between the dye and albumin could potentially lead to a decrease in brightness. In addition, the potential enzymatic degradation of the dye@albumin could also be another metabolic pathway to consider. The fluorogenic NIR-II dyes can also in-situ target albumin for deep tissue imaging (data were not included). Our methodology could enrich the library of NIR-II imaging agents to achieve next-generation NIR-II molecular bioimaging and NIR-II-guided surgery.

## Supplementary Material

Supplementary Material includes experimental methods, synthesis and characterization of NIR-II dyes, electrophoresis analysis, NIR-II fluorescence intensity, reaction optimization, quantum yield measurement, binding behavior and site characterization, in vivo biosafety characterization, and in vivo imaging.

Supplementary movie 1.

Supplementary movie 2.

Supplementary movie 3.

## Abbreviations

NIR-II: near-infrared-II
EGFR: epidermal growth factor receptor
LNs: lymph nodes
LC-HRMS: liquid chromatography high-resolution mass spectrometry
DIII: domain III
DIIIa: domain IIIa
DIa: domain Ia
DMSO: dimethylsulfoxide
RESP: restrained electrostatic potential
HOMO: highest occupied molecular orbital
LUMO: lowest unoccupied molecular orbital
BSA: bovine serum albumin
Cys: cysteine
UPLC-MS/MS: ultra-performance liquid chromatography/tandem mass spectrometry
MD: dynamics simulations
RMSD: root mean square deviation
RMSF: root mean square fluctuation
VMD: visual molecular dynamics
3D: three-dimensional
2D: two-dimensional
SASA: solvent-accessibility surface area
ICG: indocyanine green

## References

[1] Welsher K, Liu Z, Sherlock SP, Robinson JT, Chen Z, Daranciang D (2009). A Route to Brightly Fluorescent Carbon Nanotubes for Near-infrared Imaging in Mice. Nat Nanotech.

[2] Kenry Duan Y, Liu B (2018). Recent Advances of Optical Imaging in the Second Near-Infrared Window. Adv Mater.

[3] Li Y, Cai Z, Liu S, Zhang H, Wong STH, Lam JWY (2020). Design of AIEgens for near-infrared IIb imaging through structural modulation at molecular and morphological levels. Nat Commun.

[4] Lei Z, Zhang F (2021). Molecular Engineering of NIR-II Fluorophores for Improved Biomedical Detection. Angew Chem Int Ed Engl.

[5] Zhu S, Tian R, Antaris AL, Chen X, Dai H (2019). Near-Infrared-II Molecular Dyes for Cancer Imaging and Surgery. Adv Mater.

[6] Antaris AL, Chen H, Cheng K, Sun Y, Hong G, Qu C (2016). A small-molecule dye for NIR-II imaging. Nat Mater.

[7] Cosco ED, Caram JR, Bruns OT, Franke D, Day RA, Farr EP (2017). Flavylium Polymethine Fluorophores for Near- and Shortwave Infrared Imaging. Angew Chem Int Ed Engl.

[8] Wang S-F, Su B-K, Wang X-Q, Wei Y-C, Kuo K-H, Wang C-H (2022). Polyatomic molecules with emission quantum yields >20% enable efficient organic light-emitting diodes in the NIR(II) window. Nat Photonics.

[9] Liu M-H, Zhang Z, Yang Y-C, Chan Y-H (2021). Polymethine-Based Semiconducting Polymer Dots with Narrow-Band Emission and Absorption/Emission Maxima at NIR-II for Bioimaging. Angew Chem Int Ed Engl.

[10] Ren T-B, Wang Z-Y, Xiang Z, Lu P, Lai H-H, Yuan L (2021). A General Strategy for Development of Activatable NIR-II Fluorescent Probes for In Vivo High-Contrast Bioimaging. Angew Chem Int Ed Engl.

[11] Liu H, Hong G, Luo Z, Chen J, Chang J, Gong M (2019). Atomic-Precision Gold Clusters for NIR-II Imaging. Adv Mater.

[12] Fang Y, Shang J, Liu D, Shi W, Li X, Ma H (2020). Design, Synthesis, and Application of a Small Molecular NIR-II Fluorophore with Maximal Emission beyond 1200 nm. J Am Chem Soc.

[13] Li C, Chen G, Zhang Y, Wu F, Wang Q (2020). Advanced Fluorescence Imaging Technology in the Near-Infrared-II Window for Biomedical Applications. J Am Chem Soc.

[14] Fan Y, Li C, Bai S, Ma X, Yang J, Guan X (2022). NIR-II Emissive Ru(II) Metallacycle Assisting Fluorescence Imaging and Cancer Therapy. Small.

[15] Xu Y, Li C, Ma X, Tuo W, Tu L, Li X (2022). Long wavelength-emissive Ru(II) metallacycle-based photosensitizer assisting in vivo bacterial diagnosis and antibacterial treatment. Proceedings of the National Academy of Sciences.

[16] Xu Y, Li C, An J, Ma X, Yang J, Luo L (2023). Construction of a 980 nm laser-activated Pt(II) metallacycle nanosystem for efficient and safe photo-induced bacteria sterilization. Sci China Chem.

[17] Yang C, Wang H, Yokomizo S, Hickey M, Chang H, Kang H (2021). ZW800-PEG: A Renal Clearable Zwitterionic Near-Infrared Fluorophore for Potential Clinical Translation. Angew Chem Int Ed Engl.

[18] Zinn KR, Korb M, Samuel S, Warram JM, Dion D, Killingsworth C (2015). IND-Directed Safety and Biodistribution Study of Intravenously Injected Cetuximab-IRDye800 in Cynomolgus Macaques. Mol Imaging Biol.

[19] Lu G, Nishio N, van den Berg NS, Martin BA, Fakurnejad S, van Keulen S (2020). Co-administered antibody improves penetration of antibody-dye conjugate into human cancers with implications for antibody-drug conjugates. Nat Commun.

[20] Choi HS, Gibbs SL, Lee JH, Kim SH, Ashitate Y, Liu F (2013). Targeted zwitterionic near-infrared fluorophores for improved optical imaging. Nat Biotechnol.

[21] Liu R, Xu Y, Xu K, Dai Z (2021). Current trends and key considerations in the clinical translation of targeted fluorescent probes for intraoperative navigation. Aggregate.

[22] Luo X, Hu D, Gao D, Wang Y, Chen X, Liu X (2021). Metabolizable Near-Infrared-II Nanoprobes for Dynamic Imaging of Deep-Seated Tumor-Associated Macrophages in Pancreatic Cancer. ACS Nano.

[23] Zhou X, Zhang K, Yang C, Pei Y, Zhao L, Kang X (2022). Ultrabright and Highly Polarity-Sensitive NIR-I/NIR-II Fluorophores for the Tracking of Lipid Droplets and Staging of Fatty Liver Disease. Adv Funct Mater.

[24] Pan H-M, Wu C-C, Lin C-Y, Hsu C-S, Tsai Y-C, Chowdhury P (2023). Rational Design of Asymmetric Polymethines to Attain NIR(II) Bioimaging at >1100 nm. J Am Chem Soc.

[25] Bandi VG, Luciano MP, Saccomano M, Patel NL, Bischof TS, Lingg JGP (2022). Targeted multicolor in vivo imaging over 1,000 nm enabled by nonamethine cyanines. Nat Methods.

[26] Owens EA, Henary M, El Fakhri G, Choi HS (2016). Tissue-Specific Near-Infrared Fluorescence Imaging. Acc Chem Res.

[27] Kang H, Shamim M, Yin X, Adluru E, Fukuda T, Yokomizo S (2022). Tumor-Associated Immune-Cell-Mediated Tumor-Targeting Mechanism with NIR-II Fluorescence Imaging. Adv Mater.

[28] Li S, Wei J, Yao Q, Song X, Xie J, Yang H (2023). Emerging ultrasmall luminescent nanoprobes for in vivo bioimaging. Chem Soc Rev.

[29] Zhu S, Yang Q, Antaris AL, Yue J, Ma Z, Wang H (2017). Molecular imaging of biological systems with a clickable dye in the broad 800- to 1,700-nm near-infrared window. Proc Natl Acad Sci USA.

[30] Wu Y, Zhang F (2020). Exploiting molecular probes to perform near-infrared fluorescence-guided surgery. VIEW.

[31] Yang X, Shi C, Tong R, Qian W, Zhau HE, Wang R (2010). Near IR Heptamethine Cyanine Dye-Mediated Cancer Imaging. Clin Cancer Res.

[32] Tan X, Luo S, Wang D, Su Y, Cheng T, Shi C (2012). A NIR heptamethine dye with intrinsic cancer targeting, imaging and photosensitizing properties. Biomaterials.

[33] Zhang C, Liu T, Su Y, Luo S, Zhu Y, Tan X (2010). A near-infrared fluorescent heptamethine indocyanine dye with preferential tumor accumulation for in vivo imaging. Biomaterials.

[34] Thavornpradit S, Usama SM, Lin C-M, Burgess K (2019). Protein labelling and albumin binding characteristics of the near-IR Cy7 fluorophore, QuatCy. Org Biomol Chem.

[35] Thavornpradit S, Usama SM, Park GK, Shrestha JP, Nomura S, Baek Y (2019). QuatCy: A Heptamethine Cyanine Modification With Improved Characteristics. Theranostics.

[36] Usama SM, Burgess K (2021). Hows and Whys of Tumor-Seeking Dyes. Acc Chem Res.

[37] Usama SM, Lin C-M, Burgess K (2018). On the Mechanisms of Uptake of Tumor-Seeking Cyanine Dyes. Bioconjug Chem.

[38] Usama SM, Park GK, Nomura S, Baek Y, Choi HS, Burgess K (2020). Role of Albumin in Accumulation and Persistence of Tumor-Seeking Cyanine Dyes. Bioconjug Chem.

[39] Canovas C, Bellaye P-S, Moreau M, Romieu A, Denat F, Goncalves V (2018). Site-specific near-infrared fluorescent labelling of proteins on cysteine residues with meso-chloro-substituted heptamethine cyanine dyes. Org Biomol Chem.

[40] Tian R, Feng X, Wei L, Dai D, Ma Y, Pan H (2022). A genetic engineering strategy for editing near-infrared-II fluorophores. Nat Commun.

[41] Antaris AL, Chen H, Diao S, Ma Z, Zhang Z, Zhu S (2017). A high quantum yield molecule-protein complex fluorophore for near-infrared II imaging. Nat Commun.

[42] Li B, Lu L, Zhao M, Lei Z, Zhang F (2018). An Efficient 1064 nm NIR-II Excitation Fluorescent Molecular Dye for Deep-Tissue High-Resolution Dynamic Bioimaging. Angew Chem Int Ed Engl.

[43] Bai L, Hu Z, Han T, Wang Y, Xu J, Jiang G (2022). Super-stable cyanine@albumin fluorophore for enhanced NIR-II bioimaging. Theranostics.

[44] Xu J, Han T, Wang Y, Zhang F, Li M, Bai L (2022). Ultrabright Renal-Clearable Cyanine-Protein Nanoprobes for High-Quality NIR-II Angiography and Lymphography. Nano Lett.

[45] Zhu S, Hu Z, Tian R, Yung BC, Yang Q, Zhao S (2018). Repurposing Cyanine NIR-I Dyes Accelerates Clinical Translation of Near-Infrared-II (NIR-II) Bioimaging. Adv Mater.

[46] Tian R, Zeng Q, Zhu S, Lau J, Chandra S, Ertsey R (2019). Albumin-chaperoned cyanine dye yields superbright NIR-II fluorophore with enhanced pharmacokinetics. Sci Adv.

[47] Du B, Qu C, Qian K, Ren Y, Li Y, Cui X (2020). An IR820 Dye-Protein Complex for Second Near-Infrared Window and Photoacoustic Imaging. Adv Opt Mater.

[48] Feng Z, Yu X, Jiang M, Zhu L, Zhang Y, Yang W (2019). Excretable IR-820 for in vivo NIR-II fluorescence cerebrovascular imaging and photothermal therapy of subcutaneous tumor. Theranostics.

[49] Li D, Qu C, Liu Q, Wu Y, Hu X, Qian K (2020). Monitoring the Real-Time Circulatory System-Related Physiological and Pathological Processes In Vivo Using a Multifunctional NIR-II Probe. Adv Funct Mater.

[50] Zeng X, Xiao Y, Lin J, Li S, Zhou H, Nong J (2018). Near-Infrared II Dye-Protein Complex for Biomedical Imaging and Imaging-Guided Photothermal Therapy. Adv Healthc Mater.

[51] Chen Q, Wang C, Zhan Z, He W, Cheng Z, Li Y (2014). Near-infrared dye bound albumin with separated imaging and therapy wavelength channels for imaging-guided photothermal therapy. Biomaterials.

[52] He M, Wu D, Zhang Y, Fan X, Zhuang S, Yang W (2020). Protein-Enhanced NIR-IIb Emission of Indocyanine Green for Functional Bioimaging. ACS Appl Bio Mater.

[53] Tebo AG, Moeyaert B, Thauvin M, Carlon-Andres I, Böken D, Volovitch M (2021). Orthogonal fluorescent chemogenetic reporters for multicolor imaging. Nat Chem Biol.

[54] Glasgow JE, Salit ML, Cochran JR (2016). In Vivo Site-Specific Protein Tagging with Diverse Amines Using an Engineered Sortase Variant. J Am Chem Soc.

[55] Sun C, Zhao M, Zhu X, Pei P, Zhang F (2022). One-Pot Preparation of Highly Dispersed Second Near-Infrared J-Aggregate Nanoparticles Based on FD-1080 Cyanine Dye for Bioimaging and Biosensing. CCS Chem.

[56] Sun C, Li B, Zhao M, Wang S, Lei Z, Lu L (2019). J-Aggregates of Cyanine Dye for NIR-II in Vivo Dynamic Vascular Imaging beyond 1500 nm. J Am Chem Soc.

[57] Feng W, Zhang Y, Li Z, Zhai S, Lv W, Liu Z (2019). Lighting Up NIR-II Fluorescence in Vivo: An Activable Probe for Noninvasive Hydroxyl Radical Imaging. Anal Chem.

[58] Shi Y, Yuan W, Liu Q, Kong M, Li Z, Feng W (2019). Development of Polyene-Bridged Hybrid Rhodamine Fluorophores for High-Resolution NIR-II Imaging. ACS Mater Lett.

[59] Sinha SH, Owens EA, Feng Y, Yang Y, Xie Y, Tu Y (2012). Synthesis and evaluation of carbocyanine dyes as PRMT inhibitors and imaging agents. Eur J Med Chem.

[60] Lu T, Chen F (2012). Multiwfn: A multifunctional wavefunction analyzer. J Comput Chem.

[62] Xu Y, Yang C, Wu Y, Jiang W, Cheng Q, Yan L (2023). In Situ Albumin-Hitchhiking NIR-II Probes for Accurate Detection of Micrometastases. Nano Lett.

