# Supplementary material for: Site-specific albumin tagging with NIR-II fluorogenic dye for high-performance and super-stable bioimaging
Source: Theranostics. 2024 Feb 24;14(5):1860–72. doi: 10.7150/thno.88815 (PMC10945350; doi:10.7150/thno.88815)
Supplement: Supplementary file 1 — Supplementary Material includes experimental methods, synthesis and characterization of NIR-II dyes, electrophoresis analysis, NIR-II fluorescence intensity, reaction optimization, quantum yield measurement, binding behavior and site characterization, in vivo biosafety characterization, and in vivo imaging. [file thnov14p1860s1.pdf]

Supplementary Material

Site-specific albumin tagging with NIR-II fluorogenic dye for high-performance and super-stable bioimaging

Ningning Zhu, Jiajun Xu, Qi Su, Tianyang Han, Ding Zhou,* Yuewei Zhang,* Shoujun Zhu*

Table of Contents

Experimental procedures 3

Materials.. 3

Instrumentation... 3

Cyanine@protein complex 4

Gel electrophoresis analysis 4

Cell culture and cell viability 5

Animal experiments 5

NIR-II fluorescence imaging.. 5

Spectral characterization 6

Conformational search and reduced density gradient (RDG) analysis 6

Shotgun proteomics technique 7

Molecular docking analysis and molecular dynamics simulation............................. 7

Statistical analyses.. 9

Supplementary Figures 10

Supporting references 61

Experimental procedures

Materials

Benzo[*cd*]indol-2(1*H*)-one, phosphorus pentasulfide, pyridine, sodium hydroxide (NaOH), methyl iodide, acetone, methanol, 2,2-dimethyl-1,3-dioxane-4,6-dione, ethanol, triethylamine, ethyl iodide, *N,N*-dimethylformamide (DMF), potassium carbonate, concentrated hydrochloric acid (HCl), potassium iodide, acetic acid, acetic anhydride, 1-iodopropane, 1,4-butylenesulfone, potassium hydroxide, *N*-methylpyrrolidone (NMP), 2,6-di-tert-butyl-4-methyl pyridine (DTBMP), toluene, 1-butanol, and ethyl 6-bromohexanoate were purchased from Energy Chemical. Deionized water was used to prepare all the aqueous solutions. Compound **4** was synthesized according to previous literature.[1]

Tetrahydrofuran (THF), toluene (Tol), and dichloromethane (DCM) used for reactions were purified by stirring with Na and using benzophenone as the indicator, followed by evaporation.

Instrumentation

All ^{1}H-NMR spectra were obtained on Bruker AVANCE III 500 MHz or 400 MHz NMR spectrometers (Q. One Instruments Ltd.). All compounds were subjected to ^{1}H-NMR analysis to confirm sample purity. Chemical shifts were reported in ppm relative to the residual solvent peak ($CDCl_3$: ^{1}H, 7.26) or tetramethylsilane (TMS) peak. Multiplicity was indicated as follows: s (singlet), d (doublet), t (triplet), m (multiple), dd (doublet of doublets). All coupling constants (*J*) are reported in Hertz (Hz). Ultraviolet-visible-near infrared (UV-VIS-NIR) absorption spectra were recorded on PerkinElmer Lambda 950. Fluorescence emission spectra were tested on Edinburgh Instruments FL 920. Sodium dodecyl sulfate-polyacrylamide gel electrophoresis (SDS-PAGE) was performed on the American BIO-RAD electrophoresis system.

Measurement of liquid chromatography high-resolution mass spectrometry (LC-HRMS) of L-Cysteine modified 1080 series dyes

The mass spectrometry was operated under the specific conditions: ESI^+ spray voltage, 4.5 kV, or ESI-spray voltage, -3.5 kV; nebulizer gas, 1.5 L/min; drying gas,

100 kPa; heat block temperature, 200ºC; CDL temperature, 200ºC; IT Area Vacuum, 1.0×10^{-2} Pa; TOF Area Vacuum, 5×10^{-4} Pa. The ion accumulation time was set to 10 ms, and the detector voltage was fixed at 1.6 kV. The mass number calibration (ion trap and TOF analyzer) was completed using a solution of trifluoroacetic acid (TFA) and sodium hydrate. Data acquisition and analysis were performed using the LC-MS Solution version 3.0 software.

Mass spectra characterization of BSA-modified 1080 series dyes

A 20 μg protein solution was centrifuged at 12000 rcf at 4ºC for 10 min, and the supernatant was collected for later use. The samples were directly separated using the Ultimate 3000 (Thermo Fisher Scientific, USA). The mobile phase consisted of 0.1% formic acid in water (A) and 0.1% formic acid in acetonitrile (B) at a total flow rate of 0.3 mL/min. The separated samples were then detected and scanned by AB SCIEX TripleTOF 5600 mass Spectrometer. The mass range was m/z 600-4000.

Cyanine@protein complex

All dye@protein complexes were synthesized using a similar protocol and the CO-1080@BSA probe was taken here as an example. BSA was dissolved in PBS at a concentration of 10 μM. CO-1080 (IR-780, IR-1048, Et-1080, St-1080, and FD-1080-Cl) was dissolved in DMSO at 2 mM. 200 μL BSA PBS solution was first added to the Eppendorf tube. Next, 1 μL of the CO-1080 solution (IR-780, IR-1048, Et-1080, St-1080, and FD-1080-Cl) was added to the BSA solutions and quickly vortexed to mix adequately (the molar ratio of BSA and dyes was 1:1). Finally, the mixture solution was reacted under the shaker at the designed temperature for 2 hours. The dye@protein probe can be further purified with Amicon Centrifugal Filter (10-50 kDa) for five times against PBS buffer or NAP-5 Separation column.

Gel electrophoresis analysis

The complexes were loaded into 10 or 12% SDS-PAGE for electrophoresis. Then, the cyanine@protein complex solutions were mixed with protein loading buffer, followed by adding to the corresponding gel channels for electrophoresis (80 V for stacking gel and 150 V for separating gel). After electrophoresis, the binding between the protein and the dye was analyzed by detecting the NIR fluorescence signals.

Cell culture and cell viability

The mouse breast cancer cell line (4T1) was purchased from the Shanghai Enzyme Research Biotechnology Co., Ltd (Shanghai, China). Mouse fibroblasts cell line (L929) was provided by the Joint Laboratory of Opto-Functional Theranostics in Medicine and Chemistry, Jilin University. 4T1 cells and L929 cells were cultured in DMEM (containing 80 U/mL penicillin and 0.08 mg/mL streptomycin) supplemented with 10% (v/v) FBS under 5% CO_2 at 37°C in an incubator (Thermo).

The cytotoxicity of free 1080 dyes and 1080 dye@BSA probes were evaluated using the MTT assay in L929 and 4T1 cell lines. L929 cells and 4T1 cells (1×10^4 cells/well) were seeded in 96-well plates, incubated for 12 h at 37°C in a humidified incubator with 5% CO_2, and treated with mixed solutions of Et-1080, St-1080, CO-1080, FD-1080-Cl, Et-1080@BSA, St-1080@BSA, CO-1080@BSA, FD-1080-Cl@BSA, and DMEM medium at the concentrations of 0, 1, 5, 10, 30, 50, 100, and 200 μM for 24 h. The cells were then rinsed three times with PBS and incubated with 200 μL MTT (0.5 mg/mL). After removing the MTT solution after 4 hours of incubation, 200 μL DMSO was added to each well. Finally, the absorbance at 570 nm was measured by a microplate reader (Bio-Tek, Synergy LX, USA). The cytotoxicity of free dyes and dye@BSA was evaluated based on the absorption values.

Animal experiments

All animal experiments were conducted under institutional guidelines and were approved by the Animal Ethical Committee of The First Hospital of Jilin University (20210642). Balb/c mice were purchased from Liaoning Changsheng Biotechnology Co. Ltd and Beijing Vital River Laboratory Animal Technology Co., Ltd. Bedding, nesting materials, food, and water were provided ad libitum. Ambient temperature was controlled at 20 to 24°C with 12-hour light/12-hour dark cycles.

NIR-II fluorescence imaging

All mice were shaved using Nair hair removal cream (Nair™ Lotion with Aloe & Lanolin) and anesthetized with chloral hydrate or isoflurane before the experiment. All images were collected on a two-dimensional InGaAs array (Princeton Instruments, NIRvana-640) with a laser of 980 or 808 nm and a power density of 65/100 mW/cm^2.

The emission fluorescence was typically collected using different long-pass filters. A variable exposure time was used for the InGaAs camera to capture images in the sub-NIR-II windows. Meanwhile, except for blood vascular imaging using C57 mice, the rest images without special notice were all using Balb/c mice.

Spectral characterization

Absorbance spectra of free dyes and dye@BSA were acquired on an ultraviolet-visible-NIR PerkinElmer Lambda 950 spectrometer that was background corrected for each media. The NIR-II fluorescence emission spectra were captured on Edinburgh Instruments FL 920 spectroscopy by exciting with a 980 nm laser.

Conformational search and reduced density gradient (RDG) analysis

Molecular dynamics (MD) simulations of four molecules (Et-1080, St-1080, CO-1080, and FD-1080-Cl) were performed using the xTB program at the GFN0-xTB level of theory. GFN0-xTB is a computationally inexpensive method suitable for small molecule dynamics simulations. The simulation was carried out at an elevated temperature of 400 K for 100 ps to enhance conformational sampling. Solvent effects were not considered during the MD simulation, as the primary objective was potential energy surface sampling. The MD trajectory consisting of thousands of frames was subjected to batch optimization using the GFN0-xTB method via Molclus. The optimized conformations were clustered using the isostatic tool within Molclus, reducing the number of unique structures to a few hundred. The unique conformations obtained in the last step were further optimized using the GFN2-xTB method. The GFN2-xTB level of theory offers better accuracy than GFN0-xTB while maintaining a significantly lower computational cost compared to DFT methods. The optimized structures were ranked based on their energies, and the lowest-energy conformations were selected for further analysis. The selected conformations underwent geometry optimization and vibrational analysis at the B3LYP-D3(BJ)/6-31G* level of theory using Gaussian. High-level single-point energy calculations were performed using ORCA at the PWPB95-D3(BJ)/def2-QZVPP level of theory, coupled with the SMD solvation model. The resulting energies and thermal corrections from the vibrational analysis were combined to obtain the high-precision free energies in the aqueous

environment. RDG method, introduced by Weitao Yang's research group,[2] was used for visualizing and characterizing weak noncovalent interactions in the optimal structure of each molecule.

Shotgun proteomics technique

For digestion, DTT solution was added to a final concentration of 10 mmol/L, and reduced in a 56ºC water bath for 1 h. Then, IAM solution was added to a final concentration of 50 mmol/L for 40 min at room temperature in darkness. Next, the enzyme was added with an enzyme-to-substrate mass ratio of 1:100, and the first digestion was performed at 37ºC for 4 h, followed by another enzyme addition at the mass ratio of 1:100 and digested overnight. After digestion, the peptide was desalted using a self-priming desalting column, and the solvent was evaporated in a vacuum centrifuge at 45°C for the following experiments. Peptide separation was performed using a high-performance liquid chromatography system, with mobile phase A consisting of 0.1% formic acid in water and mobile B consisting of 0.1% formic acid and 80% acetonitrile. The total flow rate was 600 nL/min. The resulting peptides were detected by Q Exactive hybrid quadrupole-Orbitrap mass spectrometer. Raw MS files were analyzed and searched against the target protein database based on the species of the samples using Byonic. The parameters were set as follows: the protein modifications were carbamidomethylation (C) (variable), oxidation (M) (variable), Acetyl (Protein N-term) (variable), CO-1080 (C) (variable); the enzyme specificity was set to trypsin or chymotrypsin; the maximum missed cleavages were set to 3; the precursor ion mass tolerance was set to 20 ppm, and MS/MS mass error was 0.02 Da for fragment ions. Only highly confident identified peptides were chosen for downstream protein identification analysis.

Molecular docking analysis and molecular dynamics simulation

The AutoDockTools package was used to conduct a comprehensive study of molecular docking to explore potential interactions between four molecules (Et-1080, St-1080, CO-1080, and FD-1080-Cl) and the BSA protein (sourced from the Protein Data Bank (PDB) archive ID: 6QS9). The PDB archive provided the three-dimensional structures of BSA. The protein was treated as a rigid body for semi-

flexible molecular docking. Missing hydrogen atoms were added to the complex at pH 7.0, and the grid box dimensions were set to 18 Å (x, y, z) with a 0.375 Å grid spacing. The optimal ligand-protein complex structure, based on the scoring function, served as input files for subsequent molecular dynamics simulations. The Visual Molecular Dynamics (VMD) program was used for visualizing all structures.

Molecular dynamics simulations were performed using the GROMACS package version 2018.8 under the AMBER99SB-ILDN force field for all-atom simulation. The system was solvated with transferable intermolecular interaction potential 3 points (TIP3P) water molecules in a cubic box, maintaining a minimum distance of 10 Å between the protein and the box's edge. Extra charges were neutralized with Na^+ or Cl^- ions, and periodic boundary conditions were applied in all directions to simulate an infinite system. Steepest descents and/or conjugate gradient minimization (with a tolerance of up to 1000 kJ/mol/nm) were employed to eliminate adverse interactions, followed by a two-step equilibration process. During these stages, all atoms were restrained in position to prevent conformational changes. A constant volume (NVT) ensemble at 300 K using the V-rescale method for 1 ns, and a constant pressure (NPT) equilibration (1 ns) using the Parrinello-Rahman barostat under an isotropic pressure of 1.0 bar were employed. Production simulation was carried out for 120 ns without any restraint. Trajectories from the simulations were saved every 10 ps for analysis of root mean square deviation (RMSD), root mean square fluctuation (RMSF), and protein-ligand contact analysis. The binding energy was determined using the molecular mechanics Poisson-Boltzmann accessible surface area (MM-PBSA) method.[3]

A modified BSA protein with a non-standard amino acid residue, created by covalently linking CO-1080 to Cys101, was also subjected to molecular dynamics simulation following a similar but adjusted process. Modifications were made to acquire topology files for the non-standard residue segment. The non-standard residue segment was extracted, and the nitrogen and carbon atoms were capped with acetyl and methylamine groups, respectively. Gromacs-format topology files were generated using the Acpype tool, and a rtp file for the residue was created based on the selected

AMBER99SB-ILDN force field format. The non-standard residue's rtp file and new atom types with non-bonded parameters were added to the atomtypes.atp and ffnonbonded.itp files, respectively. The non-standard residue name was added to the GROMACS residuetypes.dat file, specifying its type as Protein. A 120 ns molecular dynamics process was executed using the GROMACS package version 2018.8, following the same procedure as described above. The trajectories were saved every 10 ps for RMSD solvent-accessible surface area (SASA) analysis.

Statistical analyses

All fluorescence intensity data are expressed as mean ± SD (n=3 for biological experiment signal acquisition and optical in vitro characterization). For normally distributed data sets with equal variances, two-way ANOVA testing followed by a Tukey post-hoc test was carried out across groups. In all cases, significance was defined as ns < 0.05, ** $p < 0.01$, *** $p < 0.001$, **** $p < 0.0001$.

Supplementary Figures

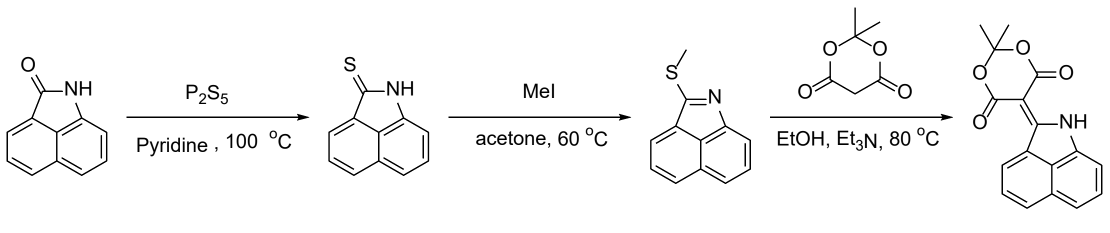

1 2 3

Figure S1. Synthetic routes of compound 3.[4]

Synthesis of benzo[*cd*]indole-2(1*H)*-thione (compound 1): Benzo[*cd*]indol-2(1*H*)-one (10 g, 59 mmol, 1.0 equiv.) and P_2S_5 (13 g, 59 mmol, 1.0 equiv.) were dissolved in pyridine (20 mL). The mixture was heated to 120°C for 8 h under N_2 atmosphere to obtain the green solution. After completion of the reaction, the solution was poured into hot water (200 mL) in batches to remove residual P_2S_5. The black precipitation was acquired through filtration, and purified through recrystallization in methanol. The reaction mixture was washed with DI water to yield a yellow-brown solid (6.6 g, 35 mmol, 60%). ^{1}H-NMR (400 MHz, $CDCl_3$, ppm) δ 8.27 (d, J = 7.1 Hz, 1H), 8.12 (d, J = 8.1 Hz, 1H), 7.76 (dd, J = 8.1, 7.2 Hz, 1H), 7.66 (d, J = 8.3 Hz, 1H), 7.49 (dd, J = 8.3 Hz, 1H), 7.12 (d, J = 7.1 Hz, 1H). LC-HRMS (ESI-TOF): calcd. for $C_{11}H_8NS^+$ [M + H]$^+$ 186.0377; found 186.0389.

Synthesis of 2-(methylthio) benzo[*cd*]indole (compound 2): Compound **1** (5 g, 27 mmol, 1.0 equiv.) and iodomethane (7.6 g, 50 mmol, 2.0 equiv.) were dissolved in acetone (30 mL) in a 100 mL single-neck flask. The resulting solution was stirred at 60°C for 9 h under nitrogen protection. The crude product was filtered, washed with acetone, and dried to yield a yellow solid (4.7 g, 24 mmol, 88%). ^{1}H-NMR (500 MHz, DMSO-d_6, ppm) δ 8.23 (d, J = 8.0 Hz, 1H), 8.14 (d, J = 7.0 Hz, 1H), 7.87 (d, J = 8.2 Hz, 1H), 7.81 – 7.77 (m, 1H), 7.71 (d, J = 7.0 Hz, 1H), 7.66 – 7.63 (m, 1H), 2.86 (s, 3H). LC-HRMS (ESI-TOF): calcd. for $C_{12}H_{10}NS^+$ [M + H]$^+$ 200.0534; found 200.0563.

Synthesis of 5-(benzo[*cd*]indol-2(1*H*)-ylidene)-2,2-dimethyl-1,3-dioxane-4,6-dione (compound 3): Compound **2** (3 g, 15 mmol, 1.0 equiv.) and 2,2-dimethyl-1,3-dioxane-4,6-dione (2 g, 15 mmol, 1.0 equiv.) were dissolved in ethanol (30 mL) with

triethylamine (610 mg, 6 mmol, 0.4 equiv.). The mixture was stirred to 80°C for 9 h under an N_2 atmosphere, cooled down to room temperature. The crude mixture was filtered, washed with ethanol and dried to yield a yellow solid (2.9 g, 9.0 mmol, 65%). ^{1}H-NMR (400 MHz, $CDCl_3$, ppm) δ 12.71 (s, 1H), 9.60 (d, J = 7.4 Hz, 1H), 8.21 (d, J = 8.0 Hz, 1H), 7.88 (t, J = 7.8 Hz, 1H), 7.78 (d, J = 8.3 Hz, 1H), 7.61 (t, J = 7.8 Hz, 1H), 7.41 (d, J = 7.2 Hz, 1H), 1.81 (s, 6H). LC-HRMS (ESI-TOF): calcd. for $C_{14}H_8NO_3$ $[M]^+$ 238.0504; found 238.0566.

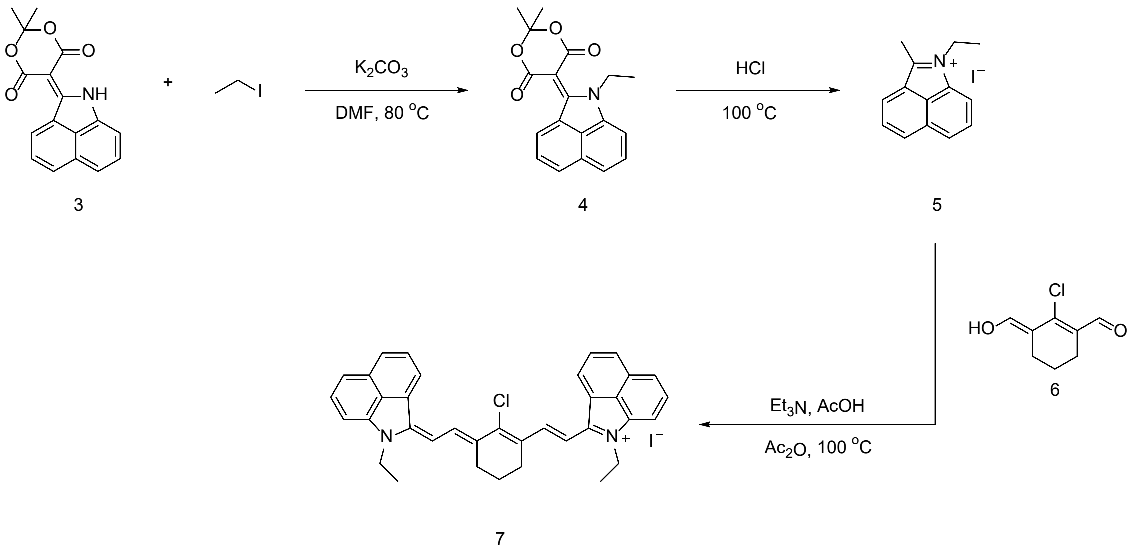

Figure S2. Synthetic routes of Et-1080 (compound 7).

Synthesis of 5-(benzo[*cd*]indol-2(1*H*)-ylidene)-2,2-dimethyl-1,3-dioxane-4,6-dione (compound 4): Compound **3** (500 mg, 1.6 mmol, 1 equiv.), potassium carbonate (701 mg, 5.1 mmol, 3 equiv.) and iodoethane (318 mg, 1.6 mmol, 1.2 equiv.) were dissolved in anhydrous DMF (5 mL). The solution was warmed to 80°C and stirred under an N_2 atmosphere for 8 h. After completion of the reaction, the reaction mixture was washed three times with 50 mL ethyl acetate and 50 mL brine, dried with anhydrous $MgSO_4$, filtered, and evaporated. The crude mixture was purified with a gradient solution (40:1 to 9:1 hexanes/ethyl acetate) through chromatograph to yield an orange solid (300 mg, 0.93 mmol, 55%). ^{1}H-NMR (400 MHz, $CDCl_3$, ppm) δ 9.01 (d, J = 7.4 Hz, 1H), 8.17 (d, J = 8.0 Hz, 1H), 7.87 – 7.78 (m, 2H), 7.62 (t, J = 7.8 Hz, 1H), 7.52 (d, J = 7.2 Hz, 1H), 4.41 (q, J = 7.2 Hz, 2H), 1.83 (s, 6H), 1.56 (t, J = 7.2

Hz, 3H). LC-HRMS (ESI-TOF): calcd. for $C_{19}H_{18}NO_4$ $[M + H]^+$ 324.1236; found 324.1280.

Synthesis of 1-ethyl-2-methylbenzo[*cd*]indol-1-ium (compound 5): Compound **4** (200 mg, 0.62 mmol, 1 equiv.) in concentrated HCl (2 mL) was heated to 100ºC and refluxed for another 1.5 h. Then the solution was cooled down to room temperature. After the addition of saturated potassium iodide solution (1M, 0.6 mL), the reaction mixture was filtered and dried to yield a red solid. (130 mg, 0.4 mmol, 75%), which was directly used in the next step without further purification. ^{1}H-NMR (400 MHz, CD_3OD, ppm) δ 8.91 (d, J = 7.2 Hz, 1H), 8.78 (d, J = 8.2 Hz, 1H), 8.47-8.44 (m, 2H), 8.19 (t, J = 7.2 Hz, 1H), 8.03 (t, J = 7.2 Hz, 1H), 4.80 (q, J = 7.4 Hz, 2H), 1.70 (t, J = 7.4 Hz, 3H). LC-HRMS (ESI-TOF): calcd. for $C_{14}H_{14}N^+$ $[M]^+$ 196.1121; found 196.1170.

Synthesis of Et-1080 (compound 7): Compound **5** (50 mg, 0.15 mmol, 1.0 equiv.), compound 6 (11.9 mg, 0.070 mmol, 0.45 equiv.) were dissolved in acetic acid (1 mL), triethylamine (0.50 mL) and acetic anhydride (0.50 mL), then heated to 60ºC for 4 h, filtered, and evaporated. The reaction mixture was vacuum filtered and triturated with ice-cold ethyl acetate to yield a dark green solid (45 mg, 0.070 mmol, 45%). ^{1}H-NMR (400 MHz, DMSO-d_6, ppm) δ 8.71 (d, J = 14.6 Hz, 2H), 8.43 (d, J = 6.9 Hz, 2H), 8.28 (d, J = 8.0 Hz, 2H), 7.97 (t, J = 7.6 Hz, 2H), 7.81 (d, J = 7.8 Hz, 2H), 7.72 – 7.62 (m, 4H), 6.90 (d, J = 13.9 Hz, 2H), 4.45 (q, J = 7.2 Hz, 4H), 2.91 (m, 4H), 1.96 (p, J = 6.4 Hz, 4H), 1.39 (t, J = 7.2 Hz, 6H). LC-HRMS (ESI-TOF): calcd. for $C_{36}H_{32}ClN_2^+$ $[M]^+$ 527.2249; found 527.2248.

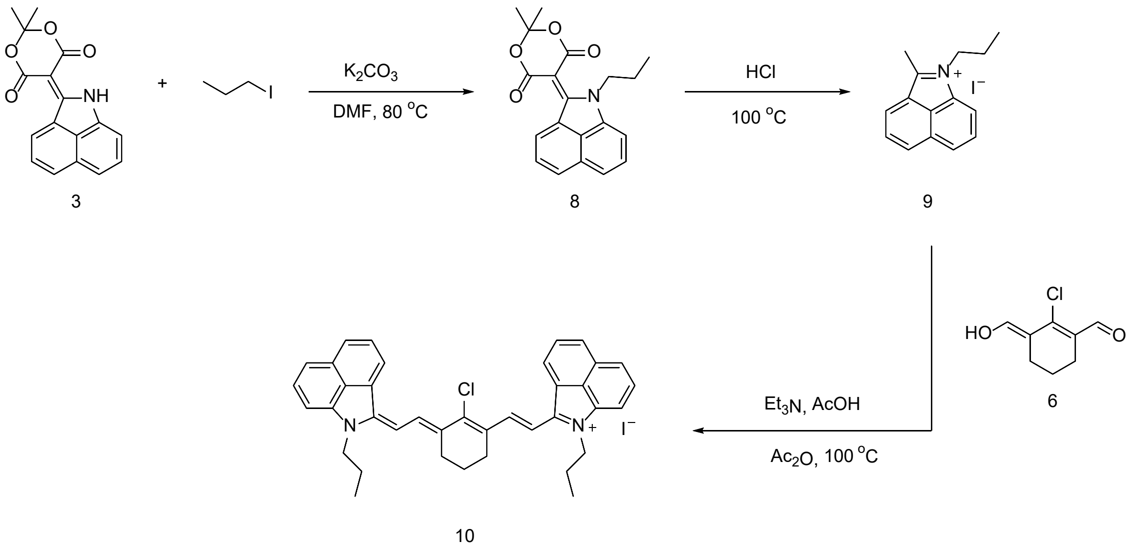

Figure S3. Synthetic routes of St-1080 (compound 10).

Synthesis of 2,2-dimethyl-5-(1-propylbenzo[cd]indol-2(1*H*)-ylidene)-1,3-dioxane-4,6-dione (compound 8): Compound **3** (500 mg, 1.6 mmol, 1 equiv.) was added to a mixture of potassium carbonate (701 mg, 5.1 mmol, 3 equiv.) and iodoethane (346 mg, 2.0 mmol, 1.2 equiv.) in anhydrous DMF (5 mL) under N_2 atmosphere. The solution was stirred at 80°C for 8 h. Then the reaction mixture was extracted with ethyl acetate (50 mL) and DI water (50 mL), dried with $MgSO_4$, filtered, and evaporated. The crude product was purified through chromatography in a gradient of hexanes with 0 to 10% ethyl acetate to yield an orange solid (320 mg, 0.94 mmol, 56%). ^{1}H-NMR (400 MHz, $CDCl_3$, ppm) δ 9.01 (d, J = 7.4 Hz, 1H), 8.16 (d, J = 8.0 Hz, 1H), 7.85 – 7.77 (m, 2H), 7.60 (t, J = 7.7 Hz, 1H), 7.49 (d, J = 7.3 Hz, 1H), 4.36 (t, J = 7.5 Hz, 2H), 1.95 (h, J = 7.2 Hz, 2H), 1.83 (s, 6H), 0.94 (t, J = 7.4 Hz, 3H). LC-HRMS (ESI-TOF): calcd. for $C_{20}H_{20}NO_4$ $[M]^+$ 338.1392; found 338.1386.

Synthesis of 2-methyl-1-propylbenzo[cd]indol-1-ium (compound 9): Compound **8** (200 mg, 0.59 mmol, 1.0 equiv.) was dissolved in concentrated HCl (2 mL) and heated to 100°C for 2 h. After completion of the reaction, the mixture was added to saturated potassium iodide solution (1 M, 0.6 mL), filtered and dried to yield a red solid. (124 mg, 0.37 mmol, 62%). ^{1}H-NMR (400 MHz, CD_3OD, ppm) δ 9.01 (d, J = 7.4 Hz, 1H), 8.16 (d, J = 8.0 Hz, 1H), 7.85 – 7.77 (m, 2H), 7.60 (t, J = 7.7 Hz, 1H),

7.49 (d, J = 7.3 Hz, 1H), 4.36 (t, J = 7.5 Hz, 2H), 1.95 (h, J = 7.2 Hz, 2H), 1.83 (s, 6H), 0.94 (t, J = 7.4 Hz, 3H). LC-HRMS (ESI-TOF): calcd. for $C_{15}H_{18}N^+$ $[M]^+$ 210.1277; found 210.1297.

Synthesis of St-1080 (compound 10): Compound **9** (50 mg, 0.15 mmol, 1.0 equiv.), compound **6** (12 mg, 0.070 mmol, 0.45 equiv.) were dissolved in acetic acid (1 mL), triethylamine (0.50 mL) and acetic anhydride (0.50 mL), then heated to 60ºC for 4 h. The reaction mixture was filtered, and washed with water and ethyl acetate to yield a dark green solid (40 mg, 0.057 mmol, 38%). ^{1}H-NMR (400 MHz, DMSO-d_6, ppm) δ 8.69 (d, J = 13.7 Hz, 2H), 8.42 (d, J = 7.5 Hz, 2H), 8.27 (d, J = 8.7 Hz, 2H), 7.96 (t, J = 7.7 Hz, 2H), 7.79 (d, J = 7.5 Hz, 2H), 7.71 – 7.61 (m, 4H), 6.89 (d, J = 14.1 Hz, 2H), 4.37 (t, J = 7.2 Hz, 4H), 2.90 (t, J = 7.2 Hz, 4H), 1.97 (p, J = 5.4 Hz, 2H), 1.89 – 1.80 (m, 4H), 0.99 (t, J = 7.3 Hz, 6H). LC-HRMS (ESI-TOF): calcd. for $C_{38}H_{36}ClN_2^+$ $[M]^+$ 555.2562; found 555.2562.

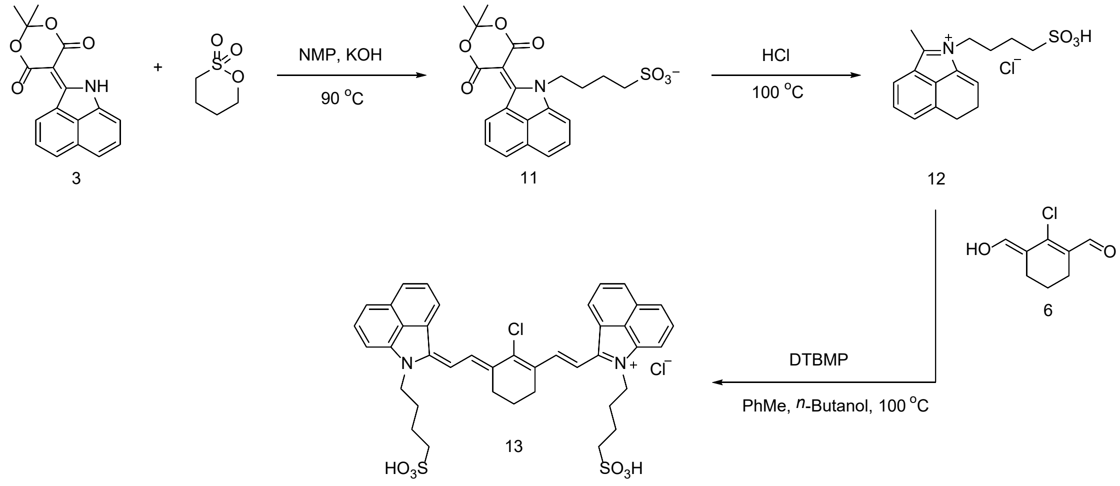

Figure S4. Synthetic routes of FD-1080 (compound 13).

Synthesis of 4-(2-(2,2-dimethyl-4,6-dioxo-1,3-dioxan-5-ylidene)-1,2-dihydrobenzo[cd]indol-1-ium-1-yl) butane-1-sulfonate (compound 11): The mixture of compound **3** (400 mg, 1.4 mmol, 1 equiv.) and KOH (152 mg, 2.7mmol, 2 equiv.) in NMP (4 mL) were stirred for 30 min at room temperature. Then 1,4-butylenesulfone (221 mg, 1.6 mmol, 1.2 equiv.) was added and further heated overnight at 90ºC. After completion of the reaction, the crude product was purified with a gradient solvent (40:1 to 5:1 dichloromethane/methanol) through

chromatograph to yield an orange solid (327 mg, 0.76 mmol, 56%). ^{1}H-NMR (400 MHz, D_2O, ppm) δ 8.99 (d, J = 7.3 Hz, 1H), 8.17 (d, J = 8.0 Hz, 1H), 7.86 – 7.78 (m, 2H), 7.61 (t, J = 7.8 Hz, 1H), 7.49 (d, J = 7.3 Hz, 1H), 4.37 (t, J = 7.6 Hz, 2H), 4.09 (q, J = 7.2 Hz, 2H), 2.26 (t, J = 7.4 Hz, 2H), 1.96 (p, J = 7.4 Hz, 2H), 1.83 (s, 6H), 1.64 (p, J = 7.4 Hz, 2H), 1.38(p, J = 7.4 Hz, 2H), 1.22 (t, J = 7.2 Hz, 3H). LC-HRMS (ESI-TOF): calcd. for $C_{21}H_{21}NO_7S$ [M + 2 H]$^+$ 432.1117; found 432.1140.

Synthesis of 4-(2-methyl-6,7-dihydrobenzo[*cd*]indol-1-ium-1-yl) butane-1-sulfonate (compound 12): Compound **11** (150 mg, 0.19 mmol) was dissolved in concentrated HCl (2 mL) and stirred at 100ºC for 2 h. After completion of the reaction, the solvent was removed by using a rotary evaporator and then crystallized with acetone and acetonitrile to yield a green solid (110 mg, 0.133 mmol, 82%). ^{1}H-NMR (400 MHz, D_2O, ppm) δ 8.55 (d, J = 7.2 Hz, 1H), 8.46 (d, J = 8.1 Hz, 1H), 8.17-8.12 (m, 2H), 7.90 (t, J = 7.7 Hz, 1H), 7.79 (t, J = 7.8 Hz, 1H), 4.59 (t, J = 7.6 Hz, 2H), 2.97 (t, J = 7.6 Hz, 2H), 2.12 (p, J = 7.9 Hz, 2H), 1.90 (p, J = 7.9 Hz, 2H). LC-HRMS (ESI-TOF): calcd. for $C_{16}H_{18}NO_3S^+$ [M]$^+$ 304.1002; found 304.1035.

Synthesis of FD-1080-Cl (compound 13): Compound **12** (60 mg, 0.20 mmol, 1.0 equiv.), compound **6** (13 mg, 0.079 mmol, 0.40 equiv.) and 2,6-di-tert-butyl-4-methyl pyridine (120 mg, 0.59 mmol, 3.0 equiv.) were dissolved in anhydrous *n*-butanol (1.4 mL) and toluene (0.6 mL). The solution was heated to 100ºC under N_2 protection for 12 h. After completion of the reaction, the crude product was filtered, purified by trituration in a mixture of acetate and methanol, and dried to yield a dark green solid. LC-HRMS (ESI-TOF): calcd. for $C_{40}H_{40}ClN_2O_6S_2^+$ [M]$^+$ 743.2011; found 743.2002.

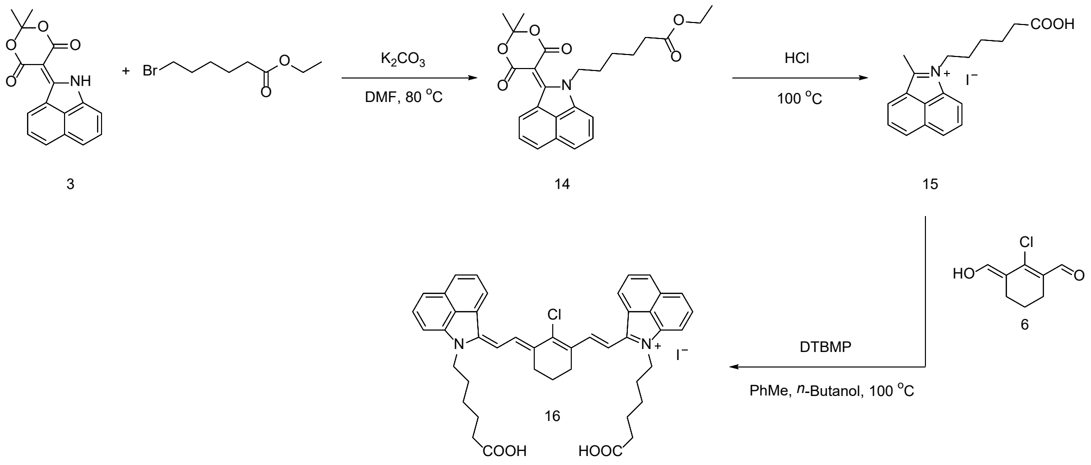

Figure S5. Synthetic routes of CO-1080 (compound 16).

Synthesis of ethyl 6-(2-(2,2-dimethyl-4,6-dioxo-1,3-dioxan-5-ylidene) benzo[cd]indol-1(2*H*)-yl) hexanoate (compound 14): Compound **3** (400 mg, 1.4 mmol, 1 equiv.), ethyl 6-bromohexanoate (363 mg, 1.6 mmol, 1.2 equiv.), and K_2CO_3 (562 mg, 4.1 mmol, 3 equiv.) were dissolved into anhydrous DMF (5 mL). The reaction was stirred overnight at 80°C with nitrogen protection. After the addition of saturated salt water, the reaction mixture was extracted with ethyl acetate, dried with Na_2SO_4, filtered, and evaporated. The crude product was purified through chromatography in a gradient of dichloromethane with 0 to 10 % methanol to yield an orange solid (332 mg, 0.78 mmol, 56%).^{1}H-NMR (400 MHz, $CDCl_3$, ppm) δ 8.99 (d, J = 7.3 Hz, 1H), 8.17 (d, J = 8.0 Hz, 1H), 7.86 – 7.78 (m, 2H), 7.61 (t, J = 7.8 Hz, 1H), 7.49 (d, J = 7.3 Hz, 1H), 4.37 (t, J = 7.6 Hz, 2H), 4.09 (q, J = 7.2 Hz, 2H), 2.26 (t, J = 7.4 Hz, 2H), 1.96 (p, J = 7.4 Hz, 2H), 1.83 (s, 6H), 1.64 (p, J = 7.4 Hz, 2H), 1.38(p, J = 7.4 Hz, 2H), 1.22 (t, J = 7.2 Hz, 3H). LC-HRMS (ESI-TOF): calcd. For $C_{21}H_{18}NO_6$ $[M]^+$ 380.1134; found 380.1462.

Synthesis of 1-(5-carboxypentyl)-2-methylbenzo[*cd*]indol-1-ium (compound 15): Compound **14** (200 mg, 0.46 mmol, 1 equiv.) in concentrated HCl (2 mL) was heated to 100°C for 2 h and then the solution was cooled down to room temperature. Finally, KI solution (1 M, 0.5 mL) was added to obtain a red precipitate. The crude product was filtered, washed with water and ethyl acetate, and dried (135 mg, 0.33 mmol, 72%). ^{1}H-NMR (400 MHz, CD_3OD, ppm) δ 8.88 (d, J = 7.2 Hz, 1H), 8.75 (d, J = 8.0 Hz, 1H) , 8.45 – 8.39 (m, 2H) , 8.21 – 8.13 (m, 1H), 8.04 – 7.97 (m, 1H), 4.72 (t, J =

7.7 Hz, 2H), 2.34 (t, J = 7.2 Hz, 2H), 2.09 (p, J = 7.7 Hz, 2H), 1.72 (p, J = 7.2 Hz, 1H) , 1.64 – 1.53 (m, 2H). LC-HRMS (ESI-TOF): calcd. For $C_{18}H_{20}NO_2^+$ $[M]^+$ 282.1489, found 282.1455.

Synthesis of CO-1080 (compound 16): Compound **15** (50 mg, 0.12 mmol, 1.0 equiv.), compound **6** (9.4 mg, 0.055 mmol, 0.45 equiv.) and 2,6-di-tert-butyl-4-methyl pyridine (75 mg, 0.37 mmol, 3.0 equiv.) were dissolved in anhydrous *n*-butanol (1.4 mL) and toluene (0.60 mL), then heated to 100°C for 12 h. After completion of the reaction, the reaction mixture was filtered and purified by trituration in ethyl acetate to yield a dark green solid (45 mg, 0.055 mmol, 45%). ^{1}H-NMR (400 MHz, DMSO, ppm) δ 8.54 (d, J = 14.0 Hz, 2H), 8.25 (d, J = 7.8 Hz, 2H), 8.14 (d, J = 8.1 Hz, 2H), 7.88 (t, J = 7.7 Hz, 2H), 7.63 (d, J = 8.1 Hz, 2H), 7.54 (t, J = 7.7 Hz, 2H), 7.48 (d, J = 7.3 Hz, 2H), 6.70 (d, J = 14.1 Hz, 2H), 4.24 (t, J = 7.8 Hz, 4H), 2.83 (t, J = 6.4 Hz, 4H), 2.21 (t, J = 7.3 Hz, 4H), 1.96-1.93 (m, 2H), 1.76 (p, J = 7.3 Hz, 4H), 1.57 (p, J = 7.4 Hz, 4H), 1.40 (p, J = 7.6 Hz, 4H). LC-HRMS (ESI-TOF): calcd. For $C_{44}H_{44}ClN_2O_4^+$ $[M]^+$ 699.2984; found 699.2980.

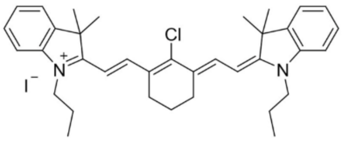

IR-780

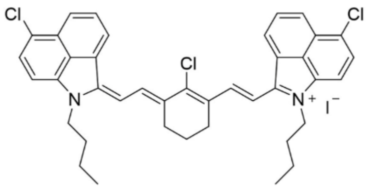

IR-1048

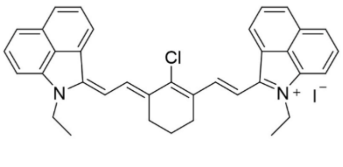

Et-1080

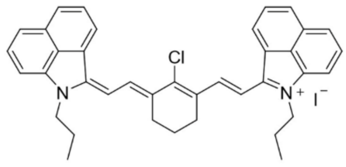

St-1080

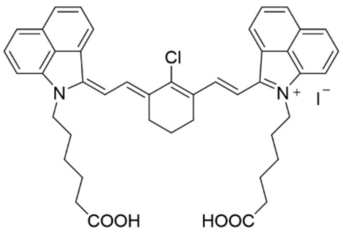

CO-1080

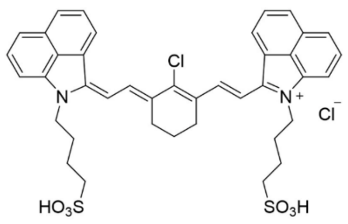

FD-1080-Cl

Figure S6. Chemical structures of commercial IR-780, IR-1048, and our 1080 series dyes with NIR-II peak emission.

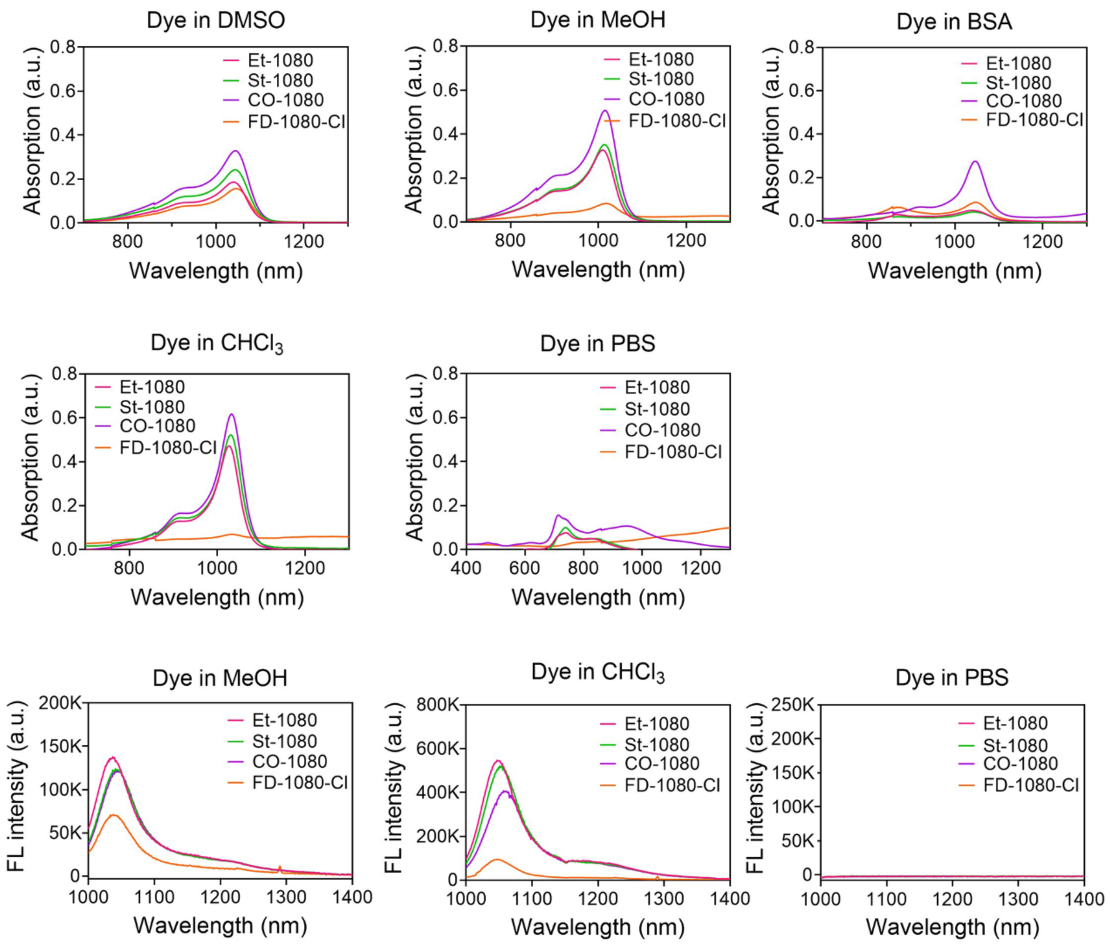

Figure S7. Absorption and fluorescence emission of 1080 dyes in different solvents. Absorption and emission spectra of 1080 dyes (10 μM) in five solutions (DMSO, MeOH, $CHCl_3$, BSA (10 μM), PBS) under 980 nm laser irradiation.

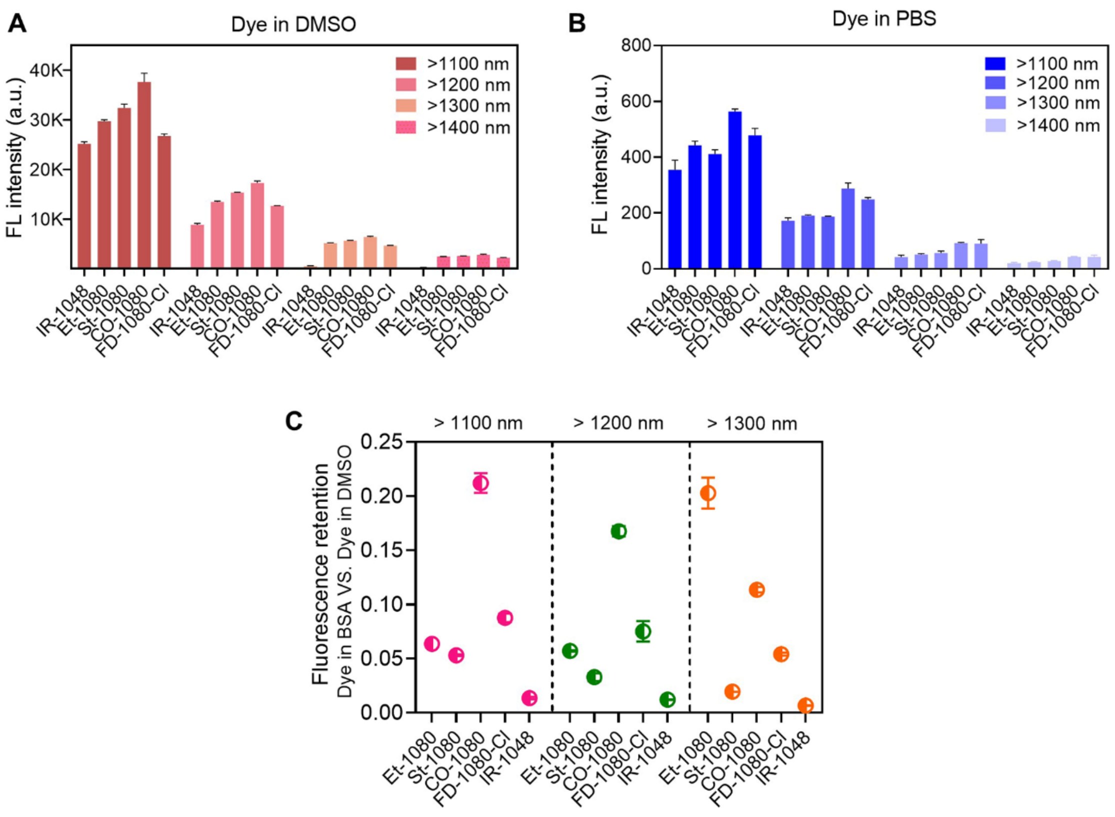

Figure S8. Brightness of 1080 dyes in DMSO/PBS and 1080@BSA under different sub-NIR-II windows. (A, B) Fluorescence intensity of different cyanine dyes in (**A**) DMSO and (**B**) PBS solution (10 µM, > 1100 nm) under different wavelengths at the same exposure time. (**C**) Fluorescence retention of cyanine dyes@BSA compared with their free dye counterparts in DMSO (10 µM, Ex: 980 nm, > 1100 nm collection).

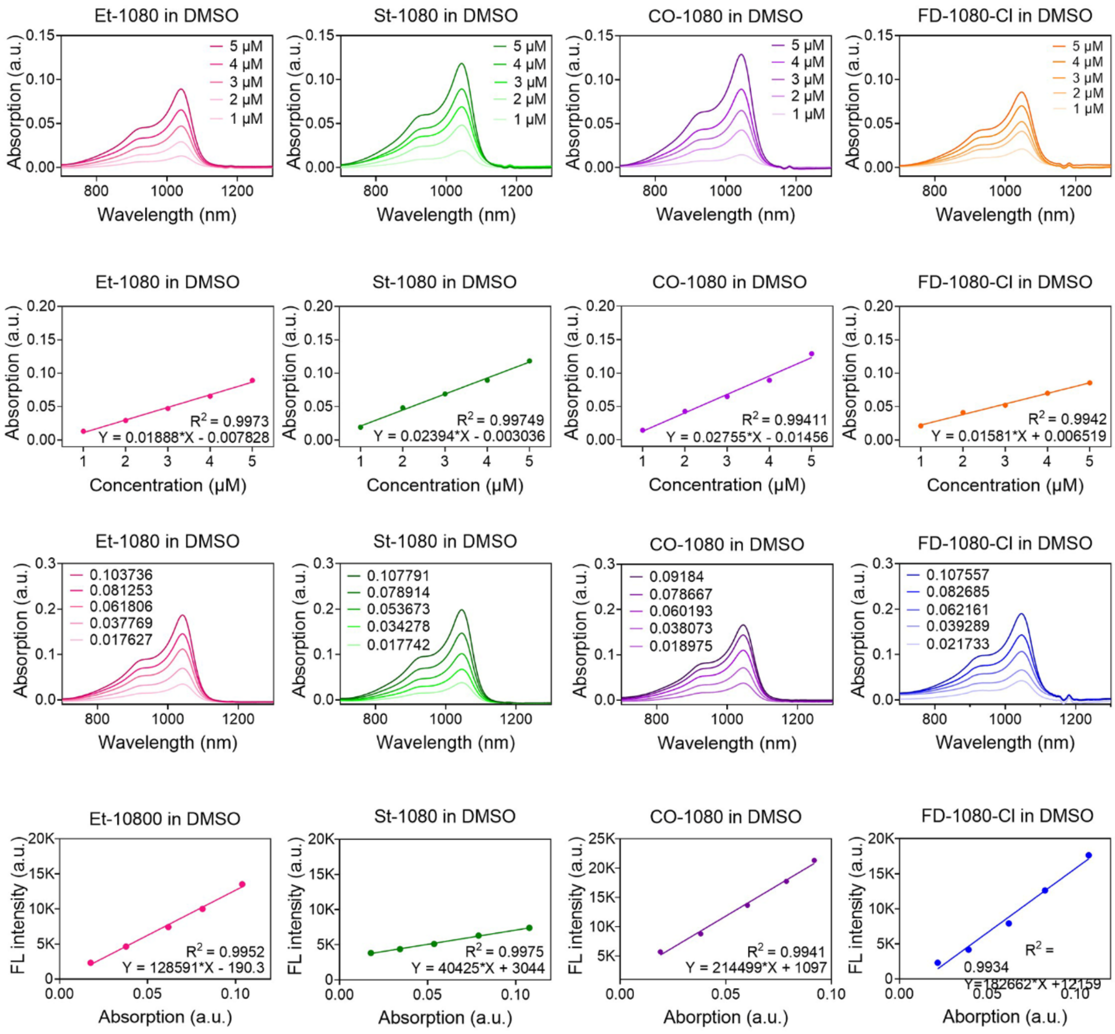

Figure S9. Molar absorption coefficients and NIR-II quantum yields of dyes in DMSO.

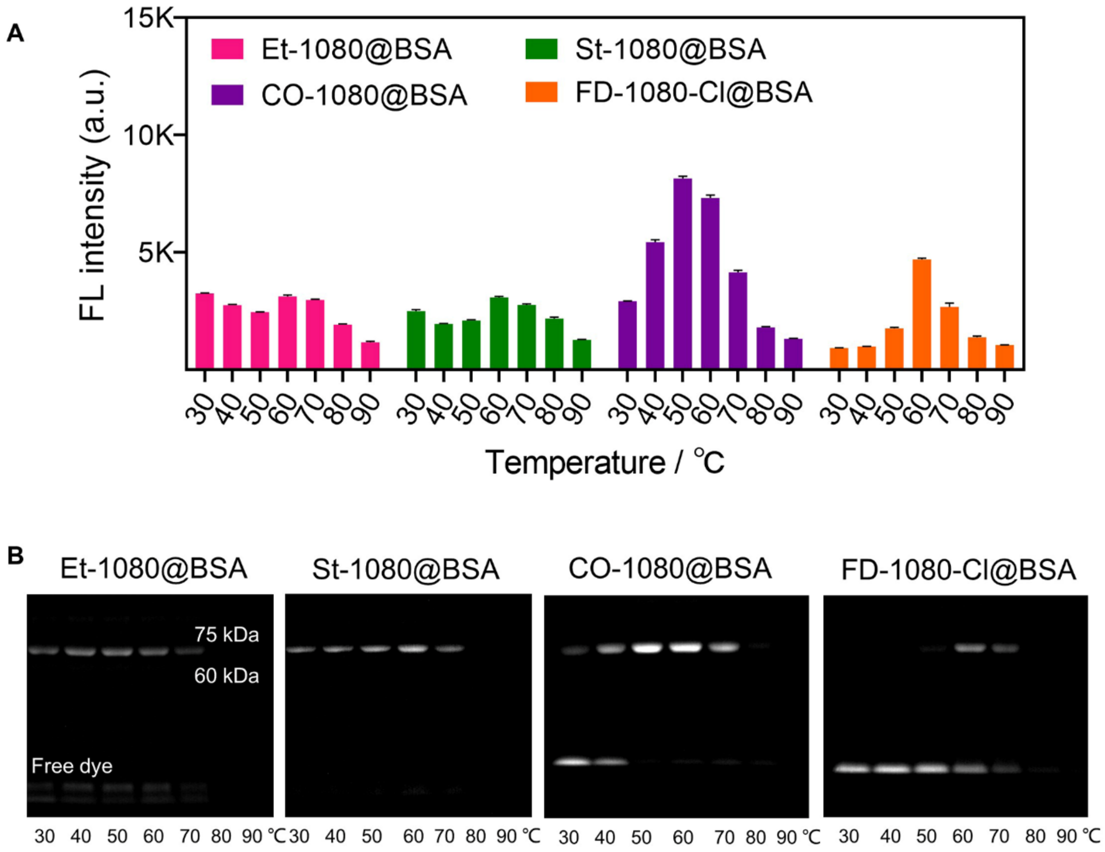

Figure S10. Optimization of reaction temperature for 1080@BSA. (**A**) Fluorescence intensity of different 1080 dyes in BSA under different reaction temperatures (10 μM, > 1100 nm collection, Ex: 980 nm). (**B**) Electrophoresis analysis of different 1080 dyes in BSA at different reaction temperatures.

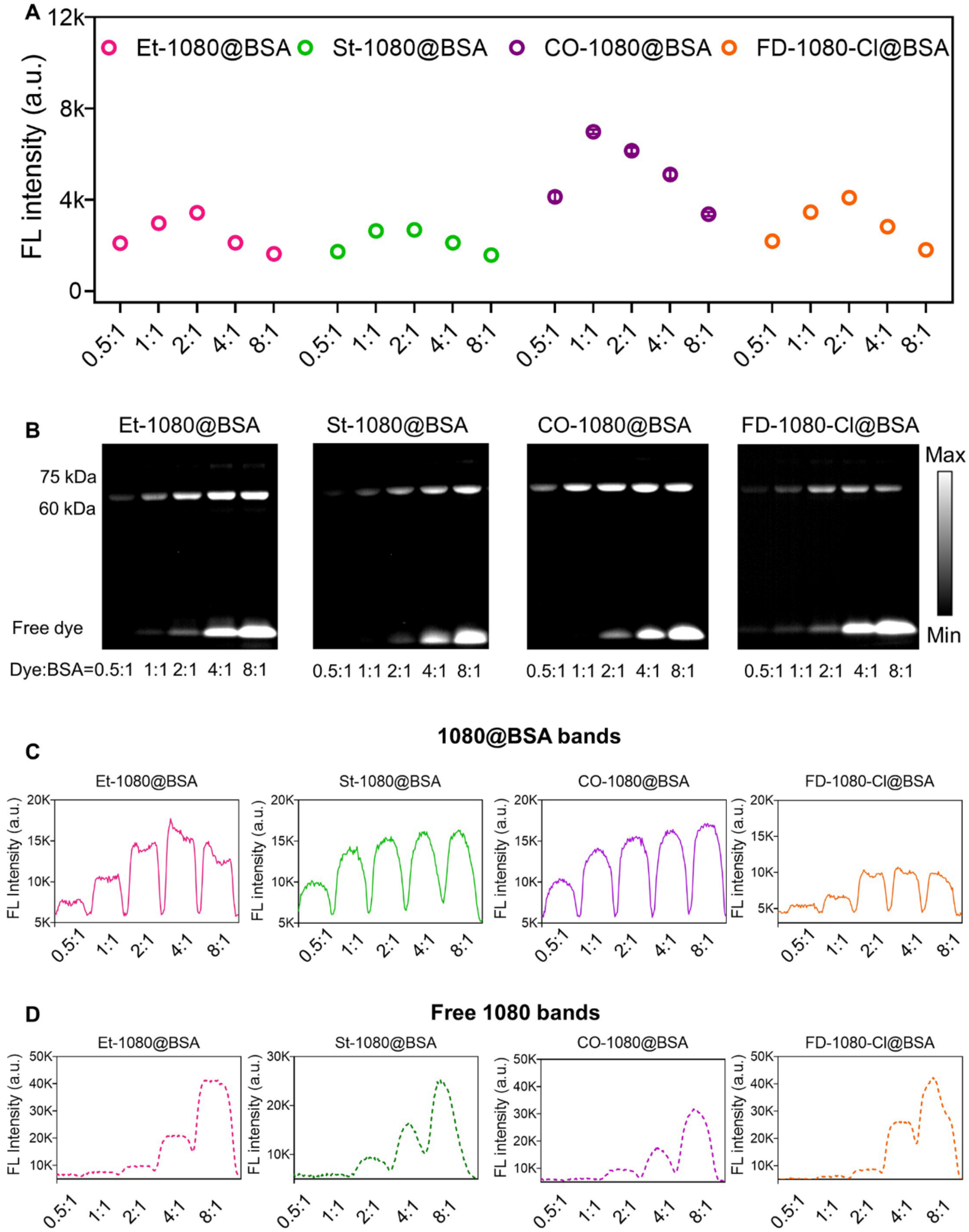

Figure S11. Optimization of reaction ratio for 1080@BSA. (**A**) Fluorescence intensities of different 1080 dyes in BSA under different reaction ratios (10 μM, > 1100 nm collection, Ex: 980 nm). (**B**) Electrophoresis analysis of different 1080 dyes in BSA under different reaction ratios. (**C, D**) Fluorescent cross-sectional intensity profile of **C**) 1080@BSA bands and **D**) free 1080 bands from electrophoresis images.

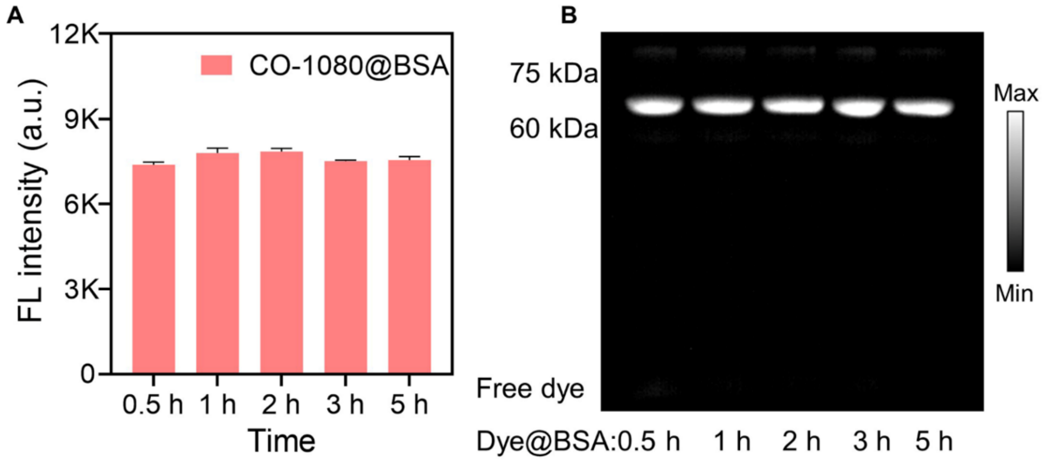

Figure S12. Optimization of reaction time for CO-1080@BSA. (**A**) Fluorescence intensity of CO-1080 in BSA under different reaction time (10 μM, > 1100 nm, Ex: 980 nm). (**B**) Electrophoresis analysis of CO-1080 in BSA under different reaction time.

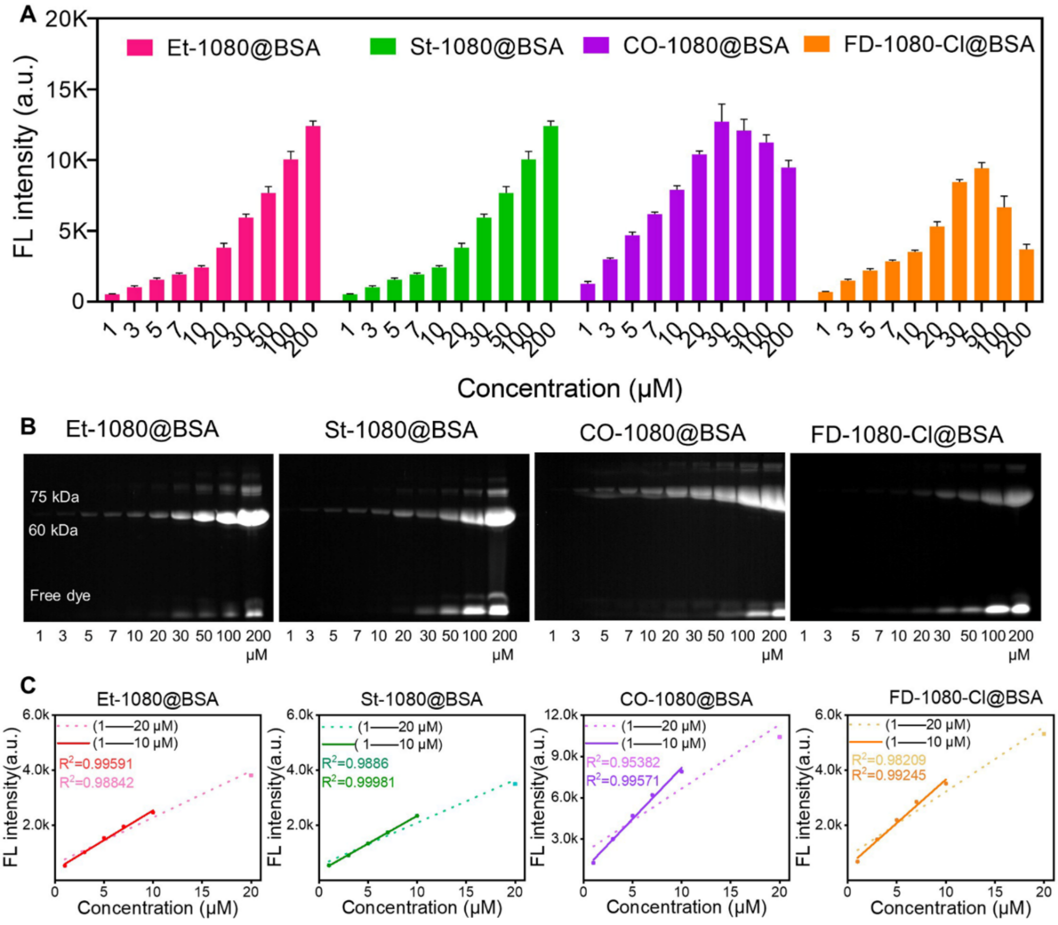

Figure S13. Optimization of reaction concentration for 1080@BSA. (**A**) Fluorescence intensities of different 1080 dyes in BSA under different reaction concentrations (10 μM, > 1100 nm, Ex: 980 nm). (**B**) Electrophoresis analysis of different cyanine dyes in BSA under different reaction concentrations. (**C**) Linear fitting of four 1080 dyes in BSA brightness against reaction concentrations.

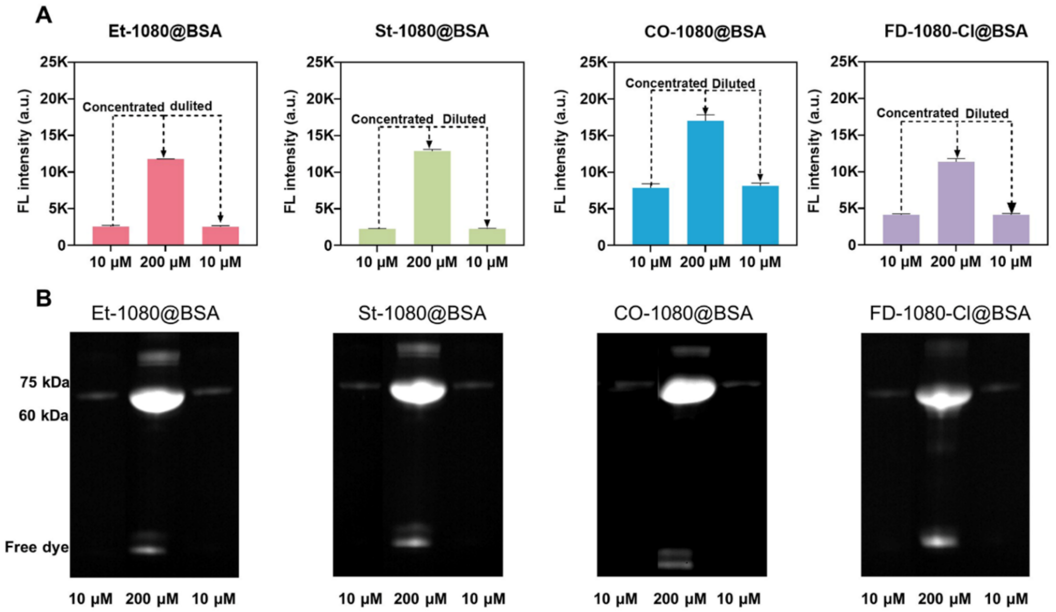

Figure S14. Concentration/dilution of 1080@BSA kept the optimal reaction concentration with maximum brightness, and simultaneously satisfied the high dosage application with high concentration. (**A**) Brightness comparison of 1080 dyes in BSA before/after concentration followed by further dilution. (**B**) Electrophoresis analysis of four 1080 dyes in BSA before/after concentration and further dilution.

Note for Figure S10-S14:

The confirmed optimal condition indicated that: (1) The reaction temperature of 50ºC enabled sufficient fluorescence enhancement for CO-1080@BSA complex, whereas the brightness of the remained dye@BSA complexes increased significantly at 60ºC (**Figure S10**). (2) When the ratio of dyes to BSA exceeds 1:1, significant free dye bands were observed (**Figure S11**). (3) The fluorescence intensity of the CO-1080@BSA complex reached a maximum at 2 hours (**Figure S12**). To determine the optimal concentration of reaction, we fitted the brightness of four cyanine dye complexes against reaction concentration, which demonstrated that a linear relationship was only observed at reaction concentrations less than 10 µM. This inspired us to further optimize the reaction concentration under 10 µM for the maximally bright probe (**Figure S13**). For the subsequent experiments, we manufactured a series of complexes at the optimal reaction concentration of 10 µM and concentrated them by ultrafiltration to effectively prevent the intramolecular fluorescence quenching under high reaction concentration. Additionally, the fluorescence intensity of Et-1080@BSA, St-1080@BSA, CO-1080@BSA, and FD-1080-Cl@BSA would basically recover after diluting the concentrated sample into its original concentration (**Figure S14**).

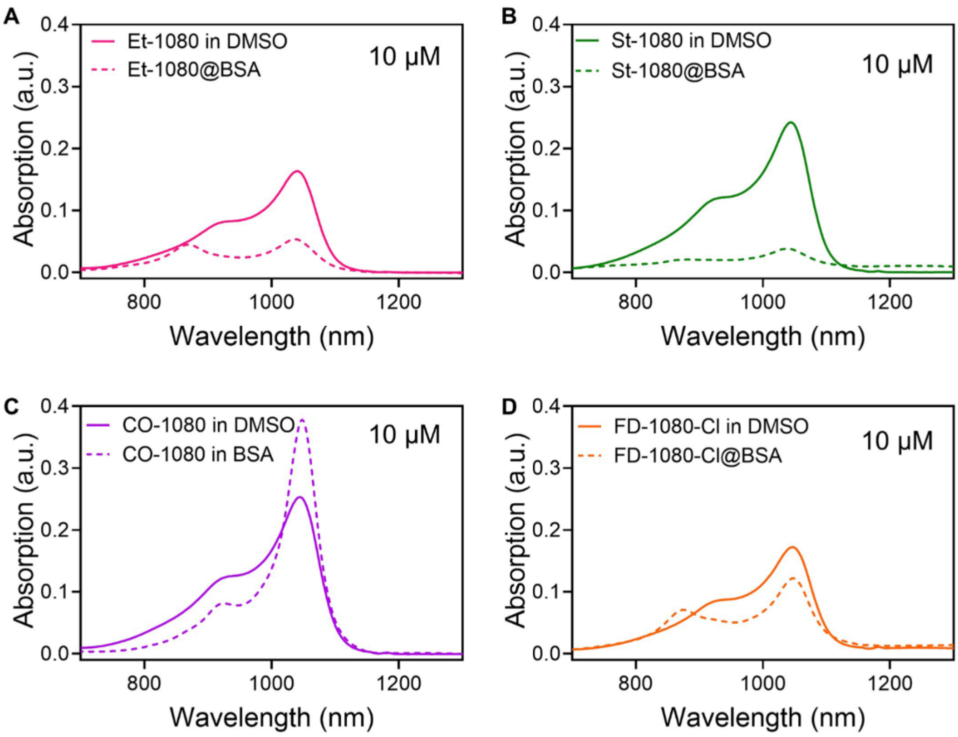

Figure S15. Comparison of absorption between 1080 dyes in DMSO and 1080@BSA probes at equal concentration. (**A**) Et-1080 and Et-1080@BSA, (**B**) St-1080 and St-1080@BSA, (**C**) CO-1080 and CO-1080@BSA, (**D**) FD-1080-Cl and FD-1080-Cl@BSA.

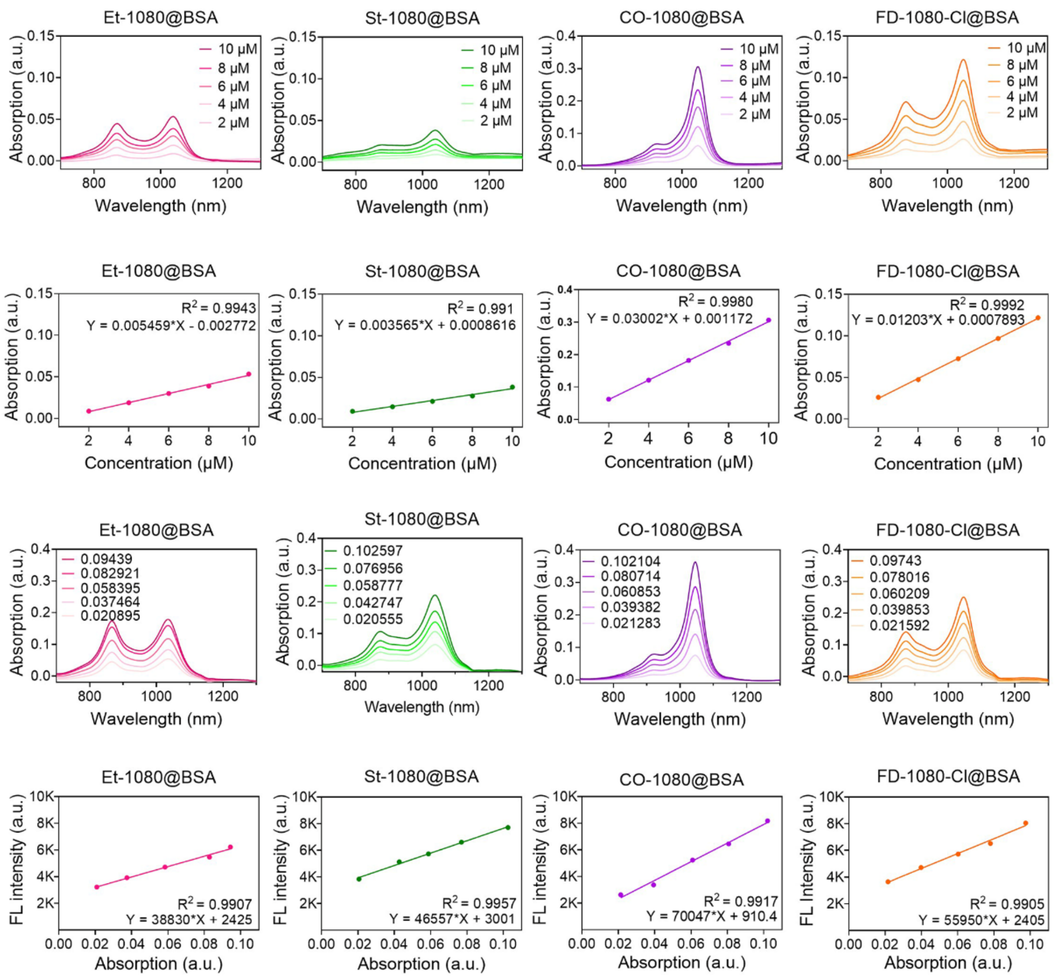

Figure S16. Molar absorption coefficients and NIR-II quantum yields of 1080@BSA.

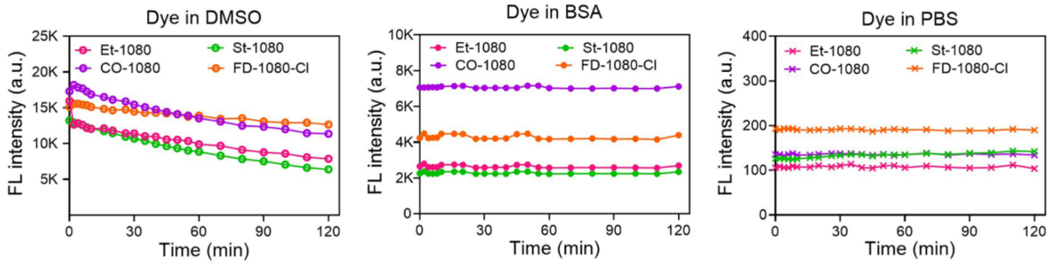

Figure S17. Photostability of 1080 dyes and 1080@BSA. NIR-II fluorescence intensity of four 1080 dyes in DMSO, PBS, and BSA solution (10 μM, Ex: 980 nm, > 1100 nm) under continuous laser irradiation.

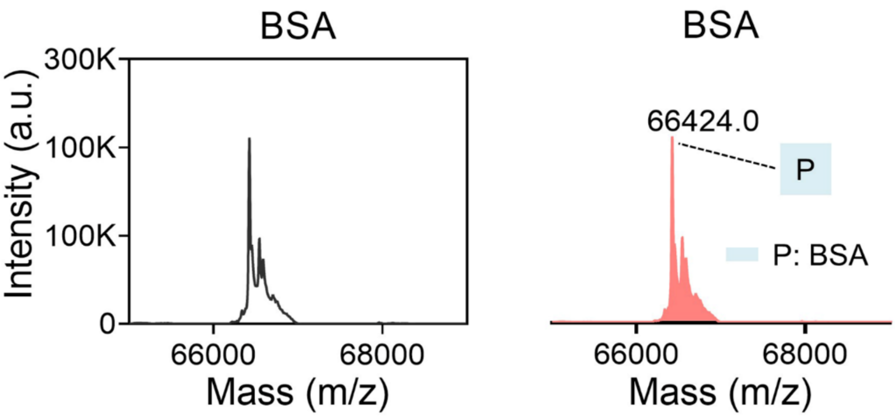

Figure S18. High-resolution mass spectrometry characterization of BSA.

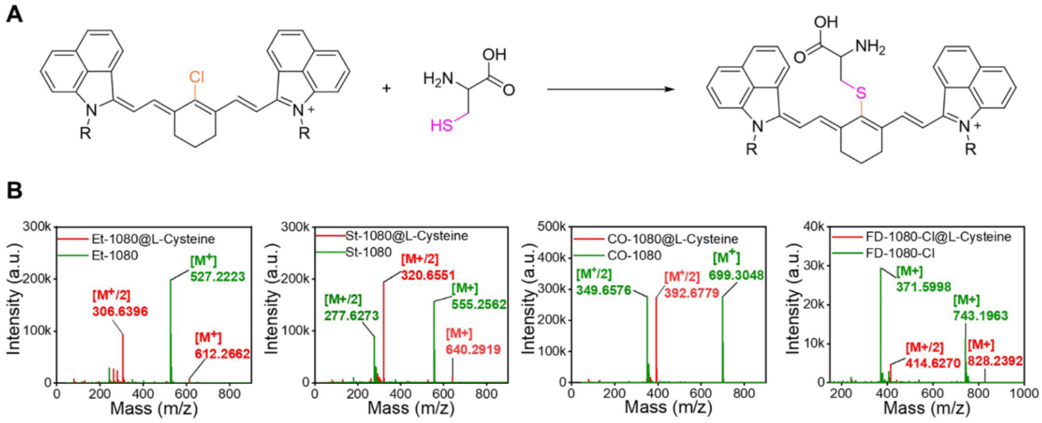

Figure S19. Validation of covalent formation via displacement of chloride of 1080 dyes by thiol group on cysteine. (**A**) Schematic diagram of the binding between L-cysteine and 1080 dyes (heavily reliant on the base catalyst). (**B**) High-resolution mass spectrometry characterization of a covalent bond between the L-cysteine and 1080 series dyes.

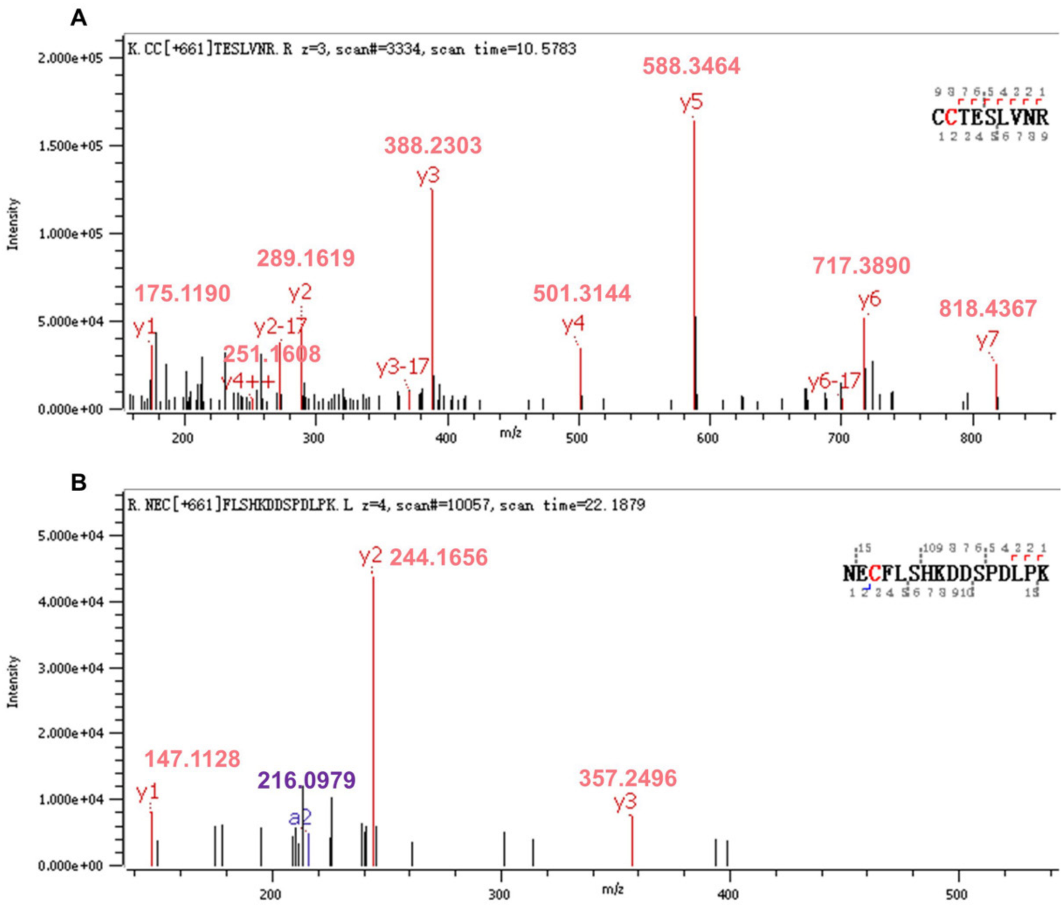

Figure S20. Shotgun proteomics analysis of CO-1080-labeled BSA. LC-MS/MS spectra revealed that (**A**) Cys476 and (**B**) Cys101 contributed to the formation of the covalent bonds.

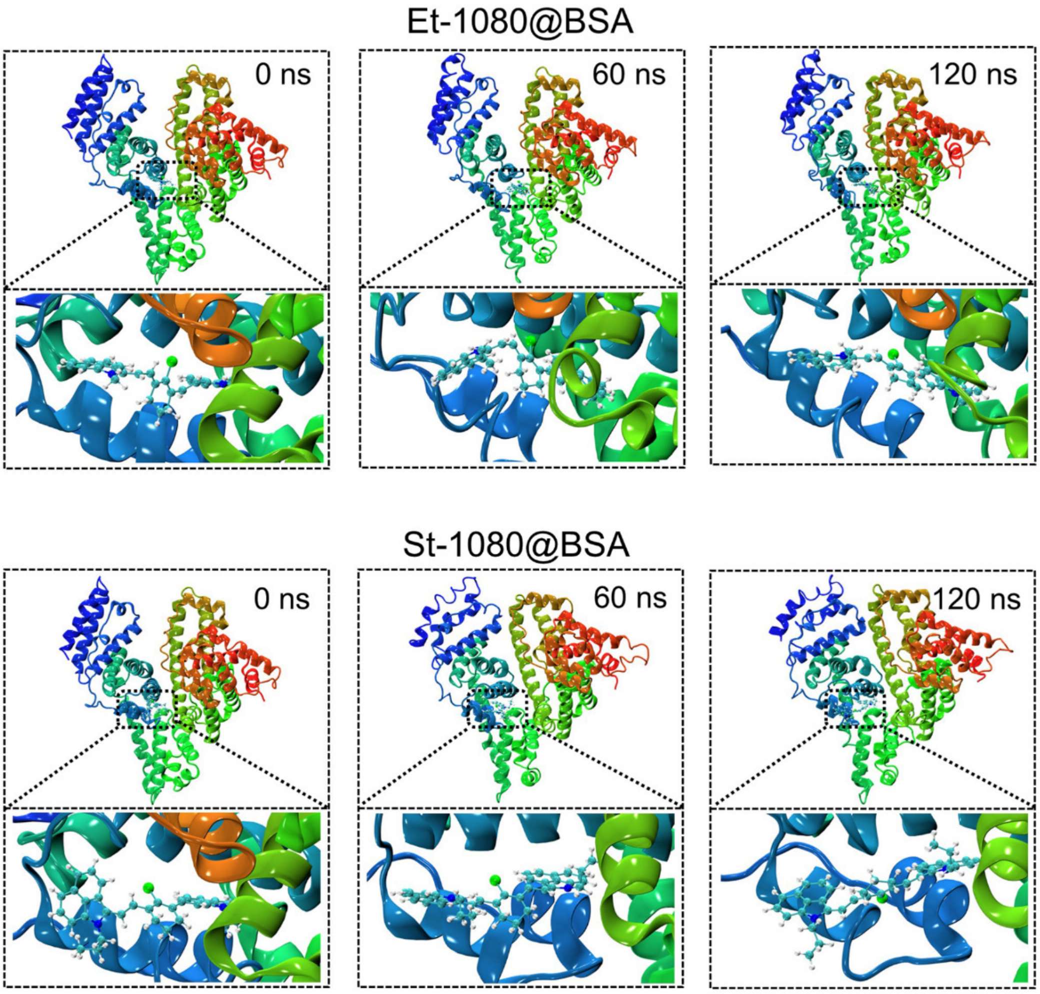

Figure S21. Structures of Et-1080 and St-1080 binding to BSA, which are extracted from the molecular dynamics simulation trajectory at 0, 100, and 200 ns, respectively.

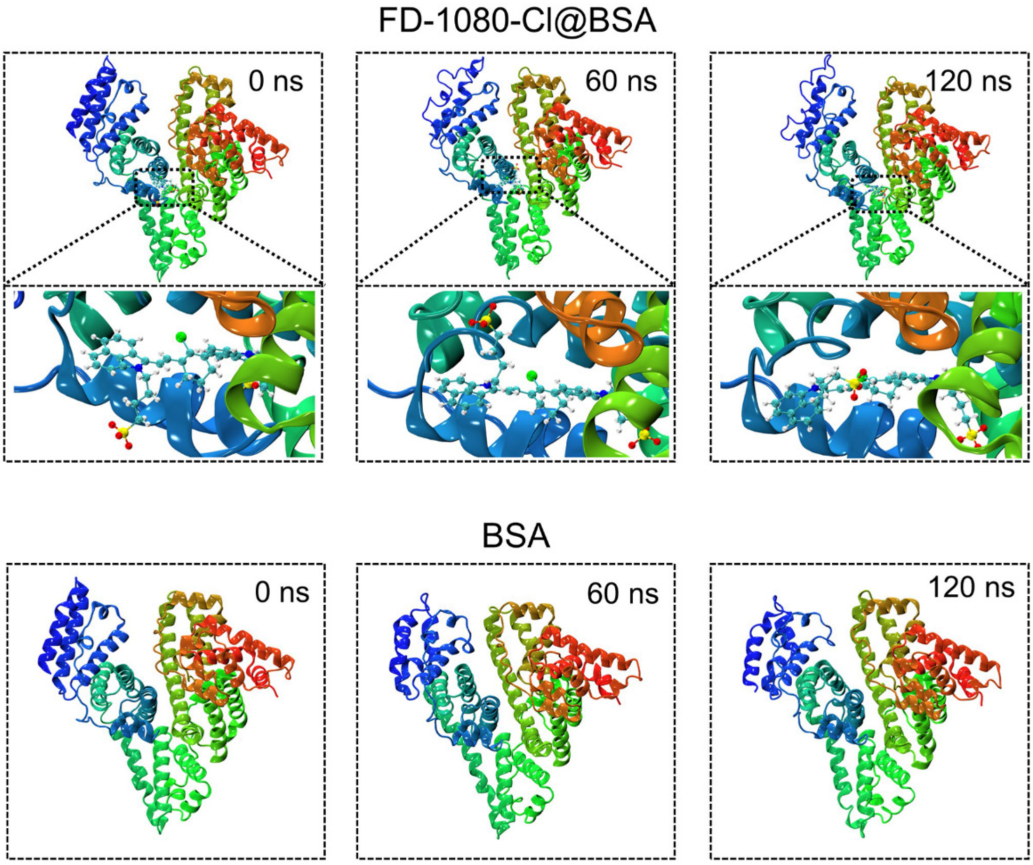

Figure S22. Structures of FD-1080-Cl binding to BSA and free BSA, which are extracted from the molecular dynamics simulation trajectory at 0, 100, and 200 ns, respectively.

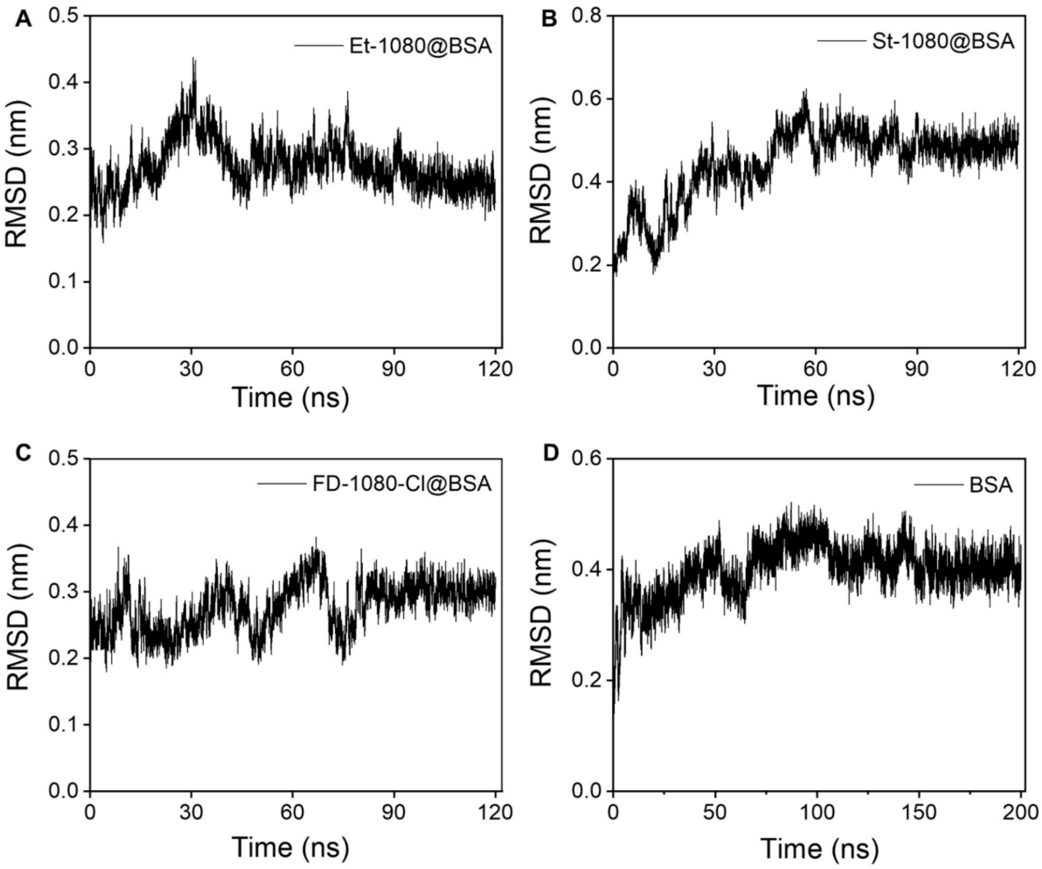

Figure S23. RMSD of (**A**) Et-1080@BSA, (**B**) St-1080@BSA, (**C**) FD-1080-Cl@BSA, and (**D**) free BSA against time.

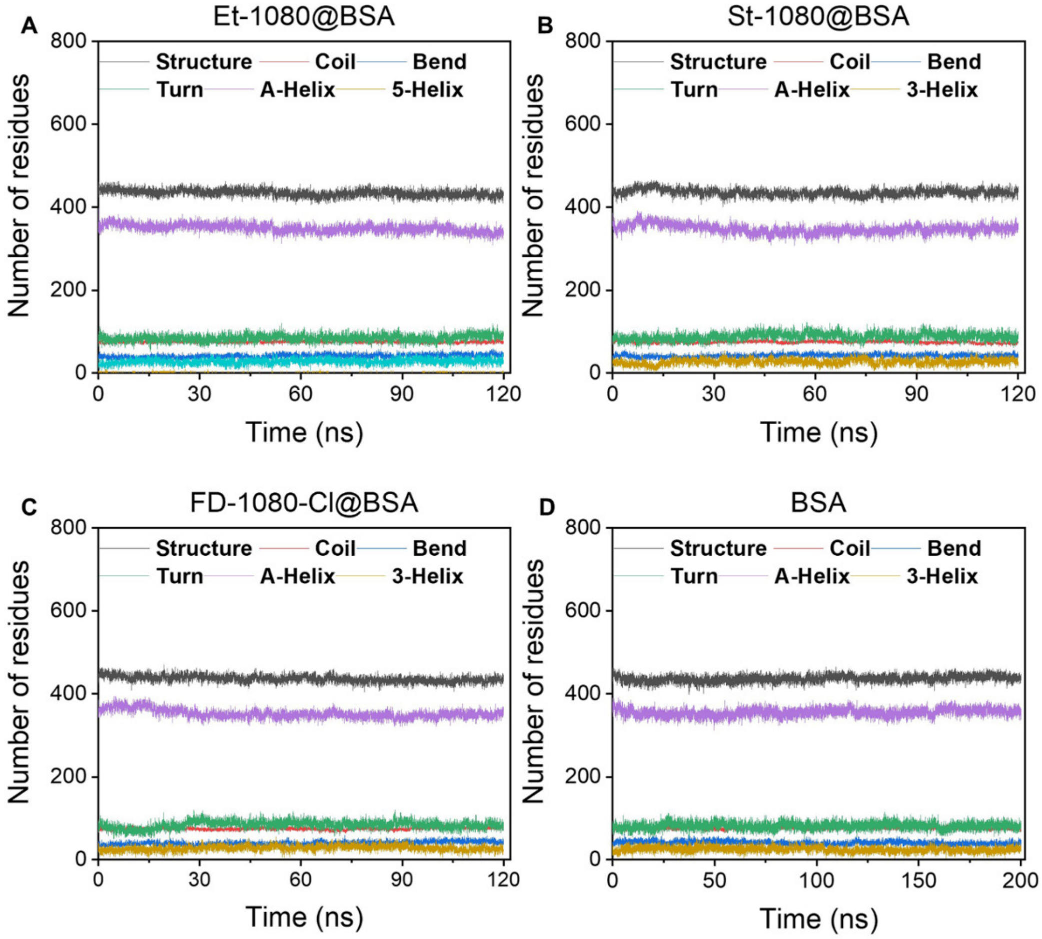

Figure S24. Numbers of residues in diverse secondary structures of (**A**) Et-1080@BSA, (**B**) St-1080@BSA, (**C**) FD-1080-Cl@BSA, and (**D**) free BSA.

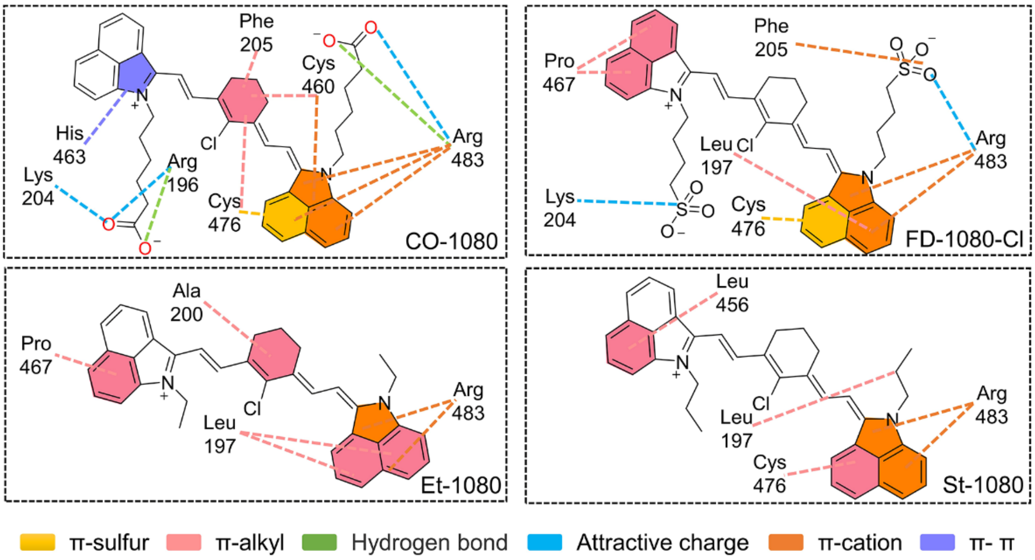

Figure S25. 2D diagram of interaction between CO-1080, FD-1080-Cl, Et-1080, St-1080 and BSA. Interactions involved in the binding of BSA to amino acid residues are shown as squares in different corresponding colors.

Note for Figure S25:

The 2D diagram reveals that St-1080 forms a π-alkyl interaction with Cys476, Leu197, and Leu456 residues of BSA, a π-cation interaction with Arg483 residues of BSA. Et-1080 interacts with Leu197, Ala200, and Pro467 residue of BSA via a π-alkyl interaction, and with Arg483 residue via a π-cation interaction. FD-1080-Cl exhibits multiple interactions with BSA. Specifically, it forms an electrostatic attraction interaction with Arg483 and Lys204 residue, a π-sulfur interaction with Cys476 residue, a π-alkyl interaction with Pro467 and Leu197 residues, a π-cation interaction with Phe205 and Arg483 residues. Similarly, CO-1080 interacts with Lys204, Arg196, and Arg483 residue via electrostatic attraction, with Cys476 residue via π-sulfur interaction, with Arg196 and Arg483 residues via hydrogen bond interaction, with Arg483 residue via π-cation interaction and with Cys476, Phe205 and Cys460 residues via π-alkyl interaction.

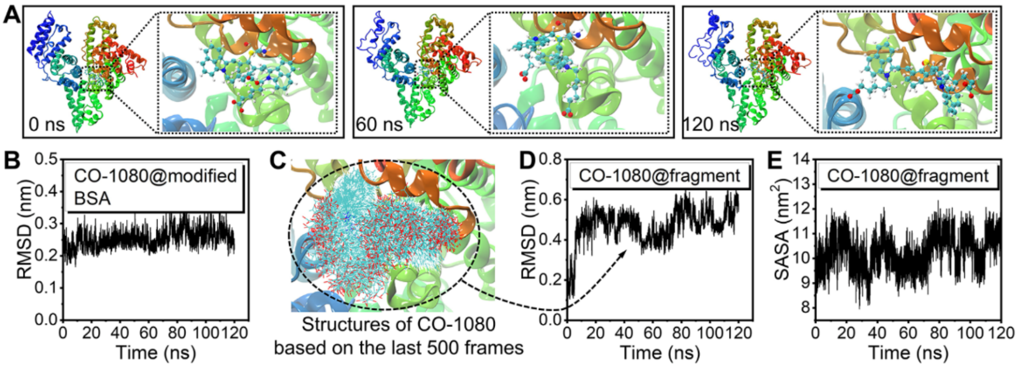

Figure S26. CO-1080 molecule covalently linked to Cys101 to obtain a modified BSA protein with a non-standard amino acid residue. (**A**) Structures of CO-1080 covalent binding to BSA with a non-standard amino acid residue, which were extracted from the molecular dynamics simulation trajectory at 0, 60, and 120 ns. (**B**) RMSD of the modified BSA protein against time. (**C**) Structures of this fragment superposition from the final 500 frames of the trajectory. (**D, E**) The RMSD and solvent-accessibility surface area (SASA) of this fragment.

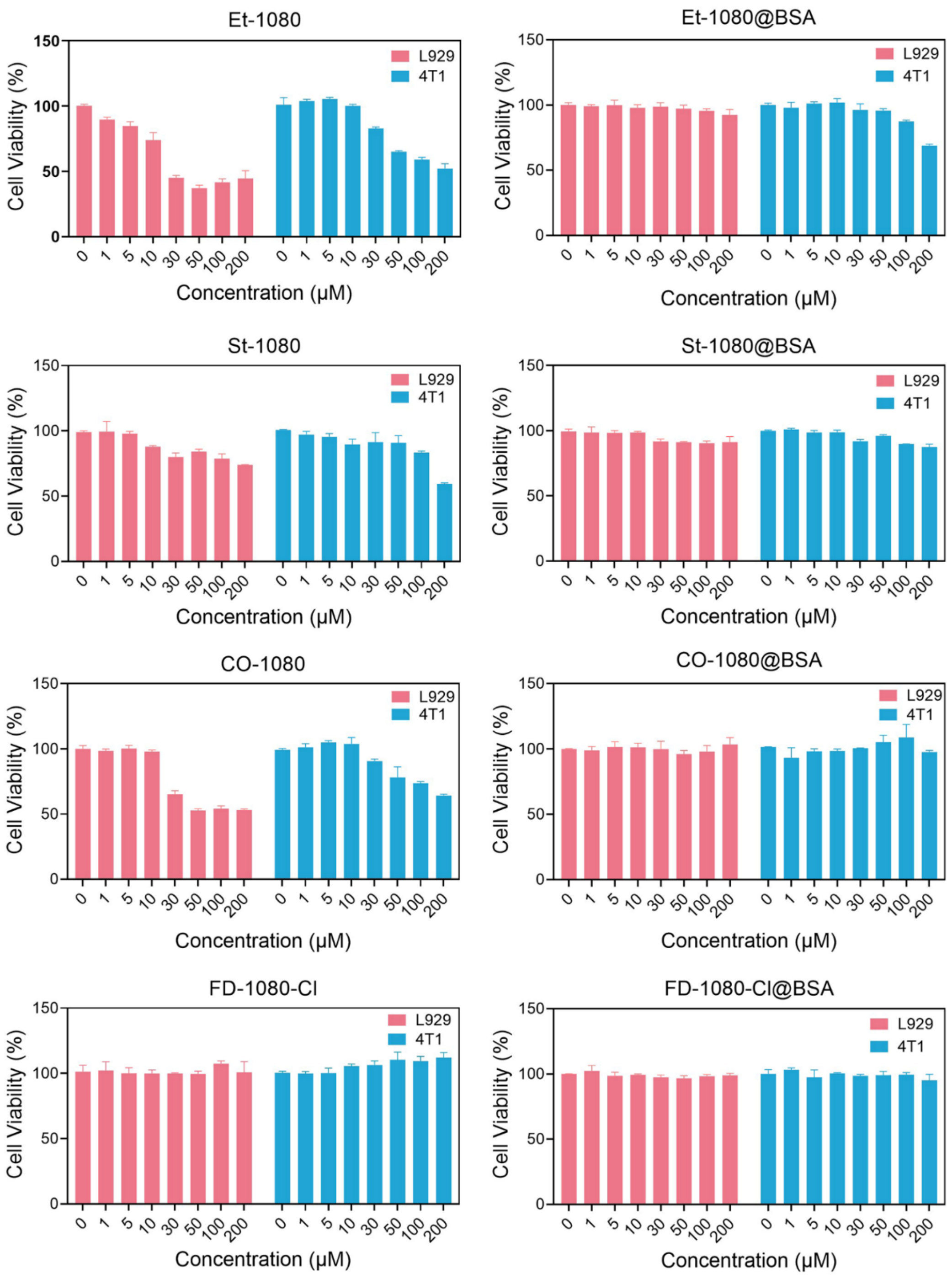

Figure S27. Cytotoxicity of 1080 dyes and 1080@BSA probes at different concentrations on the mouse fibroblasts (L929) and breast cancer (4T1) cell lines.

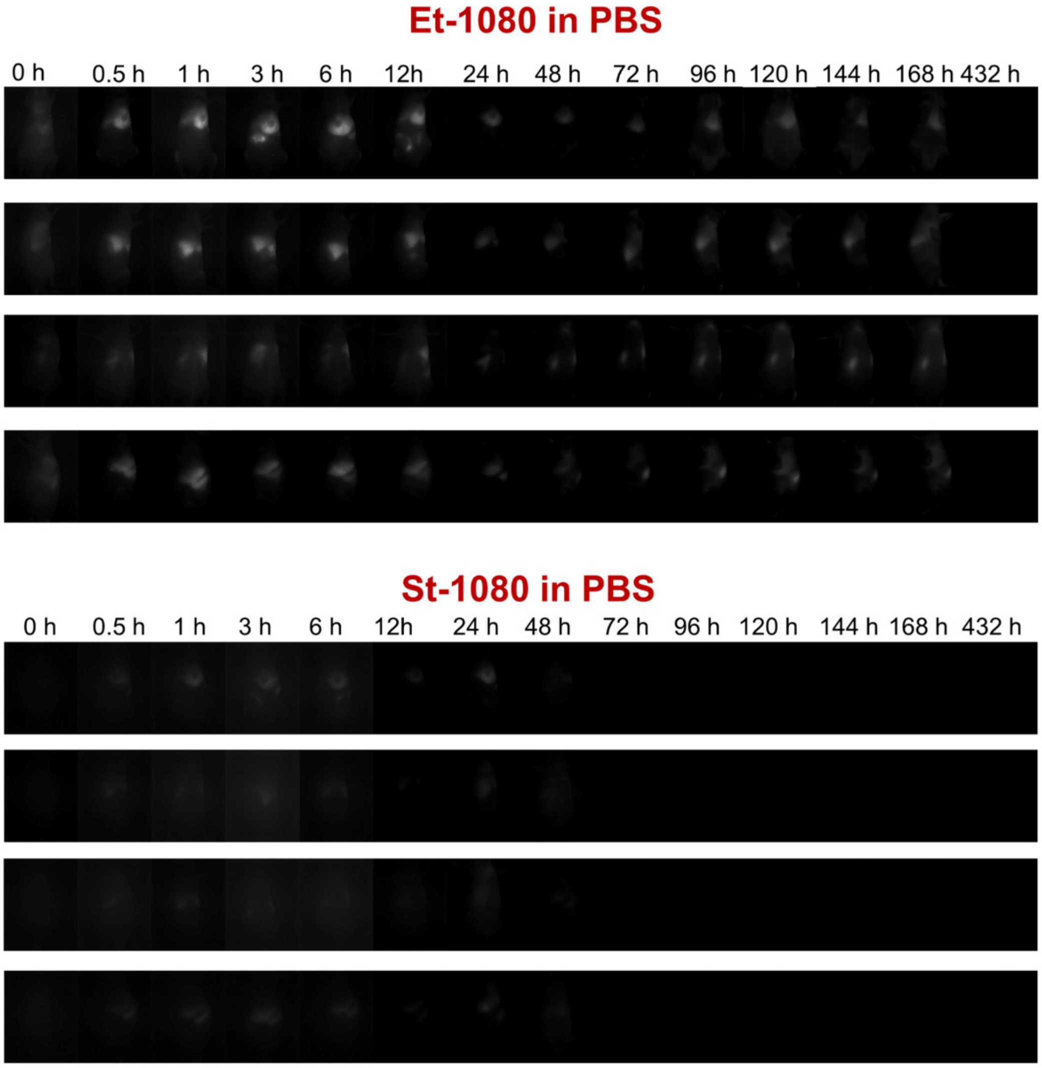
Et-1080 in PBS
0 h
0.5 h
1 h
3 h
6 h
12h
24 h
48 h
72 h
96 h
120 h
144 h
168 h
432 h
St-1080 in PBS
0 h
0.5 h
1 h
3 h
6 h
12h
24 h
48 h
72 h
96 h
120 h
144 h
168 h
432 h

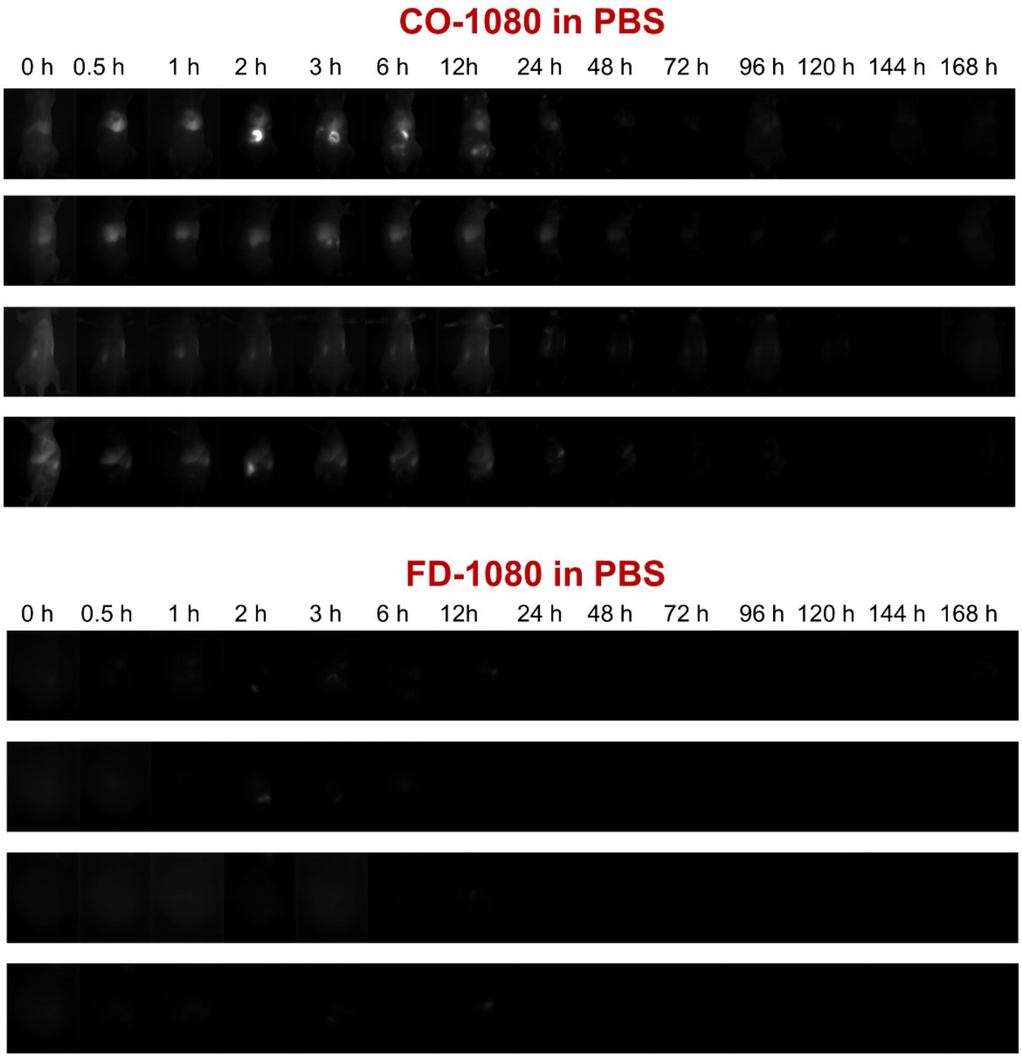
CO-1080 in PBS
0 h
0.5 h
1 h
2 h
3 h
6 h
12h
24 h
48 h
72 h
96 h
120 h
144 h
168 h
FD-1080 in PBS
0 h
0.5 h
1 h
2 h
3 h
6 h
12h
24 h
48 h
72 h
96 h
120 h
144 h
168 h

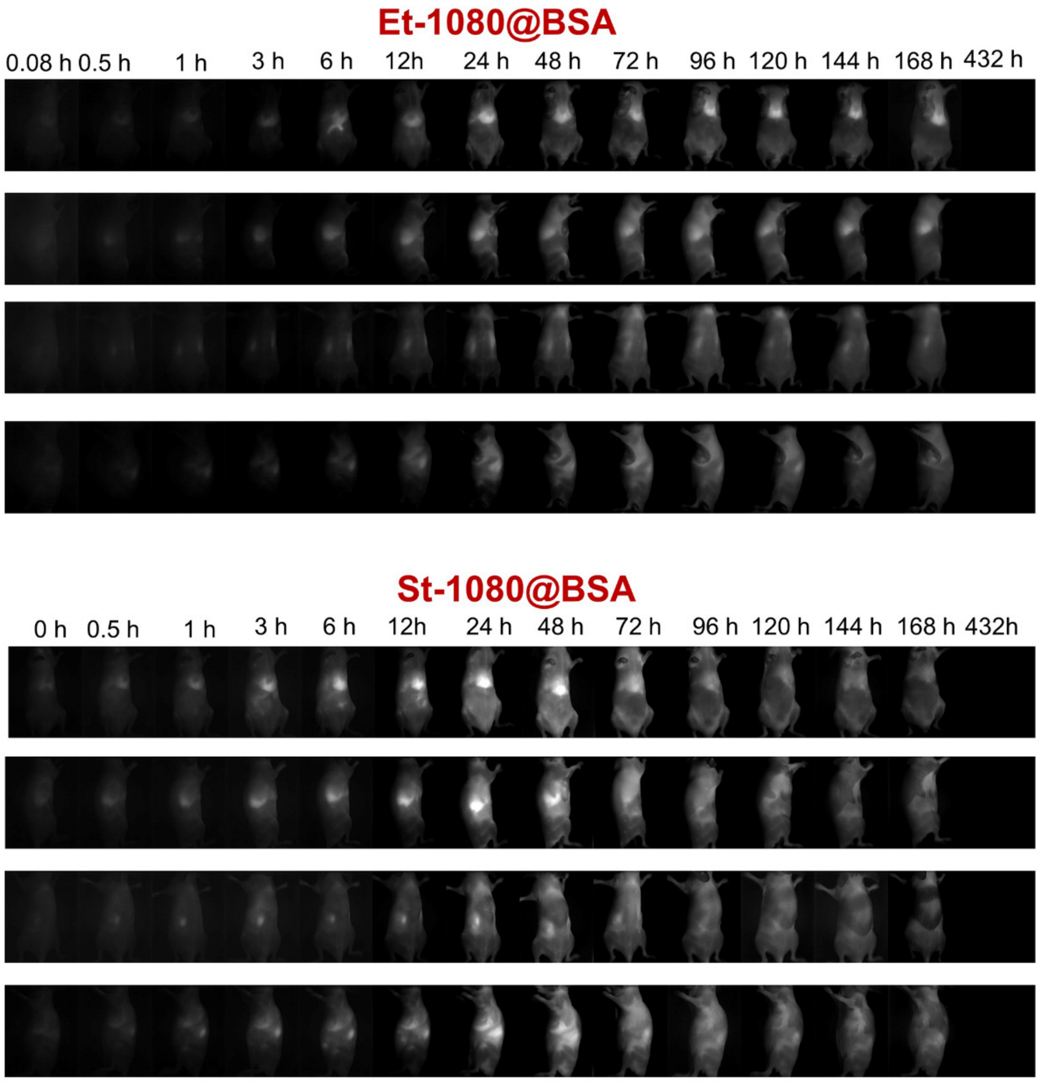
Et-1080@BSA
0.08 h 0.5 h 1 h 3 h 6 h 12h 24 h 48 h 72 h 96 h 120 h 144 h 168 h 432 h
St-1080@BSA
0 h 0.5 h 1 h 3 h 6 h 12h 24 h 48 h 72 h 96 h 120 h 144 h 168 h 432h

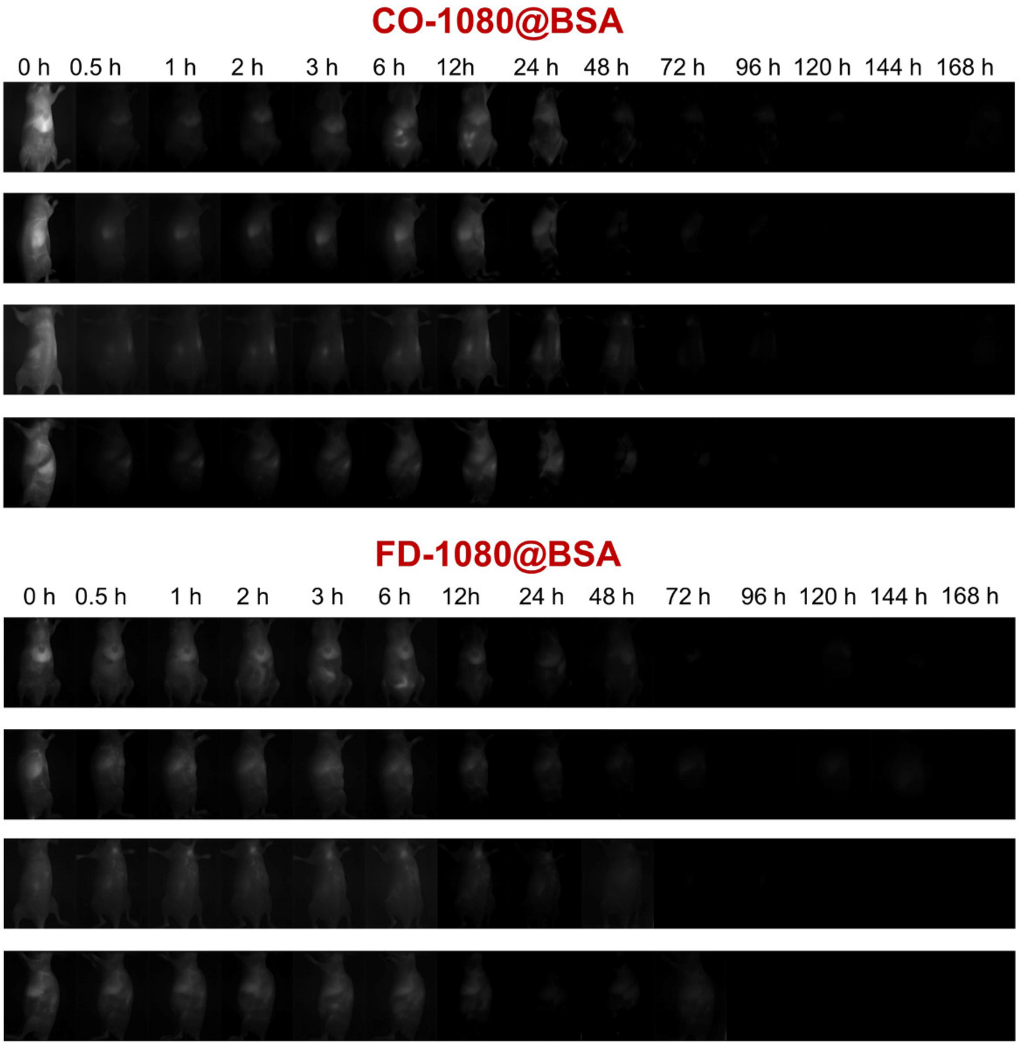

Figure S28. NIR-II whole-body imaging after tail vein injection of free dyes and dyes@BSA for different time points post-injection (200 μM, 200 μL, 1000 + 1100 LP).

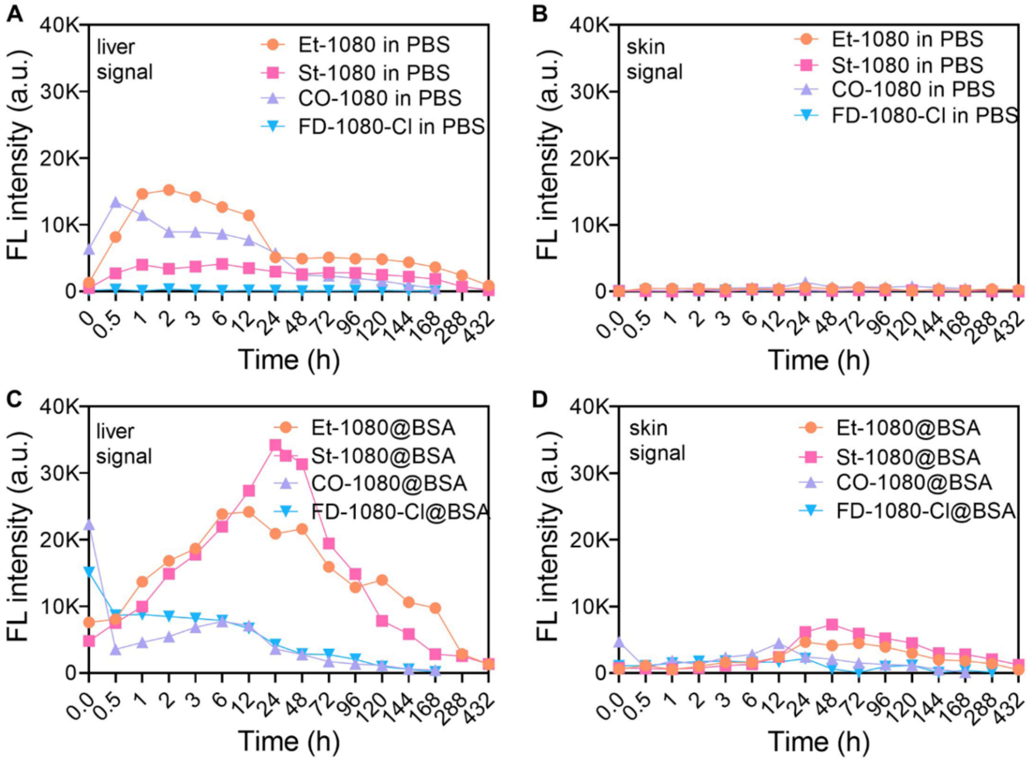

Figure S29. In vivo imaging using 1080 dyes and 1080@BSA probes. (**A, B**) Quantification of the liver and skin brightness in shaved Balb/c mice after intravenous administration of Et-1080, St-1080, CO-1080, and FD-1080-Cl. (**C, D**) Liver and skin signal quantification after intravenous injection of Et-1080@BSA, St-1080@BSA, CO-1080@BSA, and FD-1080-Cl@BSA probes in four cohorts of shaved Balb/c mice (200 μM, 200μL, imaging collected over 1100 nm).

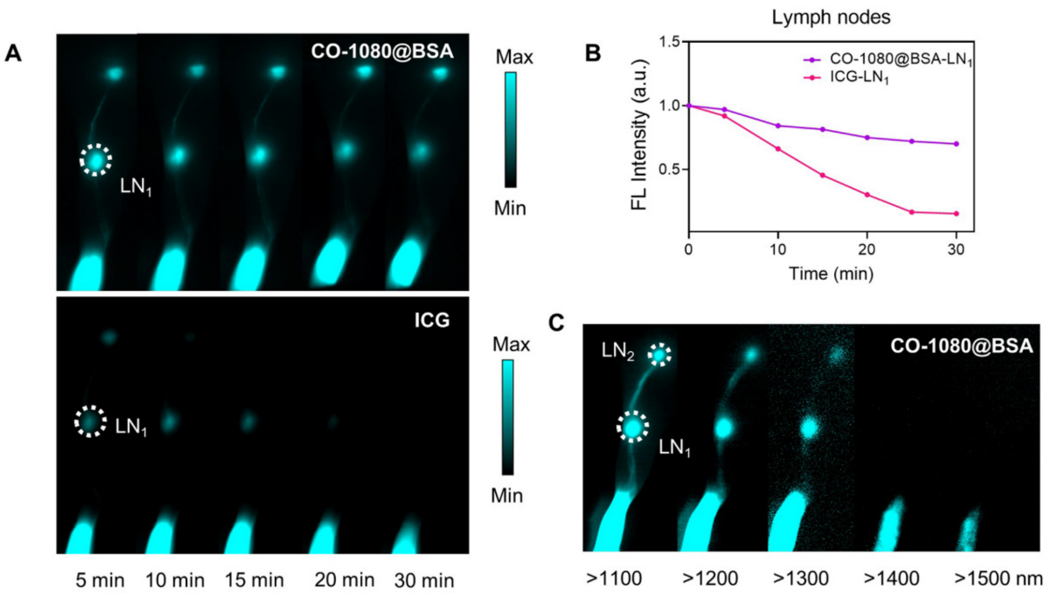

Figure S30. Imaging stability of CO-1080@BSA and ICG for NIR-II lymphography. (**A**) Comparison of lymphatic imaging between CO-1080@BSA probe and ICG with equivalent dosage (200 μM, 25 μL) during the first 30 minutes (imaging collected over 1100 nm). (**B**) Signal quantification in mice injected with CO-1080@BSA and ICG with LN_1 referring to popliteal lymph node (LN_1: popliteal lymph node). (**C**) NIR-II imaging of lymph node after injections of CO-1080@BSA (200 μM, imaging collected over 1100, 1200, 1300, 1400, and 1500 nm).

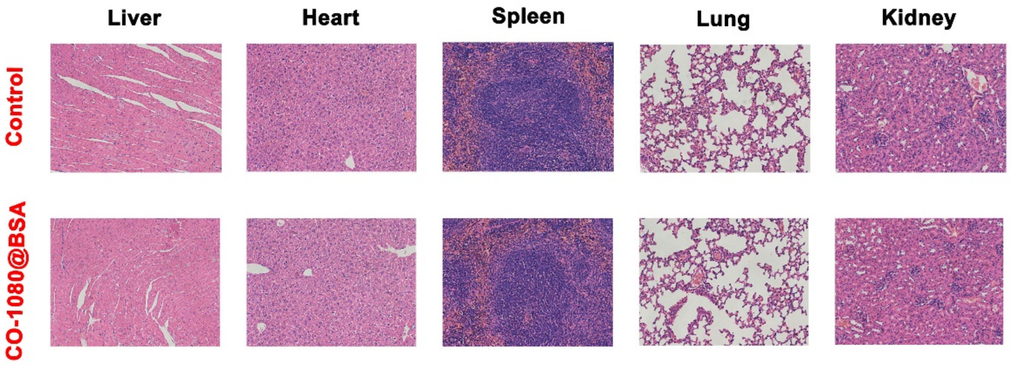

Figure S31. H&E staining analysis of main organ after administration of CO-1080@BSA and PBS.

Table S1. Optical properties between free 1080 dyes and dye@albumin.

Dye	$\lambda_{max,abs}$ (nm)	ε_{max} ($M^{-1}cm^{-1}$)	$\lambda_{max,em}$ (nm)	Stokes shift(nm)
Et-1080 in DMSO	1040	0.96×10^5	1070	30
Et-1080@BSA	1039	0.211×10^5	1055	16
St-1080 in DMSO	1044	1.003×10^5	1074	30
St-1080@BSA	1042	0.200×10^5	1058	16
CO-1080 in DMSO	1046	1.077×10^5	1077	33
CO-1080@BSA	1046	0.834×10^5	1063	17
FD-1080-Cl in DMSO	1046	0.733×10^5	1076	30
FD-1080-Cl@BSA	1048	0.434×10^5	1059	11

Table S2. CO-1080 modified peptide information.

Position	Peptide	Observed m/z	z	Mass error (ppm)	Score	Scan Time	Intensity
500	K.CC[+661.307]TESLVNR.R	562.600	3	13.5	245.0	10.5783	121170000
125	R.NEC[+661.307]FLSHKDDSPDLPK.L	627.289	4	-7.9	23.3	22.1879	53691000

Table S3. Blood routine tests of mice after administration of PBS and CO-1080@BSA.

		Control	CO-1080@BSA	Reference Range
WBC	(10^9/L)	5.633±2.274	2.033±0.416	0.8-10.6
Lymph	(10^9/L)	4.133±2.06	1.467±0.289	0.6-8.9
Mon	(10^9/L)	0.167±0.058	0.067±0.058	0.04-1.4
Gran	(10^9/L)	1.333±0.252	0.5±0.2	0.23-3.6
Lymph	(%)	70.4±10.762	71.233±5.239	40-92
Mon	(%)	3.433±1.457	4.167±1.026	0.9-18
Gran	(%)	26.167±9.309	24.6±4.709	6.5-50

RBC	$(10^{12}/L)$	9.267±0.172	9.143±0.325	6.5-11.5
HGB	(g/L)	138.667±4.041	136.333±4.933	110-165
HCT	%	47.1±1.908	47.7±1.5	35-55
MCV	(fL)	50.867±1.25	52.2±0.781	41-55
MCH	(pg)	14.9±0.173	14.867±0.404	13-18
MCHC	(g/L)	294±6.557	285±4.359	300-360
RDW	%	15±0.854	14.833±0.306	12-19
PLT	$(10^{9}/L)$	794.333±183.023	497.667±130.485	400-1600
MPV	(fL)	6.6±0.1	6.733±0.208	4.0-6.2
PDW		17.133±0.321	17.3±0.1	12.0-17.5
PCT	%	0.523±0.113	0.336±0.097	0.100-0.780

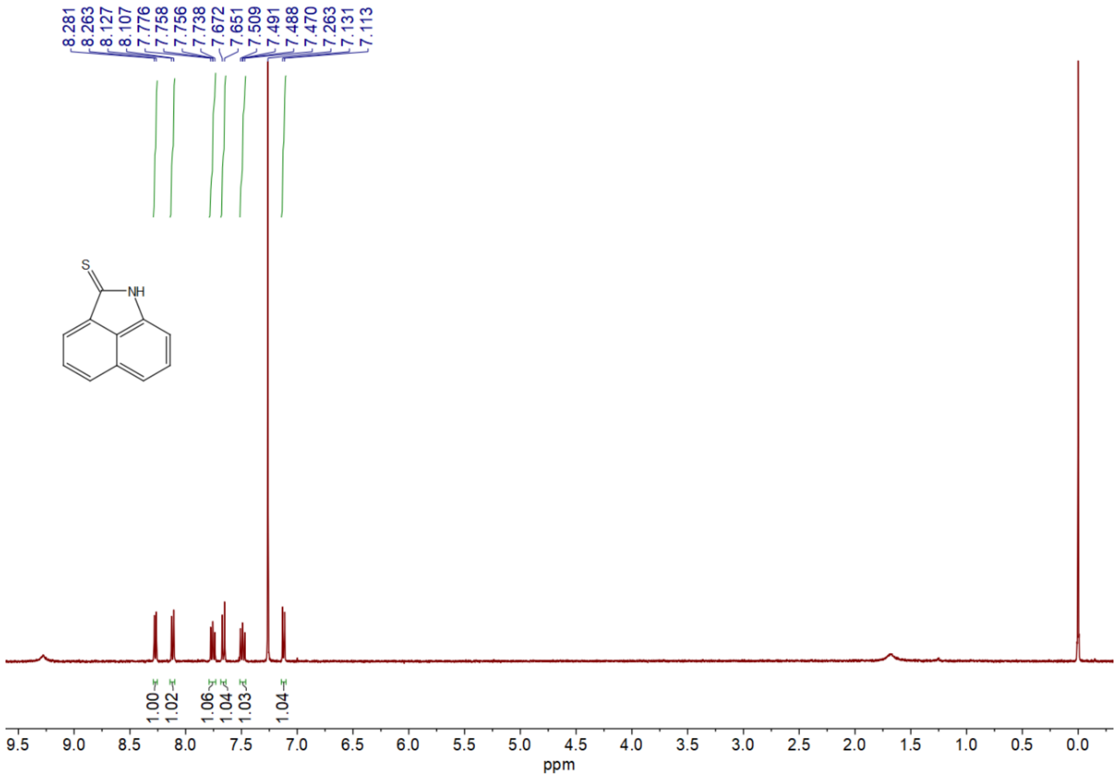

^{1}H-NMR spectrum of compound 1 in $CDCl_3$.

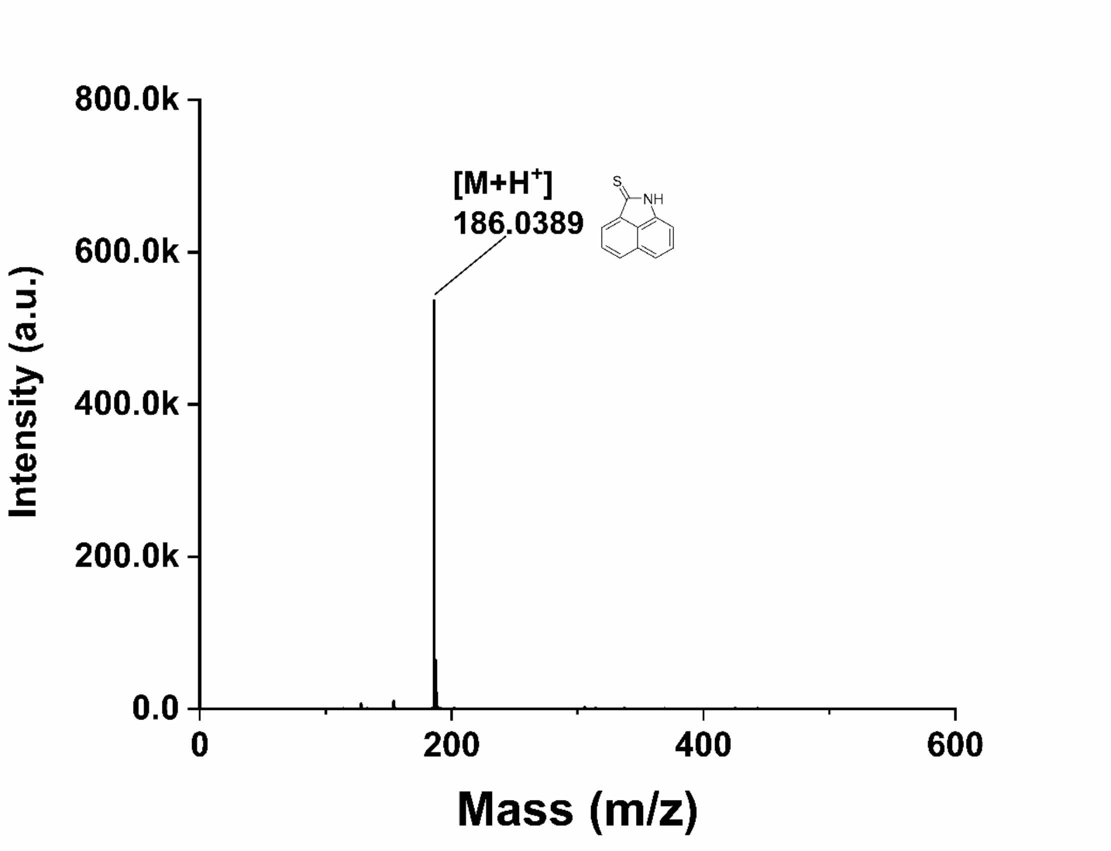

LC-HRMS spectra of the compound 1.

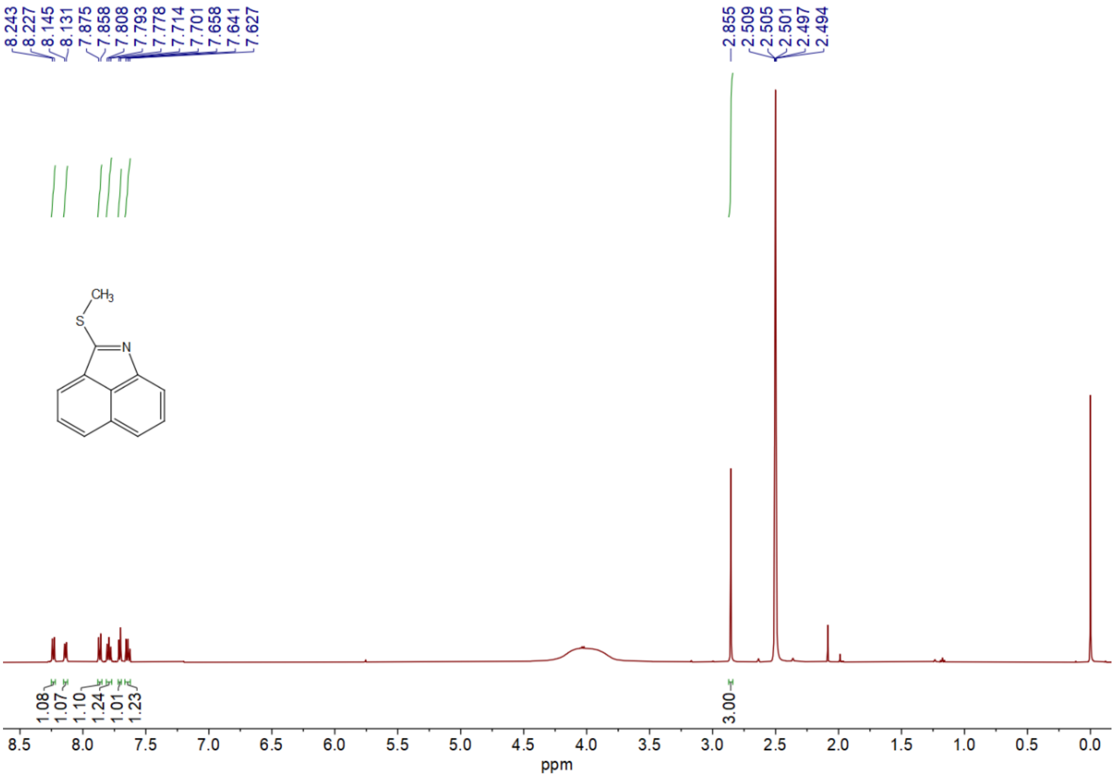

^{1}H-NMR spectrum of compound 2 in $CDCl_3$.

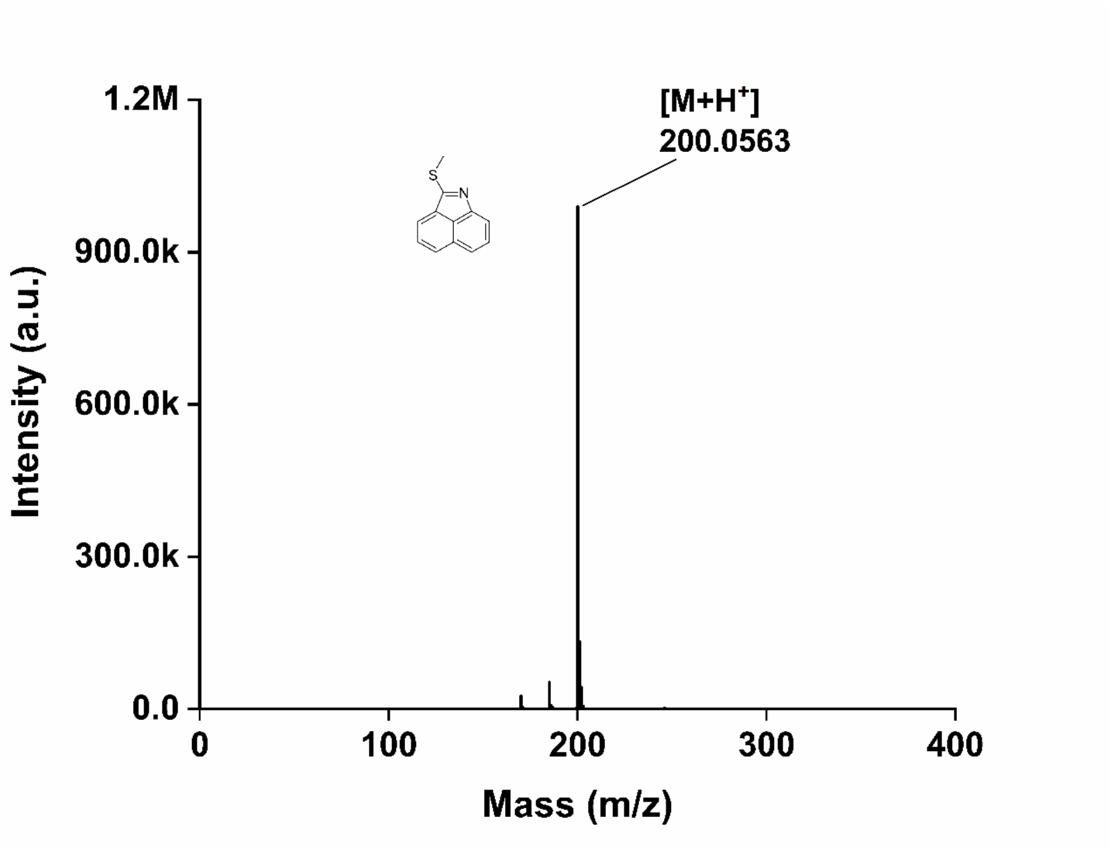

LC-HRMS spectra of the compound 2.

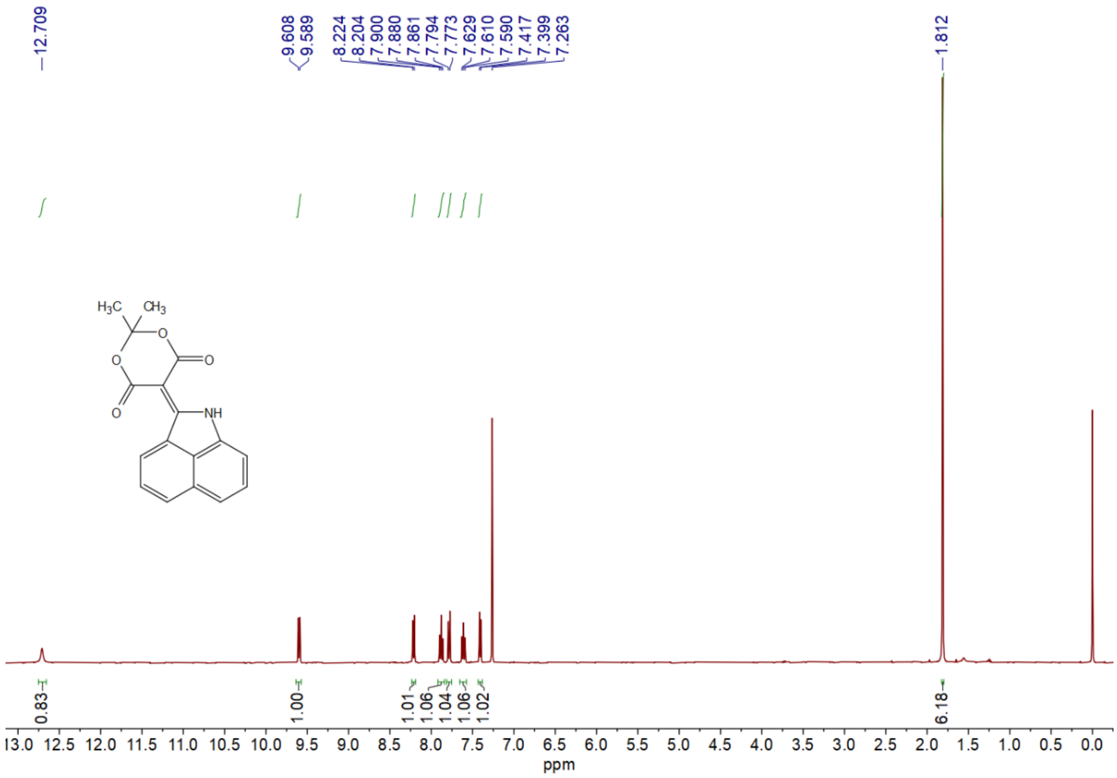

^{1}H-NMR spectrum of compound 3 in $CDCl_3$.

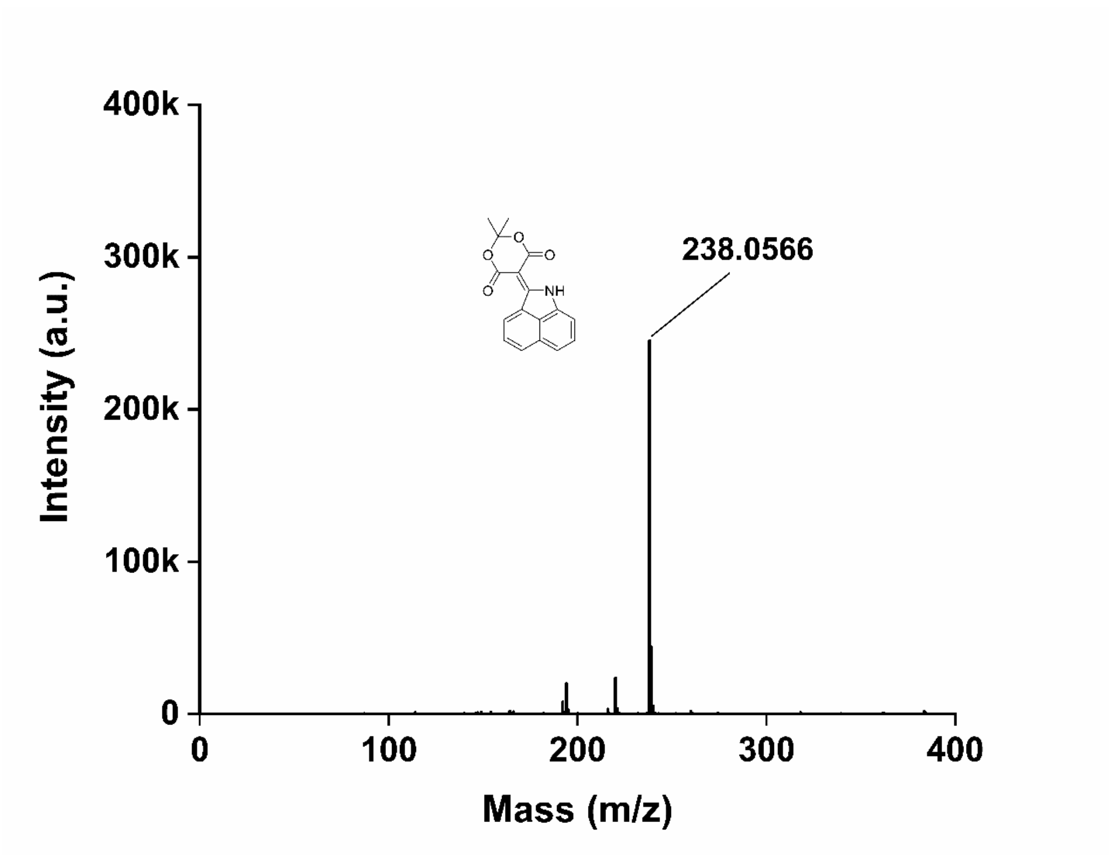

LC-HRMS spectra of the compound 3.

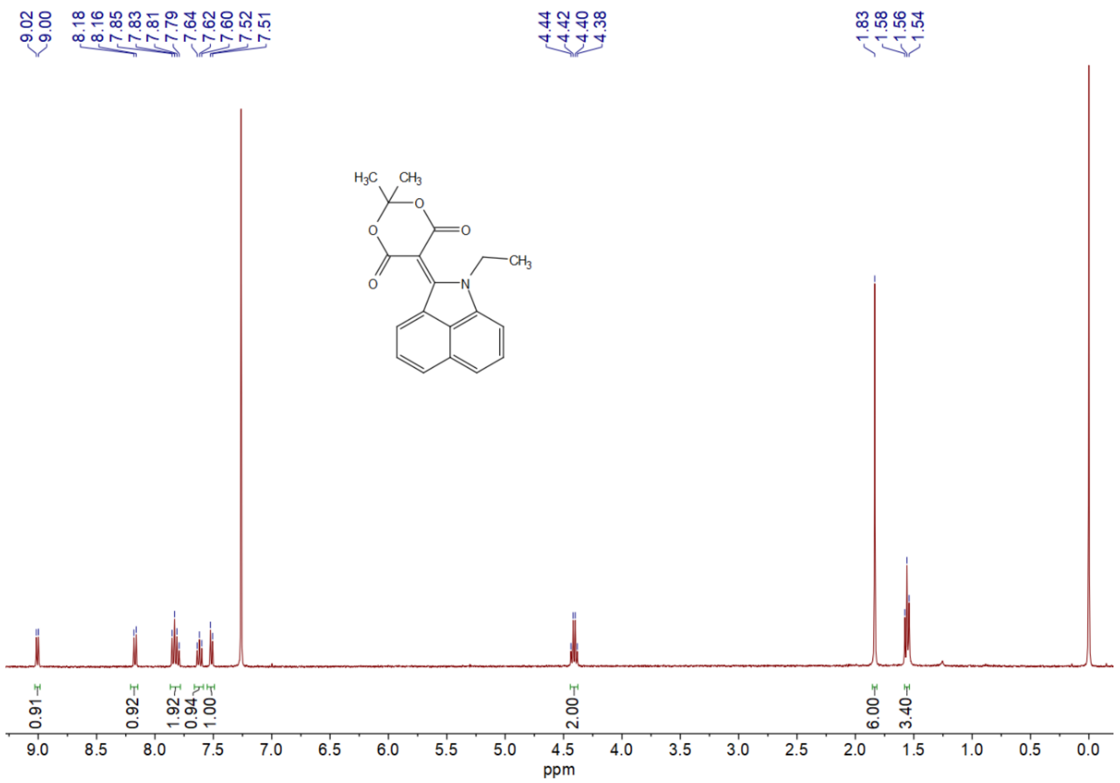

^{1}H-NMR spectrum of compound 5 in $CDCl_3$.

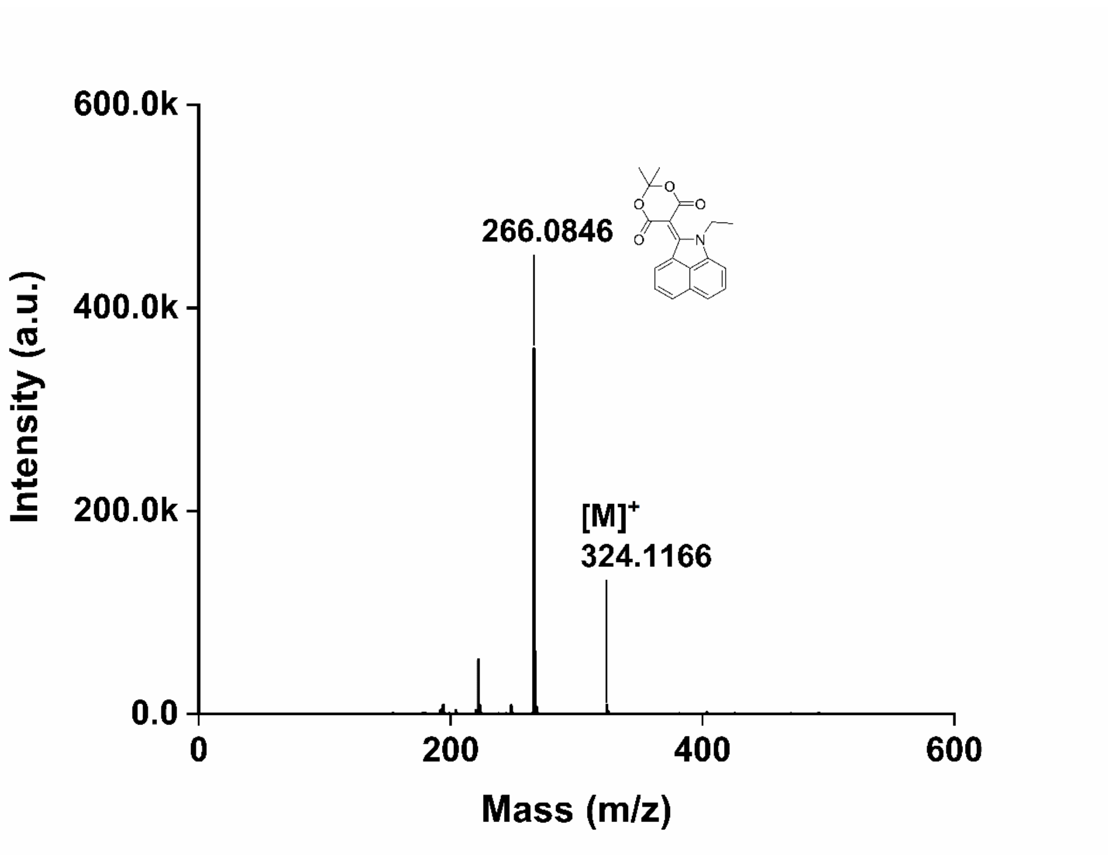

LC-HRMS spectra of the compound 5.

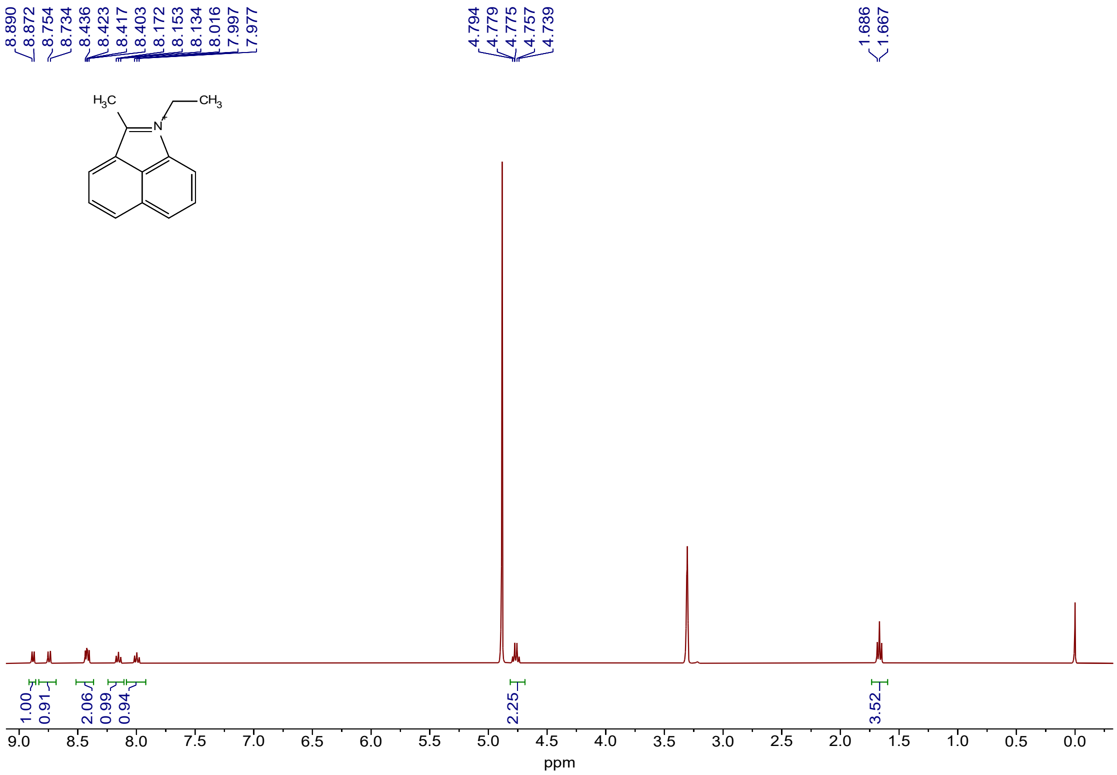

^{1}H-NMR spectrum of compound 6 in CD_3OD.

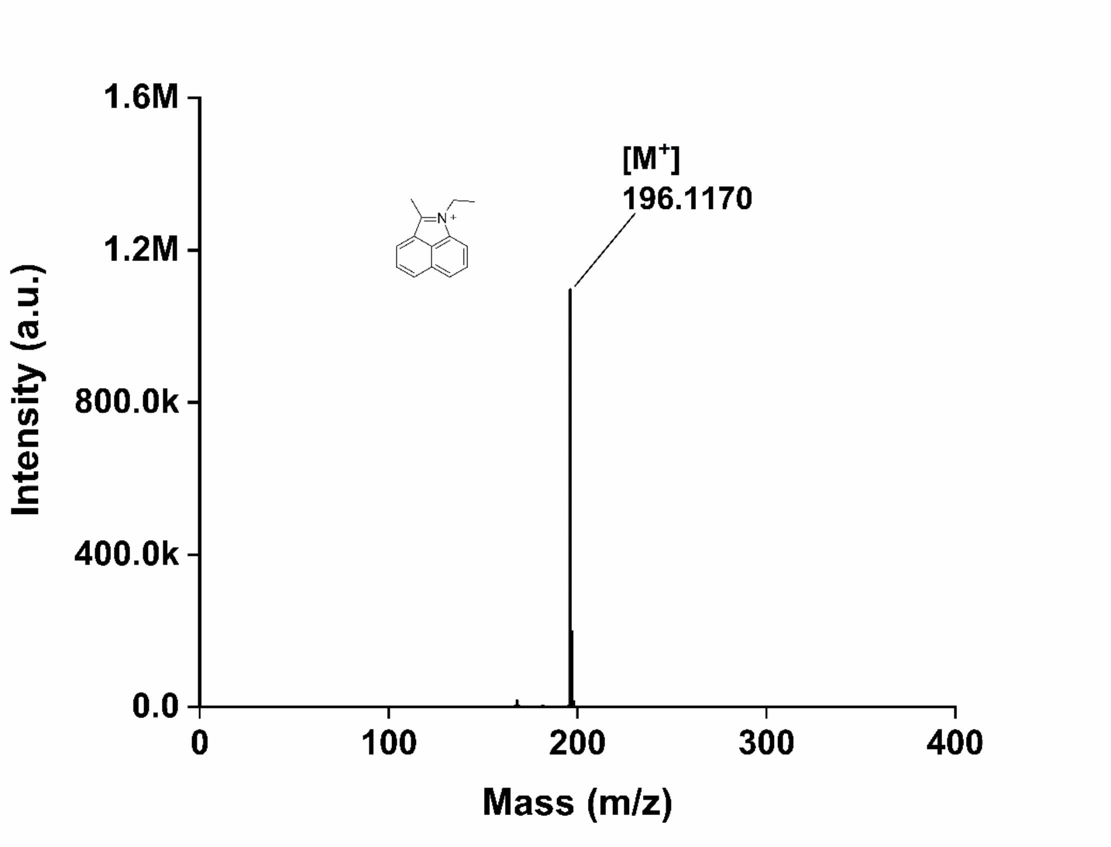

LC-HRMS spectra of the compound 6.

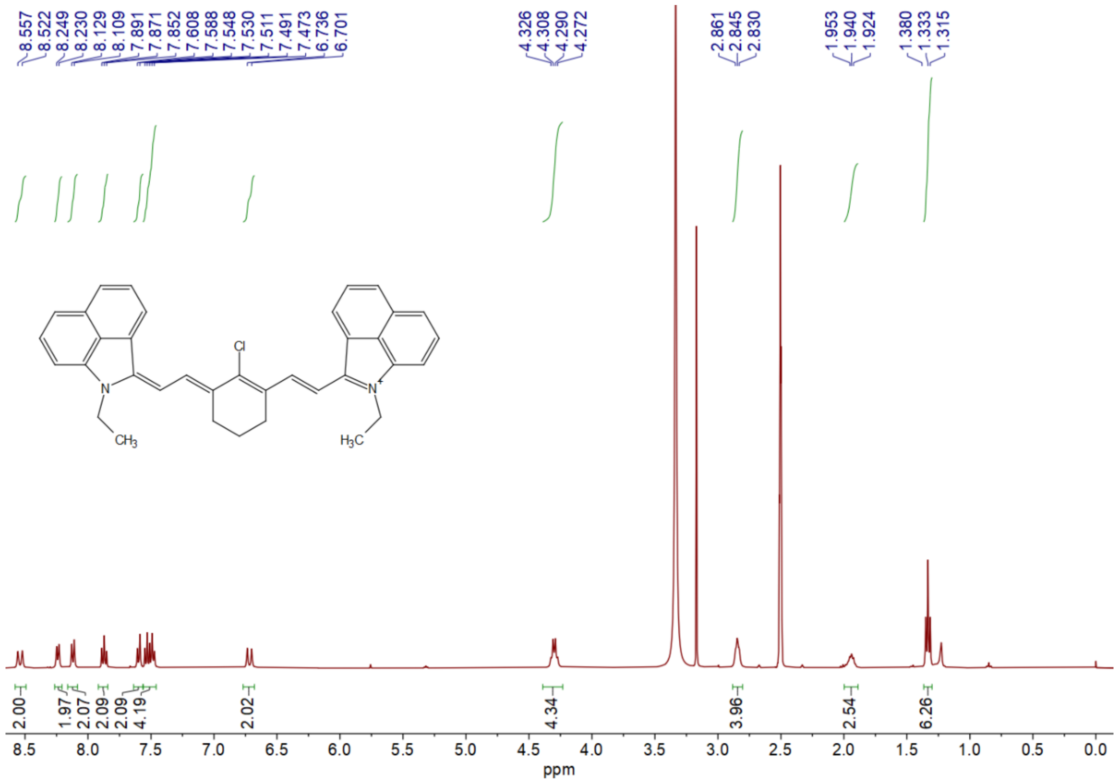

^{1}H-NMR spectrum of compound 7 in DMSO (Et-1080).

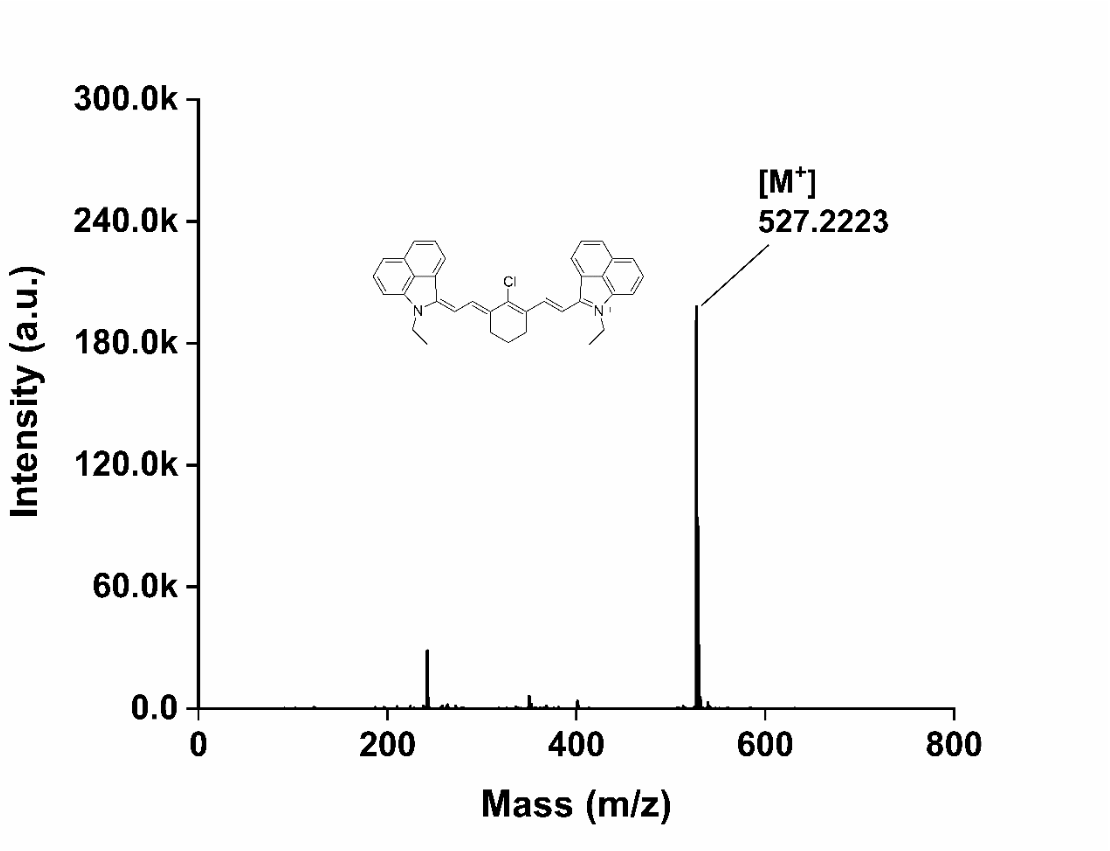

LC-HRMS spectra of the compound 7 (Et-1080).

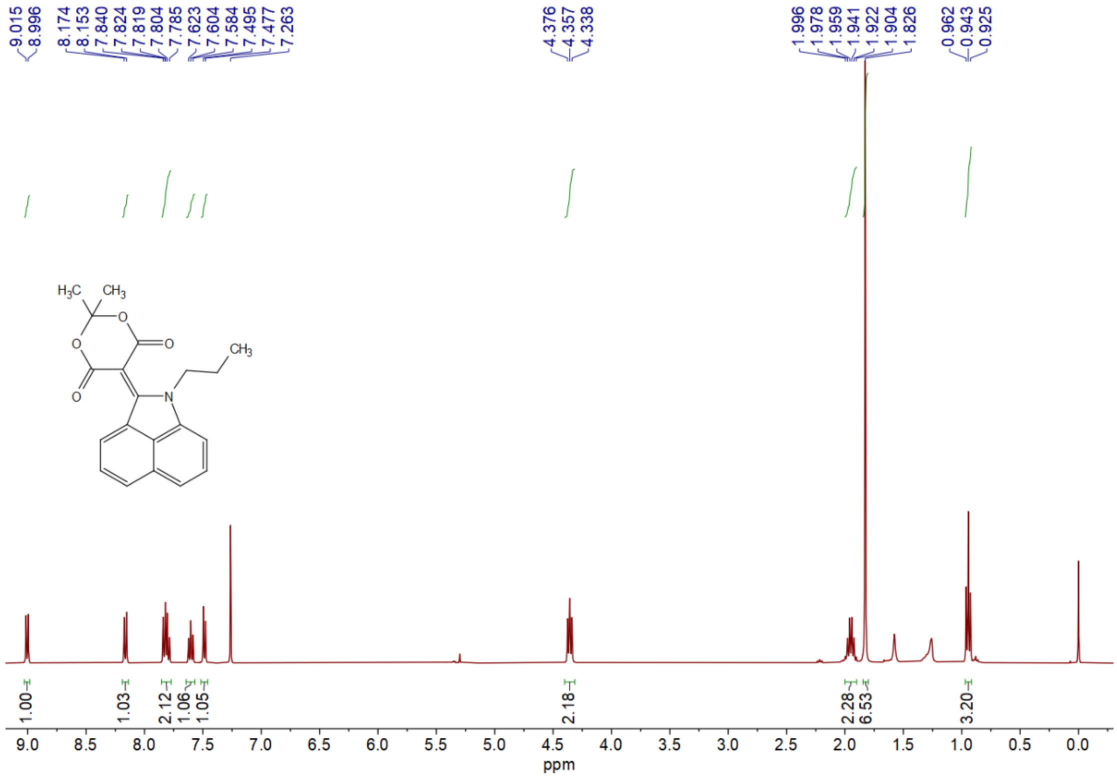

^{1}H-NMR spectrum of compound 8 in $CDCl_3$.

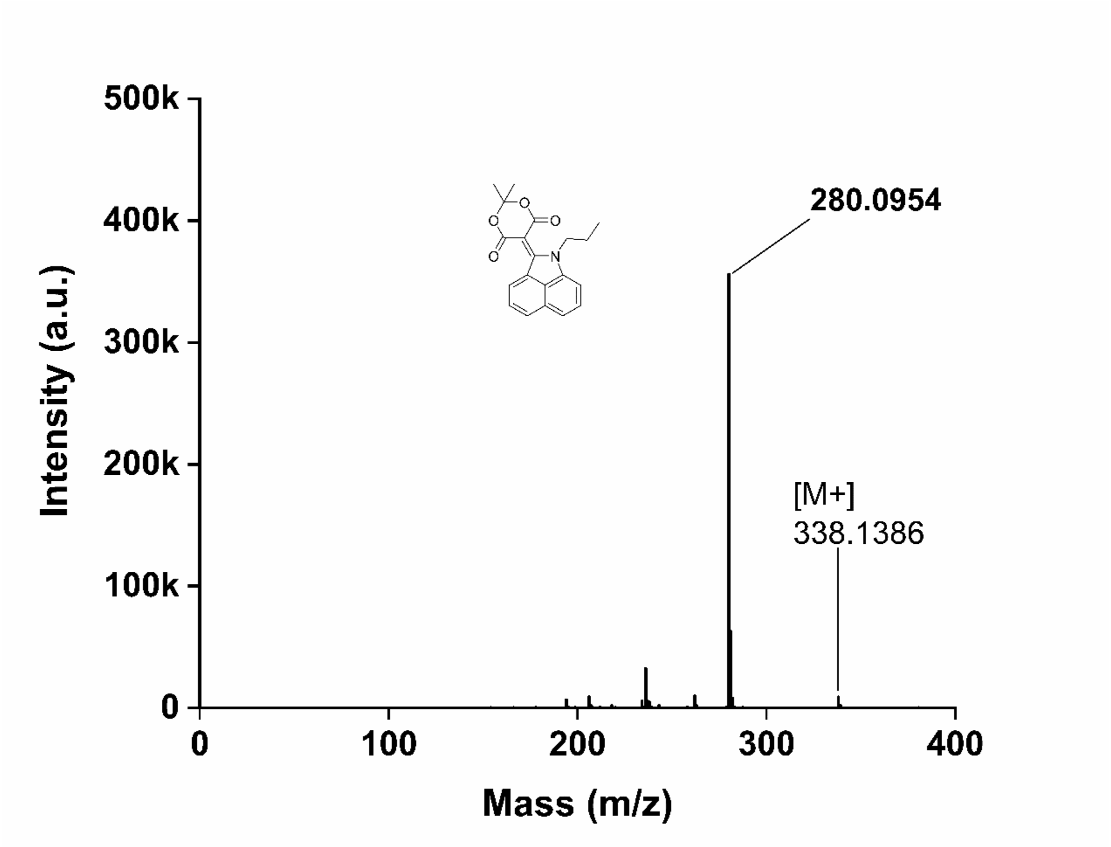

LC-HRMS spectra of the compound 8.

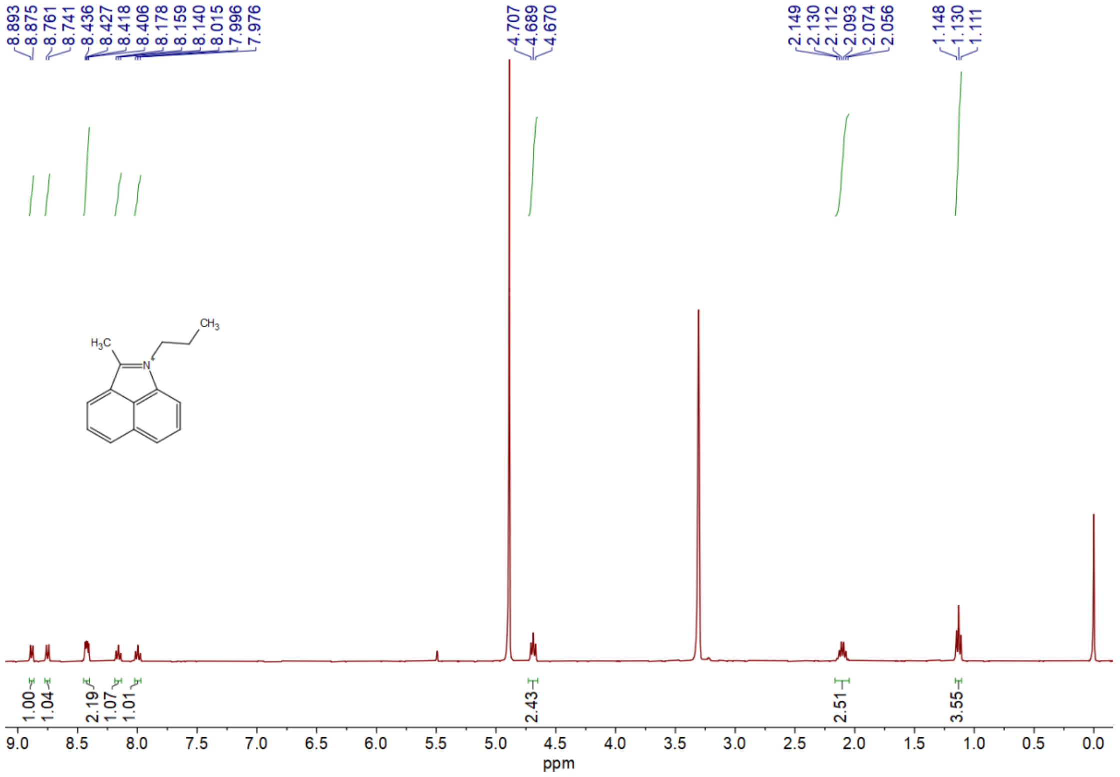

^{1}H-NMR spectrum of compound 9 in CD_3OD.

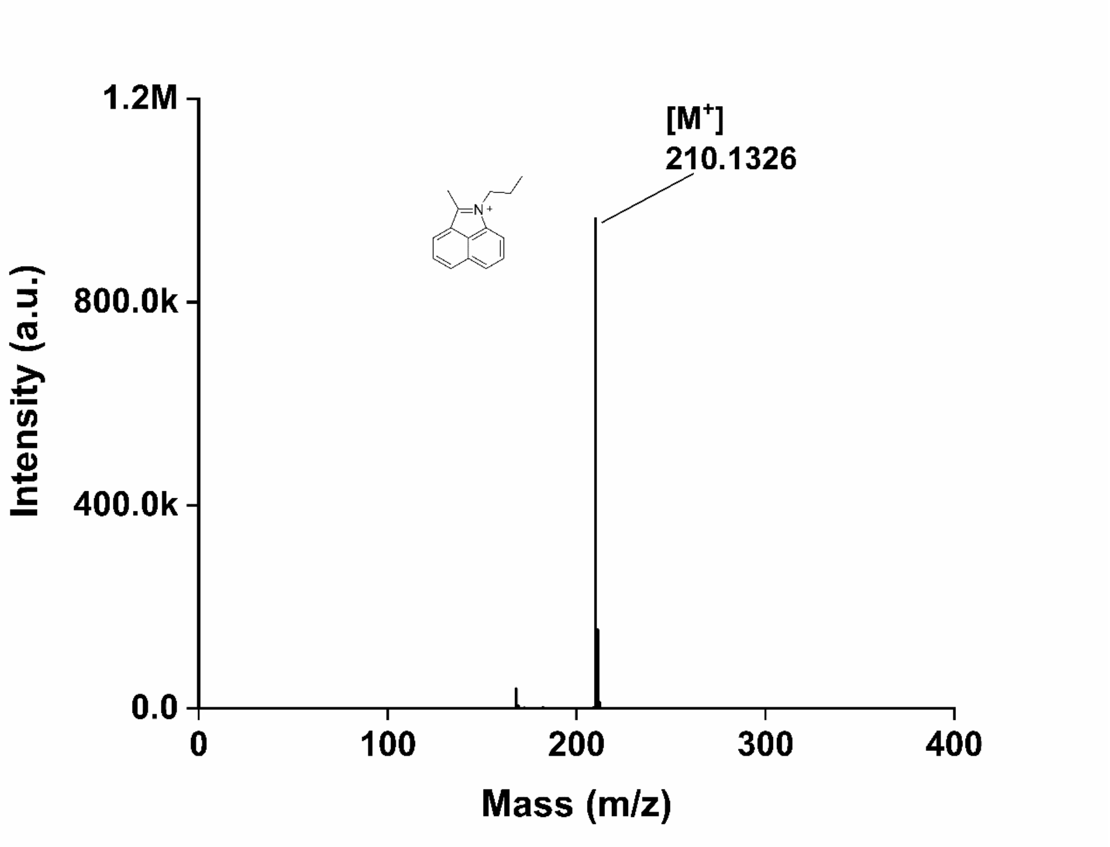

LC-HRMS spectra of the compound 9.

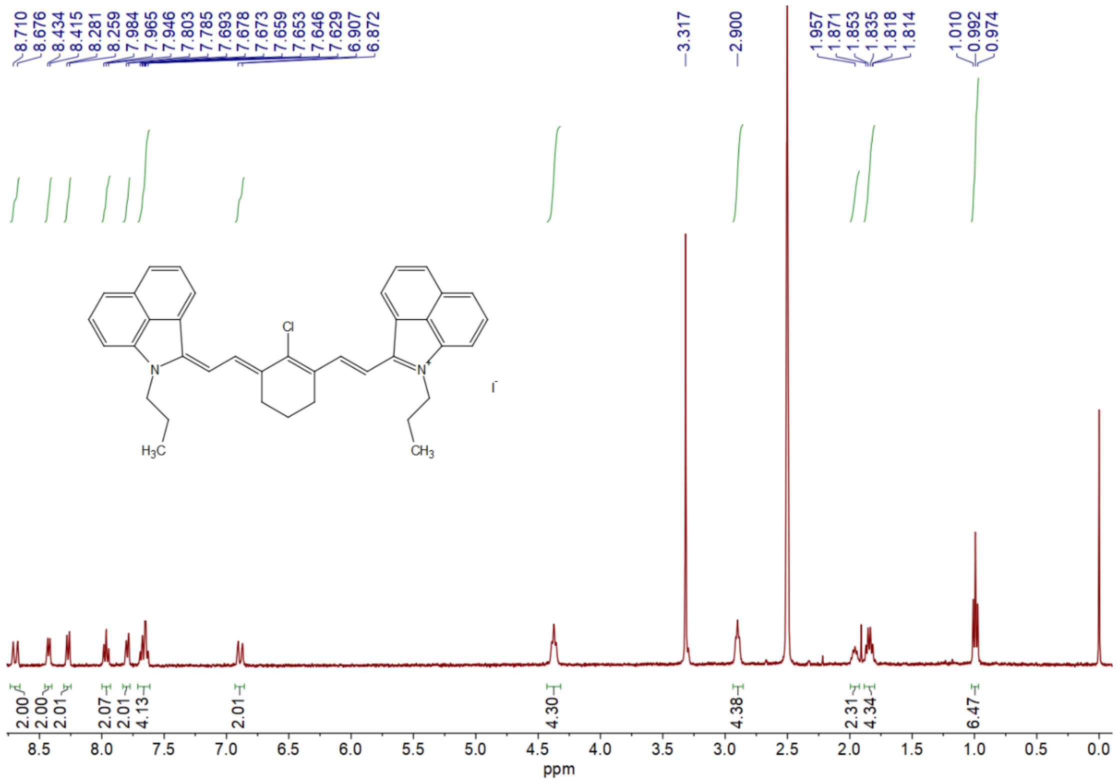

^{1}H-NMR spectrum of compound 10 in DMSO (St-1080).

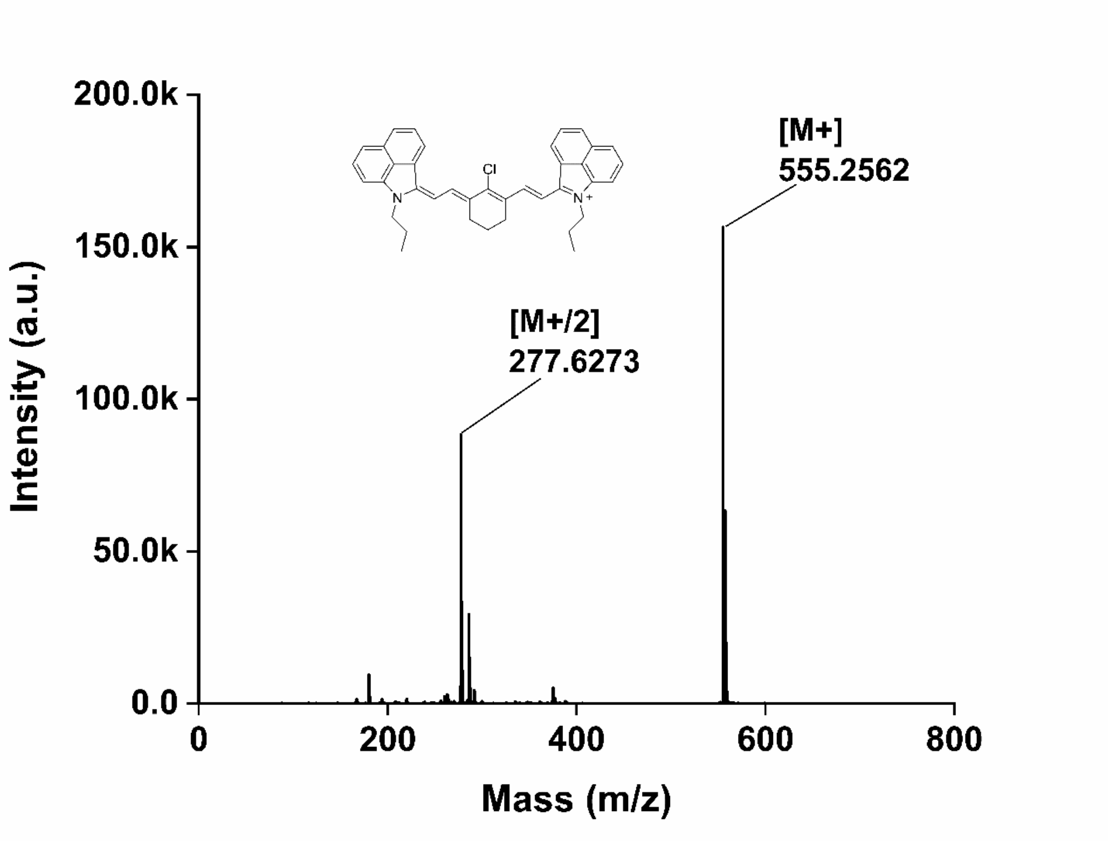

LC-HRMS spectra of the compound 10 (St-1080).

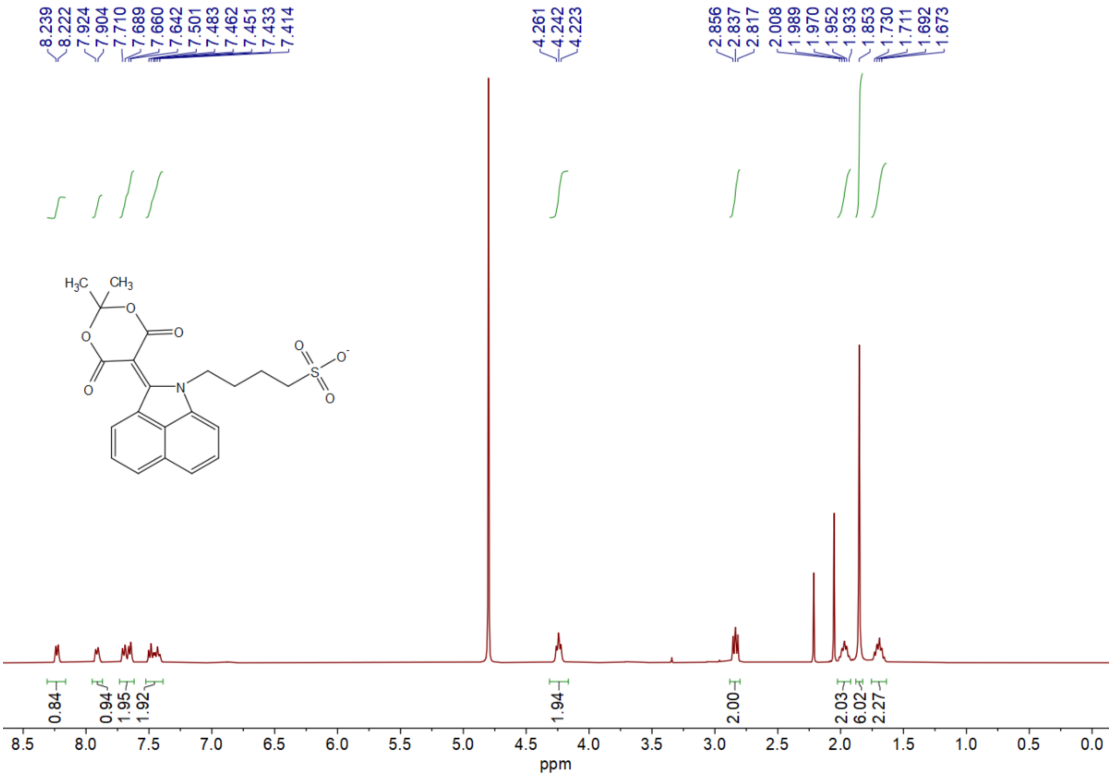

^{1}H-NMR spectrum of compound 11 in D_2O.

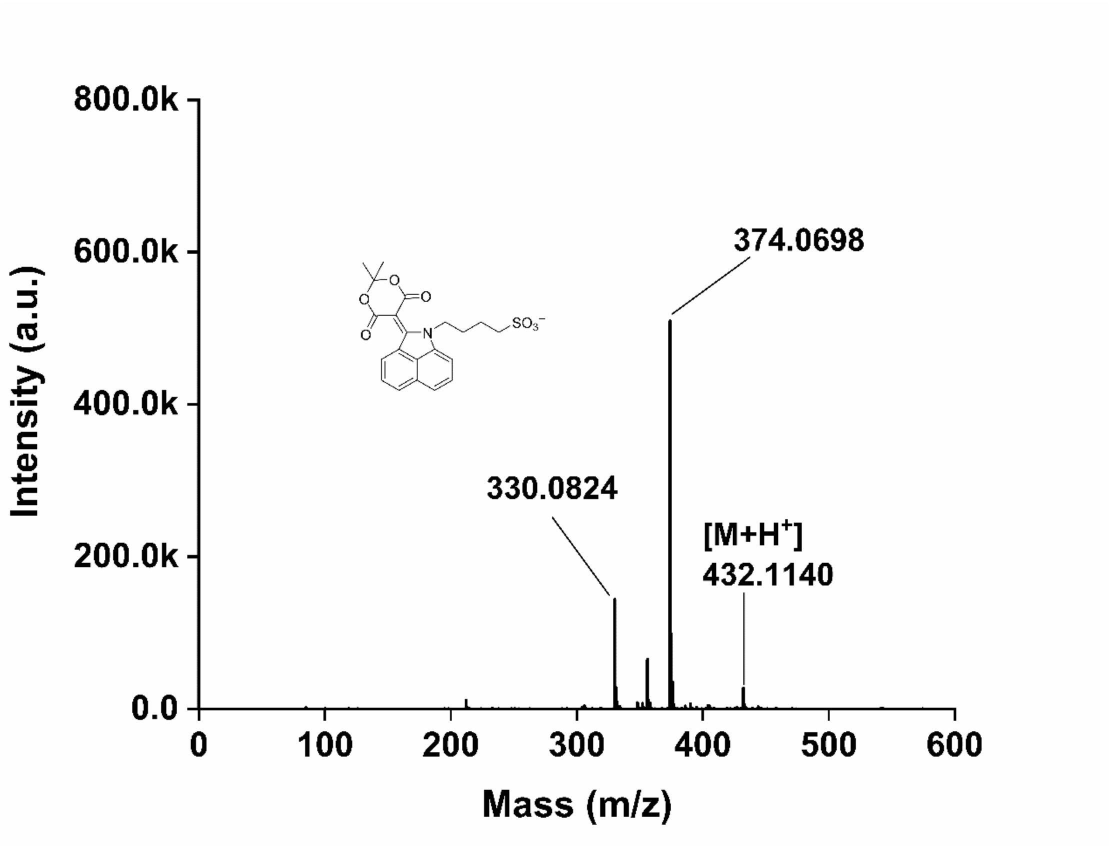

LC-HRMS spectra of the compound 11.

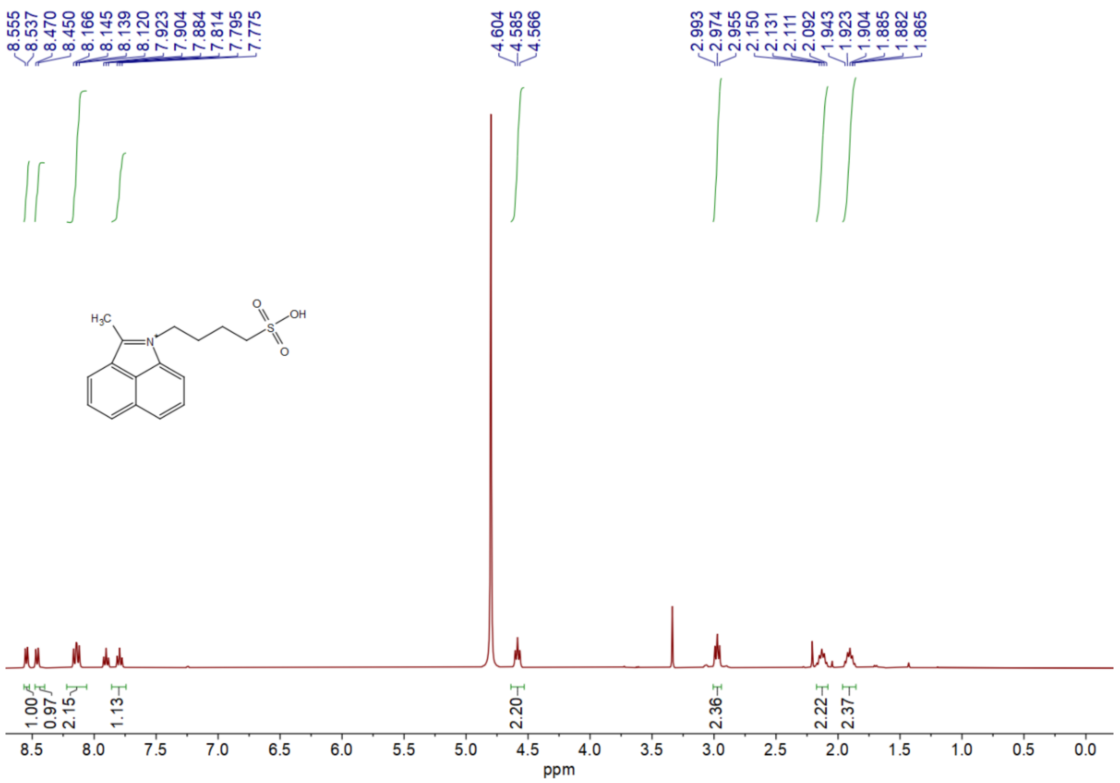

^{1}H-NMR spectrum of compound 12 in D_2O.

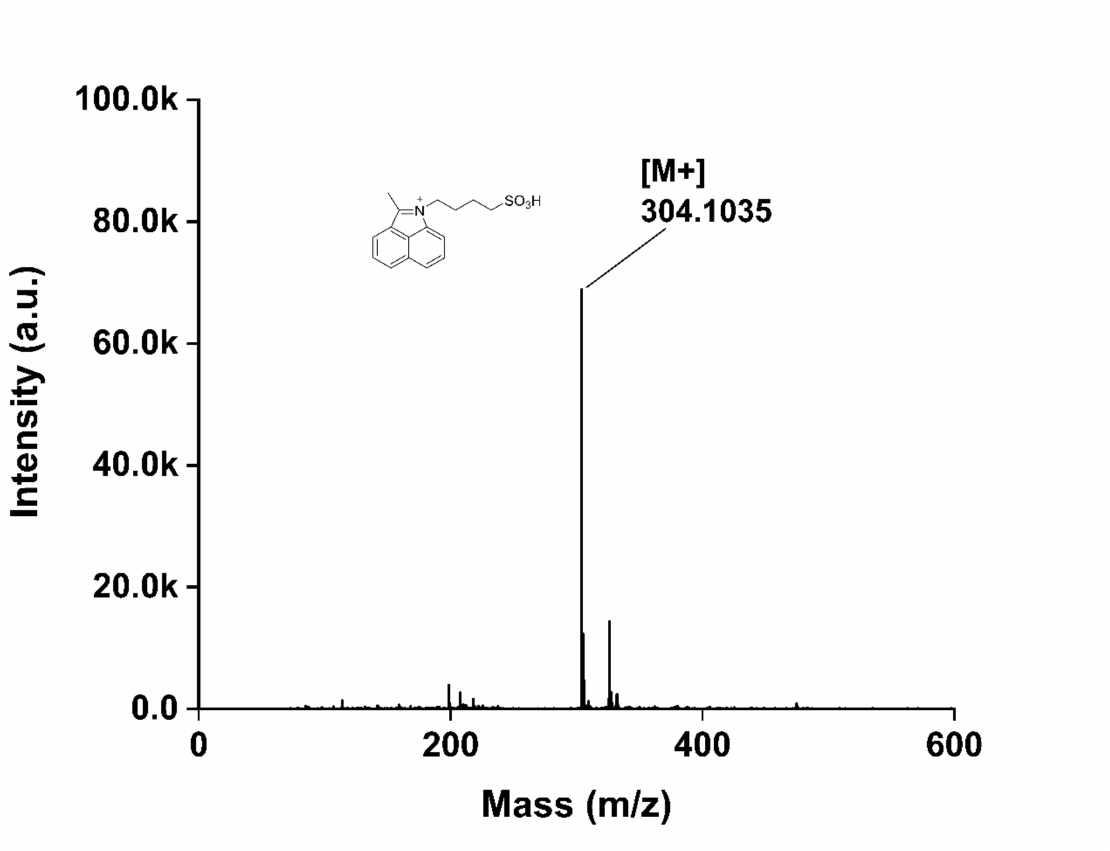

LC-HRMS spectra of the compound 12.

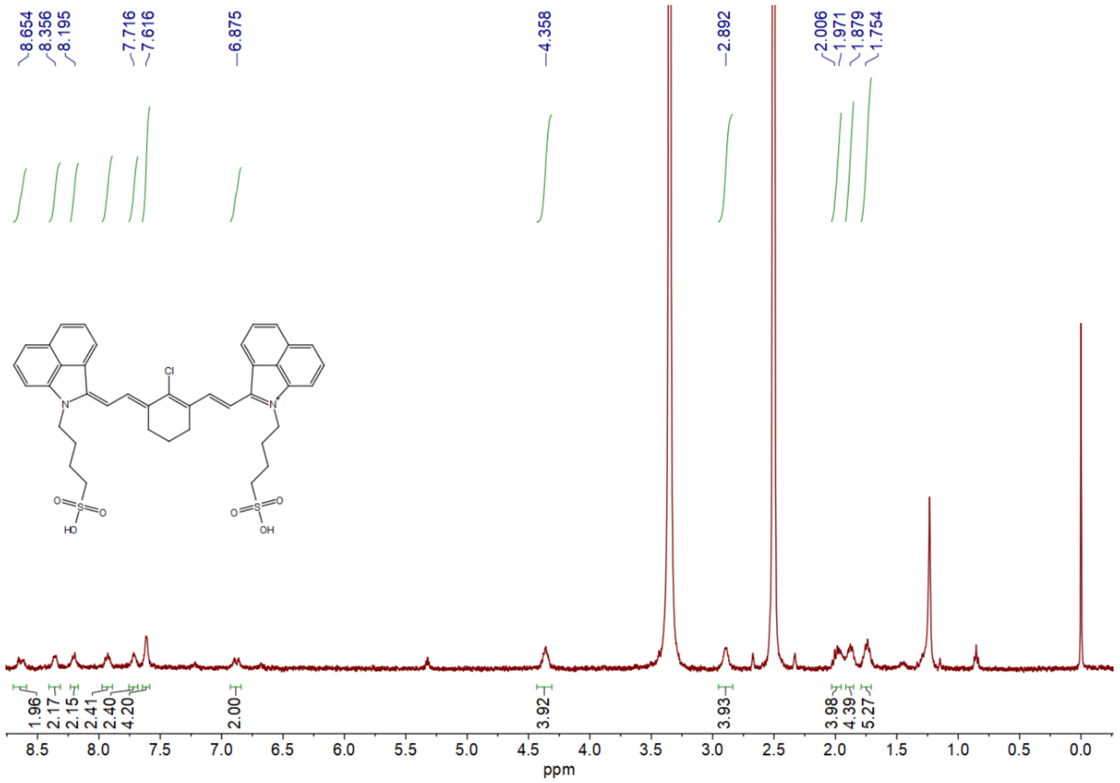

^{1}H-NMR spectrum of compound 13 in DMSO (FD-1080-Cl).

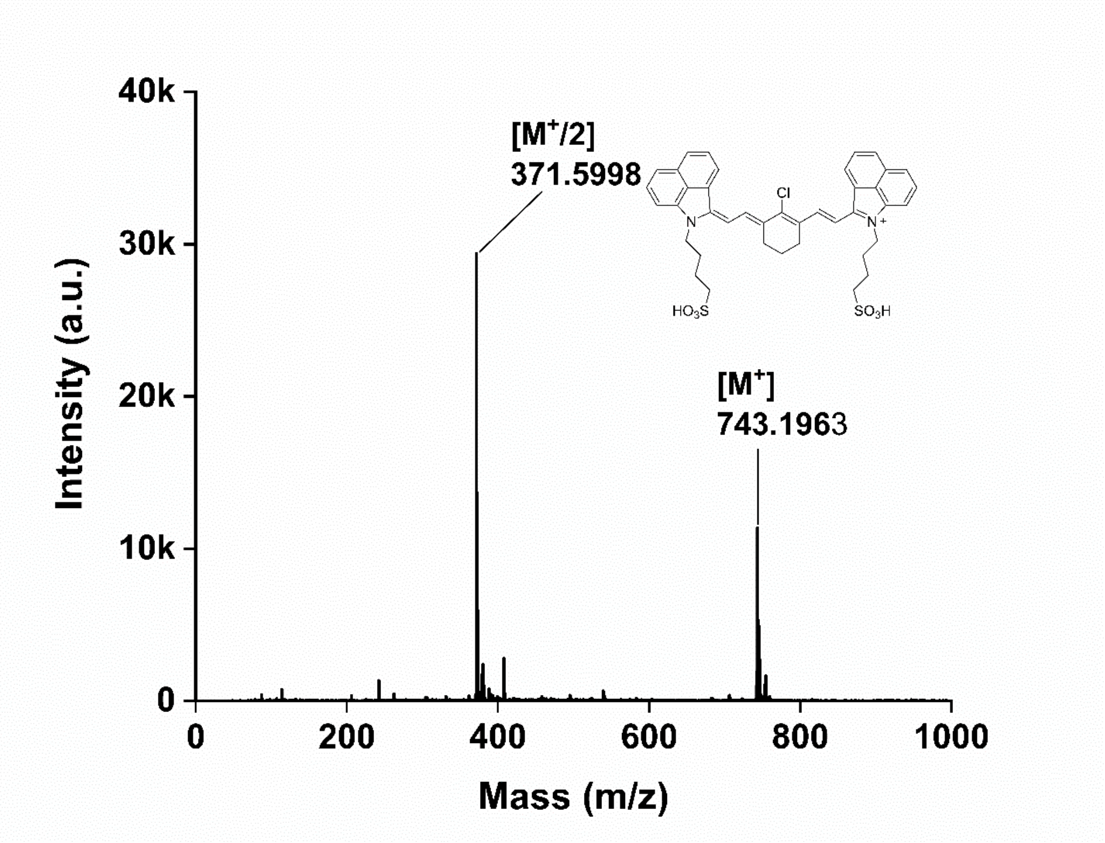

LC-HRMS spectra of the compound 13 (FD-1080-Cl).

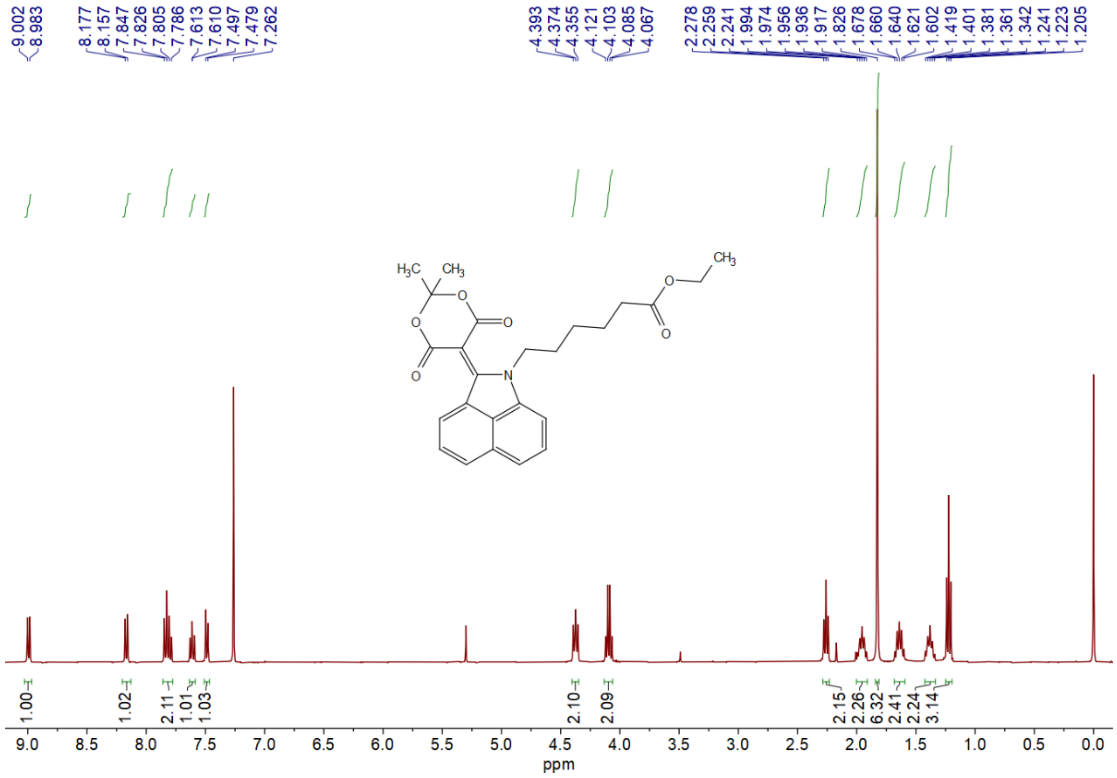

^{1}H-NMR spectrum of compound 14 in $CDCl_3$.

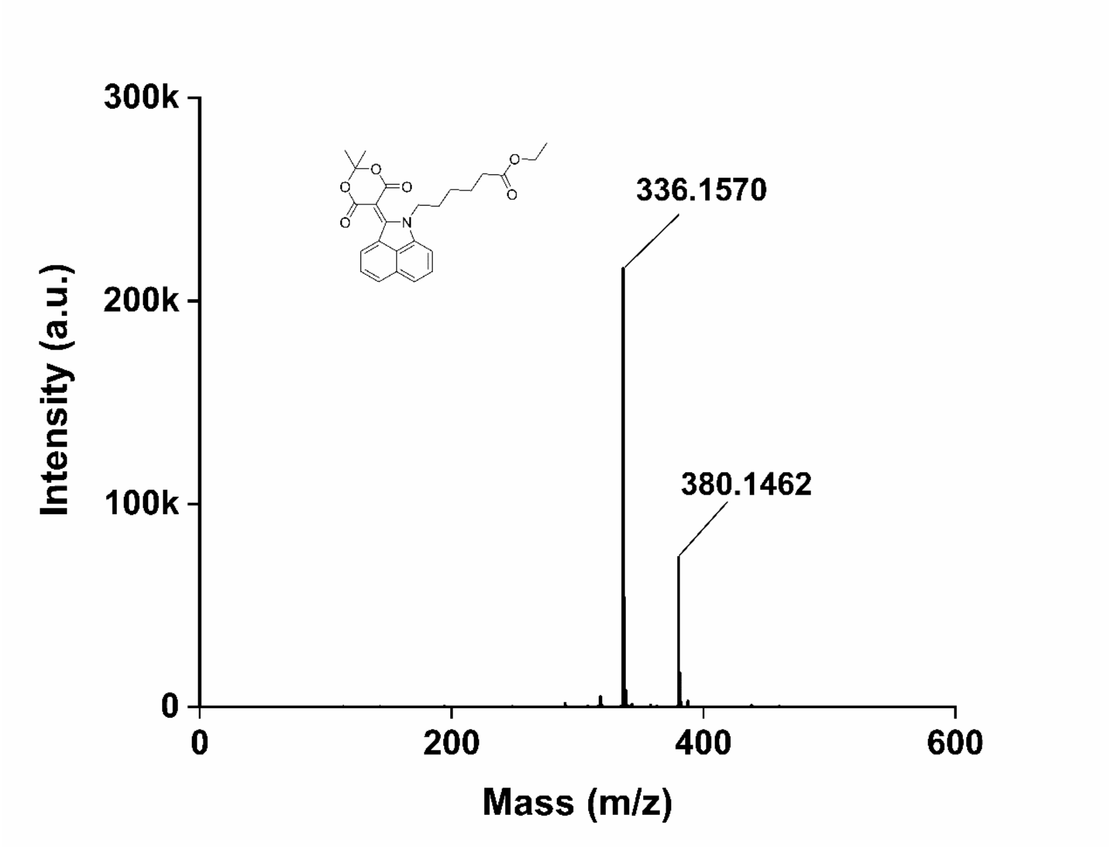

LC-HRMS spectra of the compound 14.

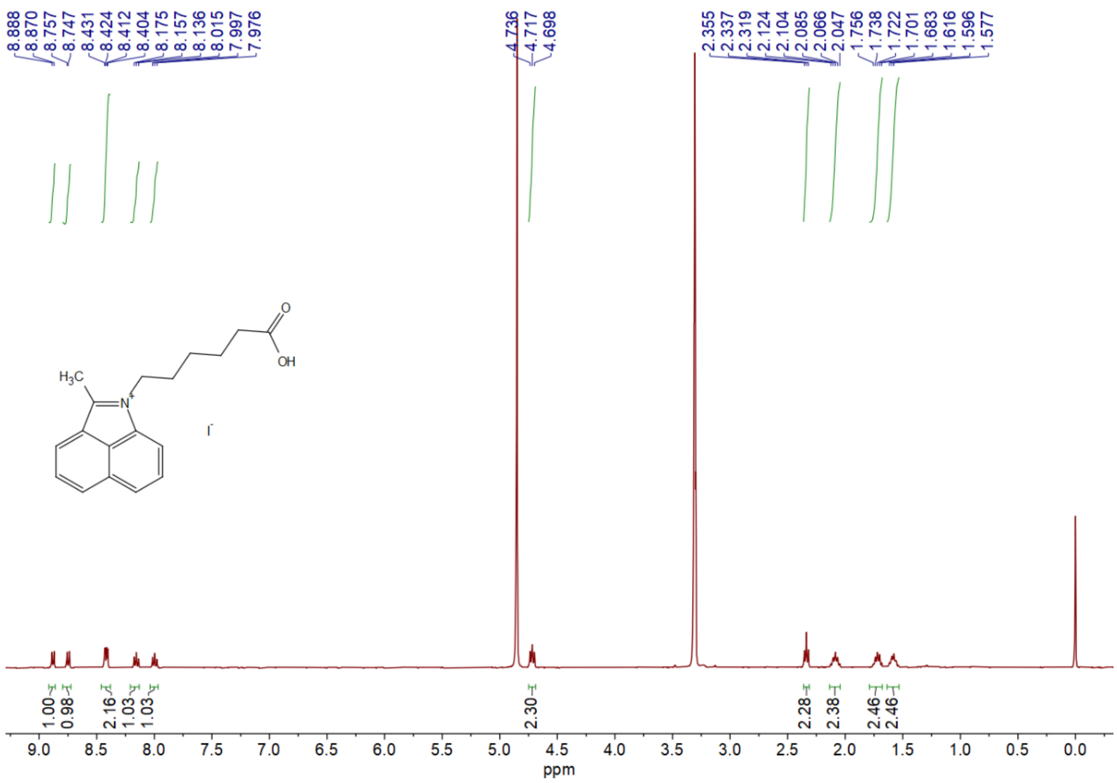

^{1}H-NMR spectrum of compound 15 in CD_3OD.

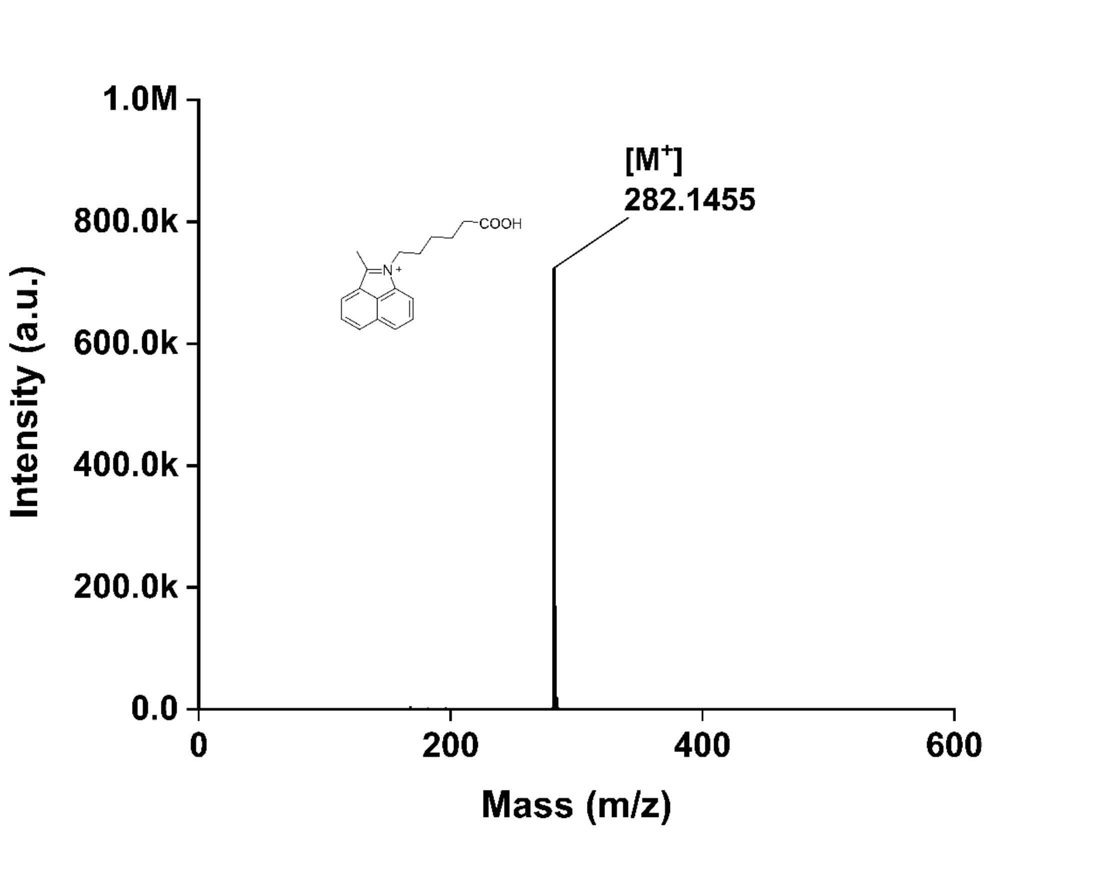

LC-HRMS spectra of the compound 15.

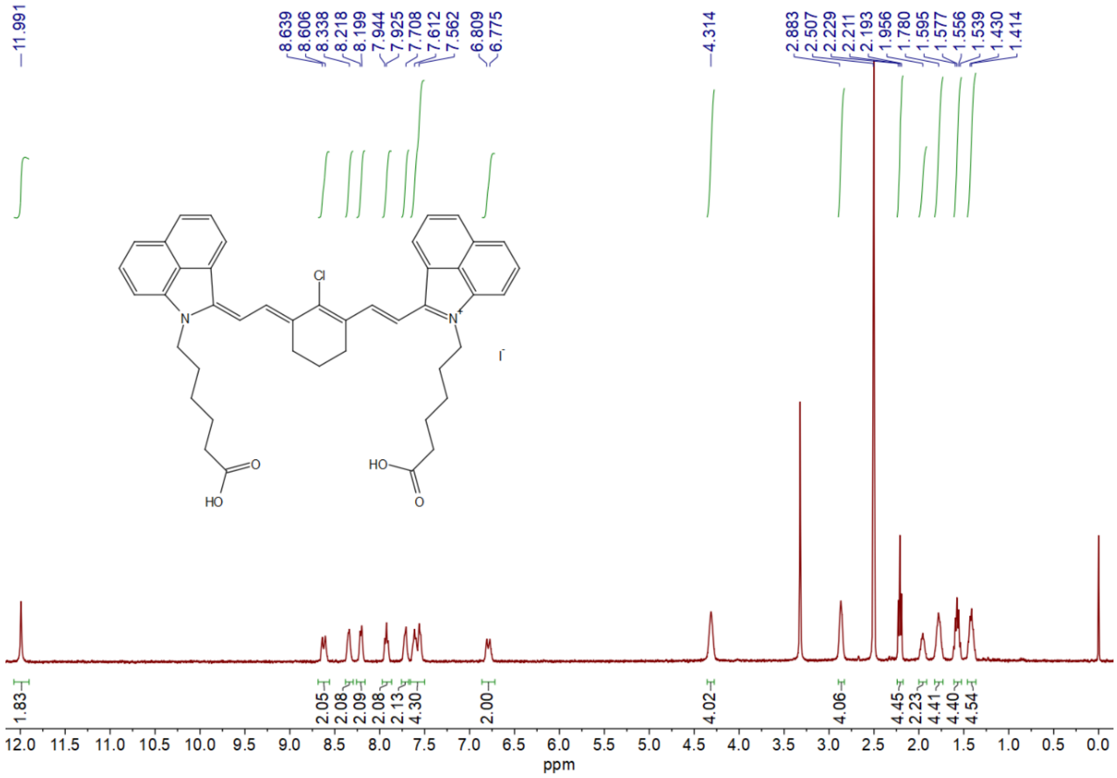

^{1}H-NMR spectrum of compound 16 in DMSO (CO-1080).

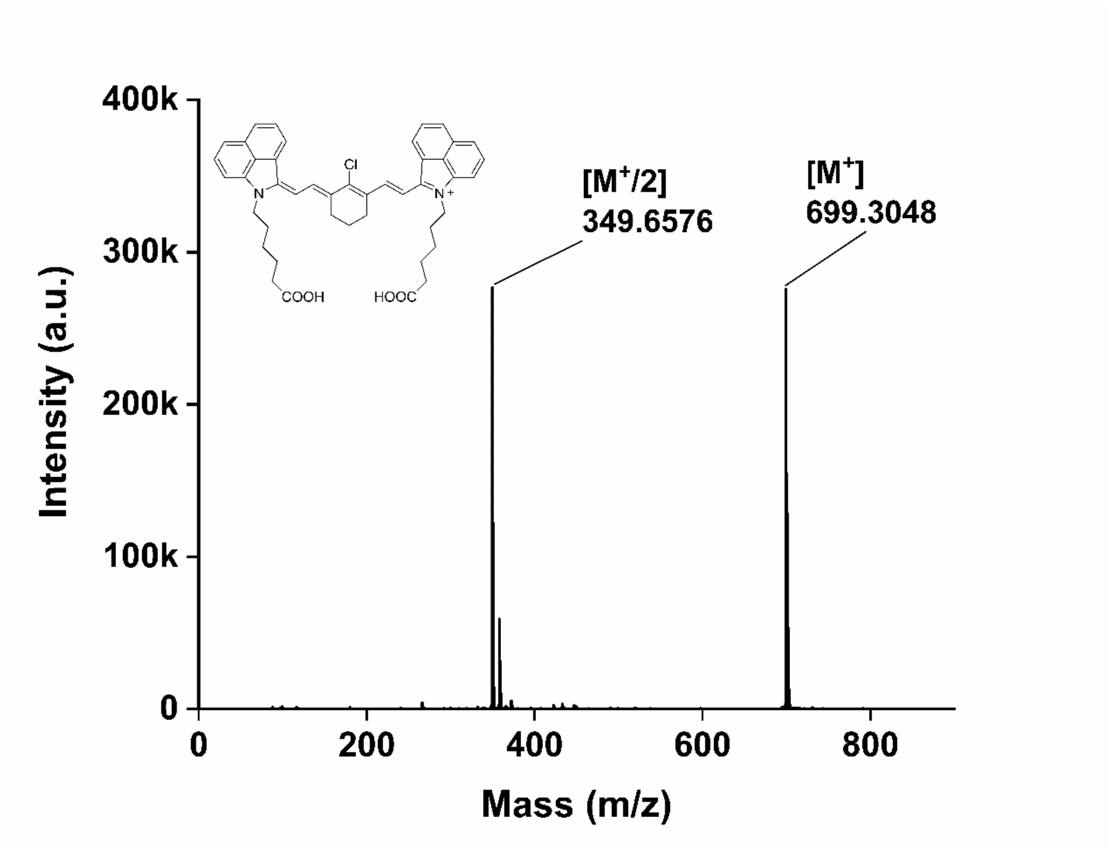

LC-HRMS spectra of the compound 16 (CO-1080).

Supporting references

[1] a) S. Chen, C. Ma, M.-S. Yuan, W. Wang, D.-E. Wang, S.-W. Chen, J. Wang, *RSC Adv.* **2016**, 6, 85529; b) L. Fu, Y. Huang, J. Hou, M. Sun, L. Wang, X. Wang, L. Chen, *J. Phys. Chem. B* **2022**, 10, 8432.

[2] E. R. Johnson, S. Keinan, P. Mori-Sánchez, J. Contreras-García, A. J. Cohen, W. Yang, *J. Am. Chem. Soc.* **2010**, 132, 6498.

[3] M. S. Valdés-Tresanco, M. E. Valdés-Tresanco, P. A. Valiente, E. Moreno, *J. Chem. Theory Comput.* **2021**, 17, 6281.

[4] S. H. Sinha, E. A. Owens, Y. Feng, Y. Yang, Y. Xie, Y. Tu, M. Henary, Y. G. Zheng, *Eur. J. Med. Chem.* **2012**, 54, 647.
